# Glucocappasalin Induces G2/M-Phase Arrest, Apoptosis, and Autophagy Pathways by Targeting CDK1 and PLK1 in Cervical Carcinoma Cells

**DOI:** 10.3389/fphar.2021.671138

**Published:** 2021-05-20

**Authors:** Guangya Xu, Xueling Yan, Zhongjia Hu, Lulu Zheng, Ke Ding, Yamei Zhang, Yi Qing, Tao Liu, Lijia Cheng, Zheng Shi

**Affiliations:** ^1^ Clinical Genetics Laboratory, Clinical Medical College, Affiliated Hospital and College of Basic Medicine and School of Pharmacy and School of Food and Biological Engineering, Chengdu University, Chengdu, China; ^2^ School of Pharmacy, Zunyi Medical University, Zunyi, China

**Keywords:** cervical cancer, GCP, CDK1/PLK1, cell cycle arrest, apoptosis, autophagy

## Abstract

Glucocappasalin (GCP), a natural product derived from the seeds of *Descurainia sophia* (L.) Webb. ex Prantl, exhibits potential antitumor activity in HeLa cervical carcinoma cells. In this study, we investigated the anti-cervical cancer property of GCP through the induction of cell cycle arrest, apoptosis, and autophagy *in vitro* and *in vivo*, and elucidated the underlying molecular mechanisms. We demonstrated that treatment with GCP inhibited the growth of HeLa, Siha, and Ca Ski cell lines in a dose-dependent manner, with HeLa cells displaying particular sensitivity to the GCP treatment. Subsequently, the expression of cyclin-dependent kinase 1 (CDK1) and polo like kinase 1 (PLK1) were evaluated in HeLa cells using the CDK1 kinase assay kit, the fluorescence polarization assay, real-time quantitative PCR, and western blotting. Our results demonstrate that GCP could be employed to attenuate the expression of CDK1 and PLK1 in a dose- and time-dependent manner. The complementary results obtained by flow cytometry and western blotting allowed us to postulate that GCP may exhibit its antitumor effects by inducing G2/M cell cycle arrest. Moreover, HeLa cells treated with GCP exhibited a loss in mitochondrial membrane potential, together with the activation of caspases 3 and 9, and poly ADP-ribose polymerase (PARP). Additionally, we found that GCP could increase the formation of acidic vesicular organelles (AVOs), as well as the levels of Beclin1, LC3-II, p62, and Atg5 proteins in HeLa cells. Further studies indicated that GCP triggered autophagy *via* the suppression of the PI3K/AKT/mTOR signaling pathways. The autophagy inhibitor 3-methyladenine (3-MA) was used to determine whether autophagy affects the apoptosis induced by GCP. Interestingly, the inhibition of autophagy attenuated apoptosis. *In vivo* anti-tumor experiments indicated that GCP (60 mg/kg, i.p.) markedly reduced the growth of HeLa xenografts in nude mice without apparent toxicity. Taken together, we demonstrate that GCP induces cell cycle G2/M-phase arrest, apoptosis, and autophagy by acting on the PI3K/AKT/mTOR signaling pathways in cervical carcinoma cells. Thus, GCP may represent a promising agent in the eradication of cervical cancer.

## Introduction

According to the WHO, cervical cancer is the fourth most common malignancy affecting women. Cervical cancer accounts for 12% of cervical cancer incident cases worldwide and for 11% of cervical cancer deaths in China (Siegel et al., 2020). Human papilloma virus (HPV) infection is considered as the principal etiological agent responsible for the onset of cervical carcinoma and is associated with 90% of reported cervical cancer cases (Cohen et al., 2019). Traditional strategies used in early-stage cervical carcinoma treatment, such as surgery, radiation treatment, and cytotoxic chemotherapy are widely employed. However, these strategies are not effective in the treatment of advanced local cervical cancer, as well as metastatic, and recurrent tumors (Wang et al., 2019). The occurrence and development of cervical cancer is a complex sequential multi-factorial process. Therefore, the most promising strategy for the treatment of cervical cancer should begin with the clarification of the molecular mechanisms involved in the onset and the development of cervical cancer, by revealing the critical signal transduction pathways and targeting specific molecules.

Cells adapt to the surrounding environment by establishing sophisticated cell cycle regulation mechanisms, whereby a large number of regulators ensure the efficiency of cell cycle progression (Mowers et al., 2017). Cell cycle dysregulation is therefore considered as one of the hallmarks of cellular transformation and cancer development (Zheng et al., 2019). Polo-like kinase 1 (PLK1), a member of the polo-like kinase (PLK) family, is an enzyme mainly involved in cell cycle progression. Cyclin-dependent kinase (CDK1) is a serine/threonine-like protein kinase. Together, PLK1 and CDK1 are essential cell cycle regulators, which play a vital role in promoting cell turnover by regulating cell differentiation and proliferation (Lemmens et al., 2018). PLK1 controls entry into mitosis and regulates the spindle checkpoint. The overexpression of PLK1 leads to oncogenesis as a result of chromosome instability through the disruption of checkpoint function (Chen et al., 2020). Previous studies have revealed that the knockdown of the gene encoding PLK1 significantly inhibited the proliferation of HeLa cells (Guo et al., 2018). PLK1 function was also shown to be associated with abnormalities in early cervical columnar epithelial cells. Moreover, multiple analyses of cancerous and healthy tissue samples derived from individuals with cervical cancer showed that PLK1 was overexpressed in cervical cancer tissues (Gao et al., 2020). Therefore, PLK1 presents an ideal target for the development of specific small molecule inhibitors for cervical cancer treatment. CDK1 is the only CDK of critical importance in the process of cell proliferation, and the abnormal expression of CDK1 has been observed in a variety of primary cancers (Asghar et al., 2015). CDK1 plays a complex role in the regulation of genetic networks involved in the progression of cervical cancer (Luo et al., 2016). To this end, CDK1 it is considered as a promising emerging target in the treatment of cervical cancer.

**FIGURE 1:**
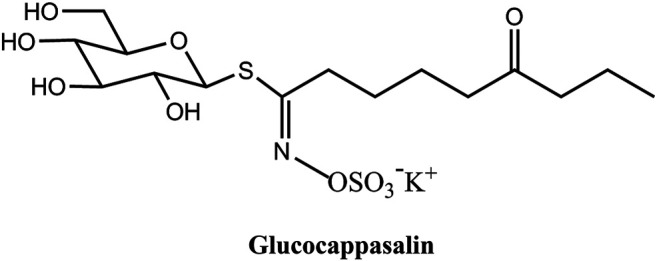
The chemical structure of Glucocappasalin (GCP).

Natural products are used in the treatment of various diseases, with many having the ability to control the growth of cancer cells. *Descurainia sophia* (L.) Webb. ex Prantl is widely distributed throughout the northeast of China, and its seeds are used in Chinese traditional medicine for the prevention and treatment of cardiovascular diseases, phlegm, cough, asthma, hyperlipidemia, and acute pharyngitis (Lee et al., 2013). Numerous studies have found that many active ingredients contained within *Descurainia sophia* seeds have significant anti-tumor effects, the use of which as a therapeutic agent is increasingly attracting the attention of researchers. Previous studies have proved that the ethanol extract of *Descurainia sophia* seeds has a potent cytotoxic effect on human lung cancer A549 cells (Sun et al., 2004; Kim et al., 2013). One of the products extracted from *Descurainia sophia* seeds is glucocappasalin (GCP), which has exhibited potential antitumor activities in HeLa cells (Khodarahmi et al., 2015). The chemical structure of GCP is shown in Figure 1. Previous studies have identified that GCP could potentially block the activities of CDK1/PLK1 by competing with ATP, potently blocking signal transduction (Shi et al., 2014). However, the underlying molecular mechanisms have not yet been investigated. The purpose of this study was to investigate the drug targets of GCP and its antitumor effects on cervical carcinoma cell both *in vitro* and *in vivo*, in addition to further characterizing the underling molecular mechanisms involved. To this end, we synthesized GCP and administered it to HeLa cells. Our previous results from the MTT assay suggested that GCP could significantly induce cervical cancer cell death, implying that GCP represents a promising antitumor agent against cervical cancer.

## Materials and Methods

### Reagents, Antibodies, Cell Lines, and Animals

GCP was synthesized as previously described (Cerniauskaite et al., 2011) and Supplementary Figure S1. The CCK-8 cell proliferation assay kit (KGA317), the JC-1 mitochondria staining kit (KGA602), the Annexin V-FITC/PI double staining cell apoptosis assay kit (KGA106), the cell cycle assay kit (KGA512), and the cell apoptosis acridine orange detection kit (KGA213) were obtained from KeyGEN BioTECH (Jiangsu, China). The MESACUP^®^ CDK1 kinase assay kit was purchased from MBL (Nagoya, Japan). The monodansylcadaverine (MDC) staining kit was purchased from Leagene (Beijing, China). The cDNA Synthesis Kit was sourced from Tiangen Biotech (Beijing, China). The MaximaTM SYBR Green Master Mix was obtained from Roche (Shanghai, China). 3-methyladenine (3-MA) was purchased from Mkbio (Shanghai, China). Antibodies against PLK1 (ab133442), Cleaved caspase-3 (ab2302), caspase-9 (ab2324), Bcl-2 (ab196495), Bcl-xl (ab77571), Cyclin B1 (ab72), CDK1 (ab133327), *p*-CDK1 (phospho Y15, ab254121), *p*-Cdc25c (phospho S216, ab47322), p27 (ab32034), p21 (ab109520), PARP (ab191217), LC3B (ab63817), Atg5 (ab108327), Beclin-1 (ab207612), p62 (ab91526), PI3K (ab140307), p-PI3K (phospho Y607, ab182651), AKT (ab8805), *p*-AKT (phospho T308, ab8933), *p*-mTOR (phospho S2448, ab109268), mTOR (ab32028), and GAPDH (ab8245) were purchased from Abcam (Cambridge, MA, United States).

Three human cervical cancer cell lines (Siha, HeLa, and Ca Ski) were purchased from American Type Culture Collection (ATCC, Rockville, MD, United States). The Siha (ATCC HTB-35) and HeLa (ATCC CCL-2) were cultured in DMEM medium (Hyclone, SH30022.01B), while Ca Ski cells (ATCC CRL-1550) were cultured in RPMI1640 medium (Hyclone, SH30809.01B), containing 10% fetal bovine serum (FBS, Hyclone, FB15011), penicillin (100 units/ml), and streptomycin (100 mg/ml). The cells were maintained in a humidified incubator at 37°C with 5% CO_2_ atmosphere.

Twenty-four female BALB/c nude mice (age: 6–8 weeks, weight: 18–22 g) were purchased from Sichuan Laboratory Animal Research Institute (Chengdu, China). All mice were housed in a standard SPF room, received humane care, kept free of specific pathogens, and provided sterile food and water. All animal procedures were conducted in compliance with the guidelines of the Institutional Animal Care and Treatment Committee of Chengdu University.

### Cell Viability Assay

Three human cervical cancer cell lines (HeLa, Siha, Ca Ski) were seeded into 96-well plates at a density of 5 × 10^3^ cells/well and cultured at 37°C and 5% CO_2_ for 24 h. After incubation, the samples were treated with different concentrations of GCP (0, 500, 1,000, and 1,500 nM) for 48 h, prior to recording the growth status of each group of cells *via* observation through a microscope. Subsequently, 10 µl of CCK-8 solution was added to each well of cells and incubated for an additional 2 h, as instructed in the Cell Counting Kit-8 (CCK-8) protocol, prior to measuring the absorbance at 450 nm on a microplate reader. Cell viability was calculated according to the formula: [average optical density (OD) of the experimental group/average OD of the control group] × 100%. Experiments were performed at least three times.

### Cell Cycle Assay

Cell cycle arrest was analyzed by flow cytometry. HeLa cells were seeded in 6-well plates (5 × 10^5^ cells/well) and cultured in an incubator at 37 with 5% CO_2._ When the cells adhered to the wall, various concentrations of GCP (0, 500, 1,000, or 1,500 nM) were added and the cells cultured for a further 48 h. After GCP treatment, the cells were washed twice with cold phosphate-buffered saline (PBS), 500 μl 75% ethanol was subsequently added and the cells were fixed at 4°C overnight. On the second day, the samples were stained with 400 μl propidium iodide (PI) and 100 μl RNase A, and incubated at room temperature and in the dark for 30 min. Fluorescence intensity was analyzed using CytoFLEX flow cytometry (Beckman Coulter, United States).

### CDK1 Kinase Assay

CDK1 activity was analyzed using the MESACUP^®^ CDK1 kinase assay kit, according to the manufacturer’s protocol. Briefly, exponentially growing HeLa cells (1 × 10^3^ cells/ml) were exposed to GCP. The cells were subsequently washed twice with ice-cold PBS and lysed on ice with lysis buffer. 10 μl of cell lysate, 5 μl of a biotin-labeled oligopeptide, and 5 μl of 1 mM ATP were mixed and incubated for 30 min at 30°C. Then, phosphor-MV peptide antibody was added to the wells and incubated at 25°C for 60 min. Subsequently, 50 μl peroxidase (POD)-streptavidin conjugate was added, and the cells cultured at 25°C for 5 min. The absorbance was measured at 492 nm. The assays were repeated three times independently.

### Fluorescence Polarization Assays

PLK1 is widely regarded as one of the most promising targets for cancer therapy due to its critical role in cell division and tumor cell survival. In additional to a highly conserved kinase domain, PLK1 also contains a unique Polo-Box domain (PBD), which is essential for PLK1’s subcellular localization and mitotic functions. The binding experiments were performed on a SpectraMax Multi-Mode Microplate Reader (Molecular Devices) using the 485 nm excitation and 535 nm emission filters. In the fluorescein polarization assays, fluorescence polarization (FP) was determined by measuring the parallel and perpendicular fluorescence intensities (Intparallel, F∥and Intperpendicular, F⊥, respectively). The percentage inhibition of the phosphopeptides at each concentration was defined as = 1- (Pobs − Pmin)/(Pmax − Pmin), where, Pmax was the polarization of the wells containing Plk1 PBD and the probe, Pmin referred to the polarization of the free probe, and Pobs was the polarization for the wells containing the inhibitors at a range of specified concentrations. In brief, 60 nM FITC-GPMQSpTPLNG-OH (30 μl/well) was added into the 384-well black plates. The Plk1 PBD protein was then added to the fluorescence polarization reaction solution (50 mMTris, pH 8.0, 10 mM NaCl, and 1 mM EDTA). The 384-well black plate was incubated at room temperature for 30 min with gentle shaking, prior to taking FP value measurements, made using a multifunctional microplate reader. All experiments were performed in triplicate.

### Apoptosis Analysis by Flow Cytometry

Apoptosis was determined using the Annexin V-FITC Apoptosis Detection Kit. Briefly, HeLa cells were seeded in a 6-well plate at a density of 5 × 10^5^ cells/well and cultured for 24 h. Then, GCP, at various concentrations ranging from 0 to 1,500 nM, was added to the cell cultures and incubated for a further 48 h. Subsequently, cells were collected and washed twice with cold PBS, prior to being resuspended in 500 μl binding buffer and stained with 5 μl Annexin V-FITC and 5 μl of PI for 30 min in the dark. Finally, cell apoptosis was detected by the flow cytometry.

### Apoptosis Analysis by TUNEL Staining

The TUNEL apoptosis *in situ* assay kit was used to detect the effect of GCP on HeLa cell apoptosis. HeLa cells at the logarithmic growth stage were taken and inoculated into a 6-well plate at a density of 1 × 10^6^ cells/well. The cells were cultured overnight at 37°C, 5% CO_2_. In the control group, an equal volume of culture medium was added. In the low-dose group, 500 nM GCP was added. 1,000 nM GCP was administered to the medium-dose group, while the high-dose group received 1,500 nM GCP. Cells in each group were cultured at 37°C, 5% CO_2_ for 24 h, rinsed with PBS for 5 min and fixed with 4% poly (methanol) for 30 min 50 μl TUNEL assay reagents were added, followed by incubation at 37°C in the dark for 1 h. The cells were subsequently washed with PBS for 5 min and then stained with 0.05 μg/μl DAPI for 10 min. Finally, the Olympus BX53 fluorescence microscope was used to observe the cells.

### Mitochondrial Membrane Potential Assay

The JC-1 mitochondria staining kit was used to measure the mitochondrial membrane potential. The cells in the logarithmic growth phase were seeded into a 6-well plate at a density of 5 × 10^5^ cells/mL, and cultured in a 37°C, 5% CO_2_ incubator for 24 h, prior to being treated with different concentrations of GCP for 48 h. 500 μl 1 × incubation buffer, prepared according to the manufacturer’s instructions, was subsequently added to the cells, followed by 1 μl JC-1 solution. The cells were mixed and incubated at 37°C and 5% CO_2_ for 20 min. During the process, the cells were inverted every 5 min. Finally, the fluorescence intensity was analyzed on a flow cytometry.

### Acridine Orange Staining

Acidic vesicle organelles (AVOs) are hallmarks of autophagy (Thome et al., 2016). Acridine orange (AO) is an auto-fluorescent agent. When autophagy occurs, the cytoplasm and nucleolus of AO-stained cells exhibit bright green fluorescence, while the AVOs glow bright orange/red. The intensity of the red fluorescence is proportional to the degree of acidity. Briefly, according to the manufacturer’s instructions, cells were incubated with GCP at different concentrations and then resuspended with 1 × Buffer A. 95 μl of cell suspension and 5 μl of AO dye were mixed and stained for 20 min at room temperature in the dark. After washing with PBS, the green fluorescence was detected at the maximum excitation/emission wavelengths of 502/525 nm, and red fluorescence was detected at 460/650 nm. The images of cells containing AVOs were photographed with a laser confocal microscope (Olympus).

### Monodansylcadaverine Staining

Autophagy was quantified using a monodansylcadaverine (MDC) staining kit. HeLa cells were seeded into 6-well culture plates at a density of 5 × 10^5^ cells/well cultured for 24 h at 37°C. After incubation for 48 h, cells were stained with MDC for 15 min at room temperature in the dark. Subsequently, the fluorescence intensity of each sample was evaluated by flow cytometry, which gave a measure of extent of autophagy in the cells.

### Quantitative Real-Time PCR Analysis

The expression levels of CDK1 and PLK1 after GCP treatment at different time points (24, 48, and 72 h) were analyzed by quantitative real-time PCR (qRT-PCR). Total RNA was extracted from HeLa cells with TRIzol reagent, following the manufacturer’s protocol. The extracted RNA was treated with DNase I to remove the remaining DNA. Then, the MiRcute First-strand cDNA Synthesis Kit was used to reverse transcribe RNA to cDNA. qRT-PCR analysis was carried out using the MaximaTM SYBR Green Master Mix on a 7,500 real-time PCR system according to the manufacturer’s protocol (Applied Biosystems, United States). The PCR cycling parameters (19 cycles) were as follows: denaturation (94°C, 30 s), annealing (56°C, 30 s), and extension (72°C, 30 s). GAPDH (glyceraldehyde-3-phosphate dehydrogenase) was used as the internal standard control. At the end of the PCR reaction, a melting curve was established, and the gene expression was calculated using the 2^−ΔΔCt^ method. Each sample was assayed in triplicate. The primer sequences, which were obtained from PrimerBank, are shown in Table 1.

**TABLE 1:** Primer sequences for the RT-PCR.

Gene	Primer	Sequence
CDK1	Forward	5′-GGG TCA GCT CGC TAC TCA AC-3′
	Reverse	5′-AAG TTT TTG ACG TGG GAT GC-3′
PLK1	Forward	5′-GGA CTA GTT AGC TGC CCT CCC CTC CG-3′
	Reverse	5′-CCC AAG CTT GAA TAT TCA CAT CTG TTT AAT-3′
GAPDH	Forward	5′-CTG GGC TAC ACT GAG CAC C-3′
	Reverse	5′-AAG TGG TCG TTG AGG GCA ATG-3′

### Western Blotting Analysis

The HeLa cells, treated with different concentrations of GCP, were collected and lysed on ice with RIPA buffer for 30 min to extract the total protein. Protein quantification was performed using a BCA protein assay kit. The protein was loaded on a 10% sodium dodecyl sulfate-polyacrylamide (SDS-PAGE) gel for protein separation, and then transferred onto the polyvinylidene difluorid (PVDF) membrane. After transfer, the membrane was rinsed with PBS for 1–2 min, and then sealed with 5% skim milk at 4°C for 2 h. Following the addition of the primary antibody (1:1,000), the membrane was incubated overnight at 4°C. After washing with PBS several times, we added the secondary antibody conjugated to horseradish peroxidase (HRP) and incubated at room temperature for 1.5 h. Finally, the western blot detection system was used to image the protein bands, and ImageJ software was used to analyze the optical density of the bands. GAPDH was used as an internal control. All experiments were repeated three times independently.

### Xenograft Model

A subcutaneous model of cervical cancer was established using HeLa cells. On day 0, 100 μl cell suspension of ∼1 × 10^6^ HeLa cells were subcutaneously injected into the right upper flank of the mice. Animals started treatment when the average tumor volume reached 100 mm^3^. Mice were randomly divided into four groups (*n* = 6). Every 2 days, either normal saline, the positive control drug (cisplatin), medium alone (30 mg/kg), or a high (60 mg/kg) dose of GCP, were injected through the tail vein. To observe the dynamic changes in tumor growth, we measured the tumor size every 2 days and recorded the long diameter 1) and short diameter 2) of the tumor with calipers. The tumor volume (V) was calculated using the following formula: a*b^2^/2. After 10 doses, the mice were sacrificed. The complete tumor tissue and various major organs (heart, liver, spleen, lung, kidney, etc.) were removed. Each tissue sample was accurately weighed, before the parts were cut up and fixed with 4% formaldehyde solution to be used for follow-up research.

### Histological Staining

The tissues were removed from the fixing solution, dehydrated, embedded in paraffin, and cut into 5 μm sections. Sections were sliced for H&E and TUNEL staining. Histopathology analysis was performed by staining the paraffin sections of tumors with H&E in accordance with the manufacturer’s protocol. Briefly, the samples were stained with the hematoxylin solution for 10 min, and then placed in the eosin solution for 1 min. Finally, all tissue slices were imaged under the microscope and analyzed using image analysis software.

### Statistical Analyses

Data were analyzed by ANOVA and expressed as means ± standard deviation ($\bar{x}\pm s$) (SPSS 22.0, United States). A *p* < 0.05 was considered statistically significant (*p < 0.05, **p < 0.01, ***p < 0.001).

## Results

### GCP Inhibits the Growth of HeLa, Siha, and Ca Ski Cells

**FIGURE 2:**
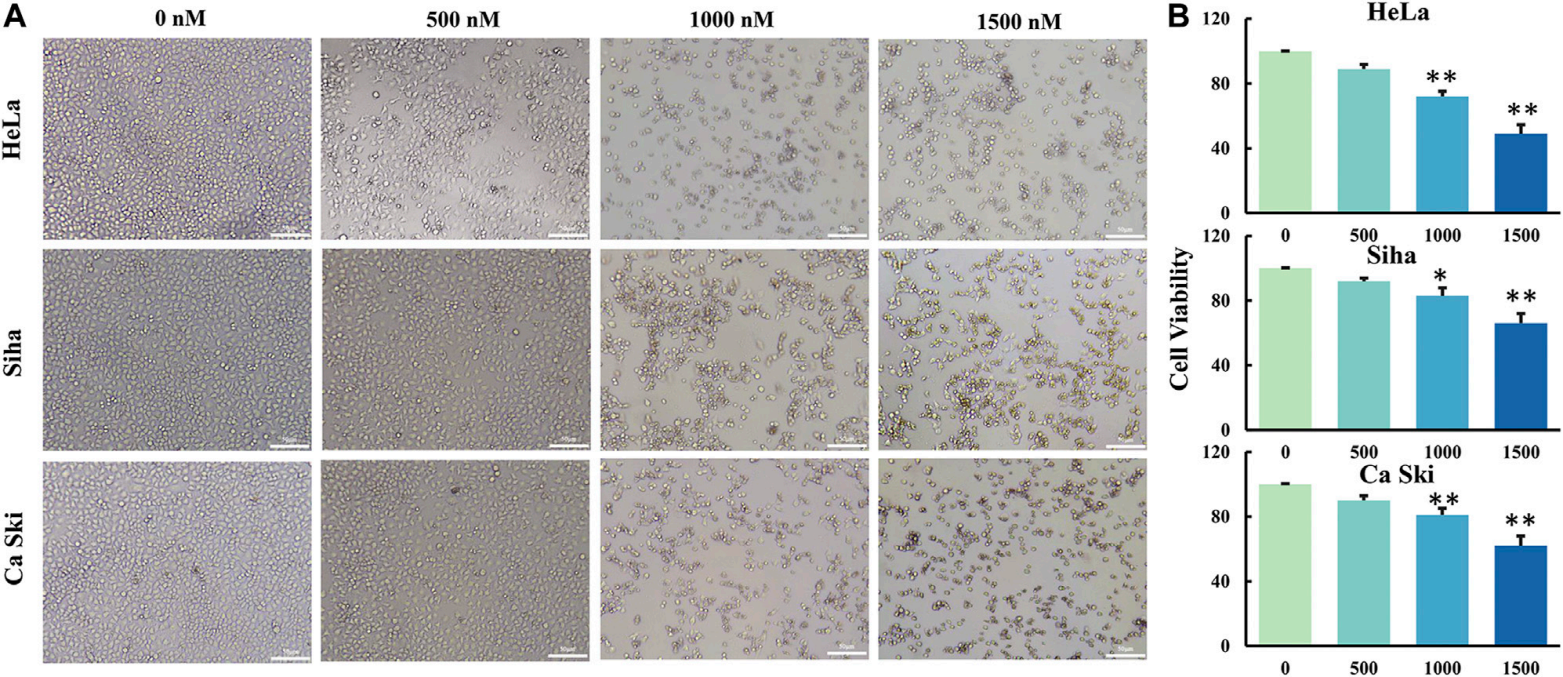
GCP inhibits cell growth of three human cervical cancer cell lines. **(A)** Hela, Siha and Ca Ski cells were treated with different dose of GCP for 48 h, cell morphology was observed using a microscope (Magnification bars, 50 μm). **(B)** The cell viability was detected by CCK8 assay. Each bar represents the mean ± SD of three independent experiments. *p < 0.05, **p < 0.01 vs control.

Two methods were used to measure the cell viability of three different cervical cancer cell lines treated with GCP. Firstly, to observe the cell death of cervical cancer cells treated with different doses of GCP, the morphologies of cancer cells were compared to those of untreated control cells using light microscopy. As shown in Figure 2A, we observed a lot of floating cells and cellular debris, in addition to changes to the cell membrane (deformed or incomplete), and the appearance of vacuoles and intracellular particles. Secondly, the CCK8 assay was used to quantitatively measure the cell viability of the three cancer cell lines after GCP treatment (Figure 2B). Collectively, these results indicate that GCP could significantly reduce the activity of cervical cancer cell lines and inhibit their cell proliferation in a dose-dependent manner, with the greatest effect of GCP being seen in HeLa cells. Based on these results, we used the HeLa cell line for further investigation.

### GCP Targets PLK1 and CDK1 to Inhibit Their Expression in HeLa Cells

**FIGURE 3:**
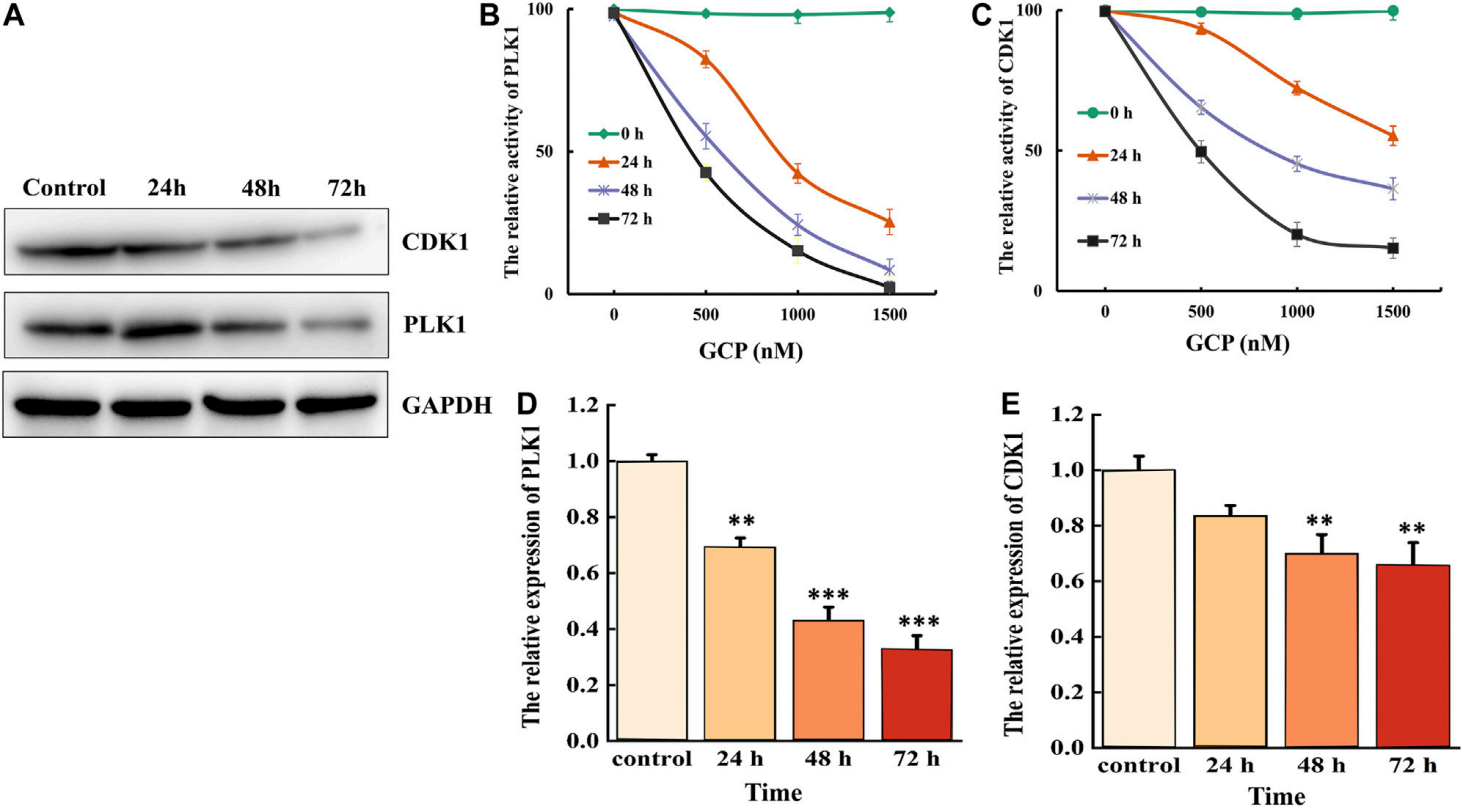
GCP inhibits the expression of CDK1 and PLK1. HeLa cells were treated with 1,500 nM for 24–72 h, **(A)** western bolt analysis the expression of CDK1 and PLK1. GAPDH was used as internal control. **(B)** Biological activity of PLK1 PBD protein was measured by Fluorescence polarization assays. **(C)** CDK1 activity was examined after incubation with different GCP doses and for different lengths of time using a kit. **(D, E)** The relative expression of PLK1 and CDK1 was evaluated by RT-qPCR analysis.

**FIGURE 4:**
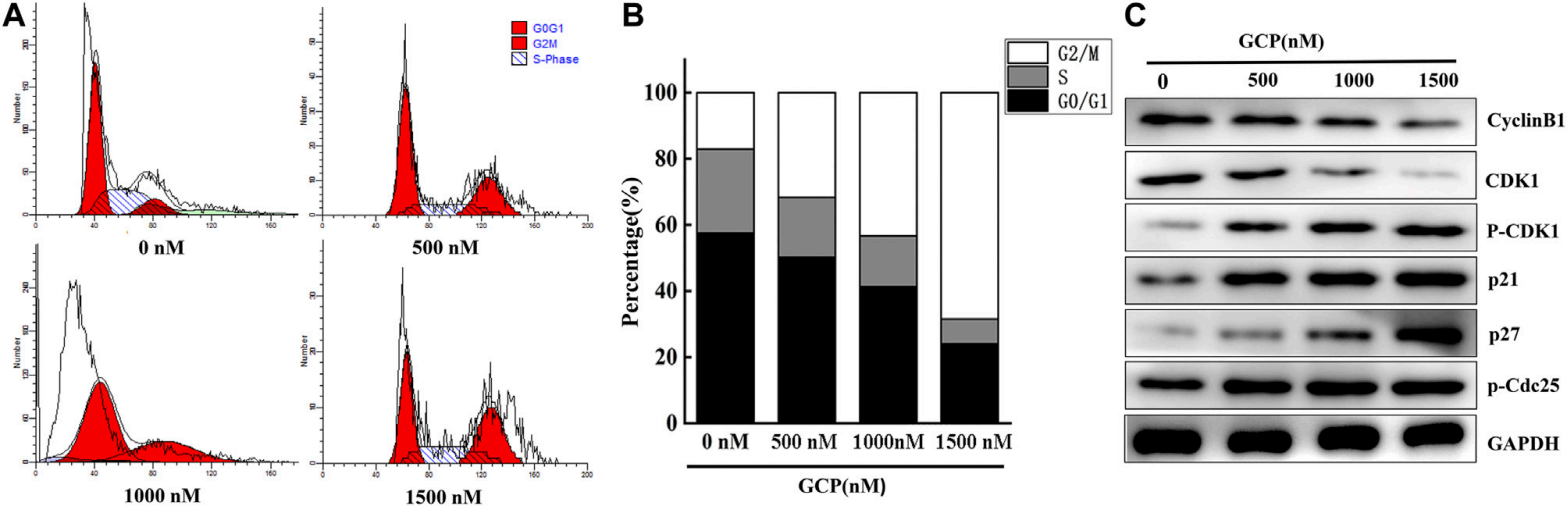
GCP induces cell cycle G2/M arrest in HeLa cells. **(A, B)** The HeLa cells were treated with different dose of GCP for 48 h, and the distribution of cell cycle was assessed by flow cytometry. The percentage of cells in each phase is shown as mean ± SD from three independent experiments. **(C)** The expression of related protein was analyzed by western bolt.

CDK1 and PLK1 are critical cellular regulators, which play a vital role in cell differentiation, proliferation, and autophagy. Therefore, we examined the effect of 1,500 nM GCP on the protein expression levels of CDK1 and PLK1 at 24, 48, and 72 h in HeLa cells by western blotting. As shown in Figure 3A, a time-dependent decrease in CDK1 and PLK1 expression was observed. CDK1 activity was subsequently measured using the CDK1 kinase assay kit, and the activity of PLK1 PBD was measured by fluorescence polarization. The results demonstrated that the expression levels of both CDK1 and PLK1 were markedly reduced in a time- and dose-dependent manner following GCP treatment (Figures 3B, 4C). PLK1 and CDK1 mRNA levels, assessed by qRT-PCR, were significantly lower in cells treated with GCP in a time- and dose-dependent manner. Furthermore, 72 h after GCP treatment, the relative expression of PLK1 fell from 1−0.28, and the expression of CDK1 decreased to 0.33 (Figures 3D,E). The results obtained with qRT-PCR here were largely consistent with the western blot data. Collectively, these data indicate that CDK1 and PLK1 represent potential therapeutic targets of GCP in the treatment of cervical cancer.

### GCP Induces Cell Cycle G2/M Arrest in HeLa Cells

The effect of GCP on cell cycle phase distribution was studied in HeLa cells by flow cytometry using propidium iodide (PI) staining (Figure 4A). When HeLa cells were treated with different concentrations of GCP for 24 h, the results demonstrated that the percentage of cells in the G2/M phase increased significantly (18–66%), accompanied by a decrease in the percentage of cells in the S and G0/G1 phases (Figure 4B). The expression of cell cycle-related proteins was subsequently measured by western blotting in order to elucidate the mechanisms involved. We found that the expression of Cyclin B1 and CDK1 was downregulated, while the levels of phospho-CDK1, p21, and p27 were significantly upregulated in a dose-dependent manner. Moreover, compared with the control group, the expression of phospho-Cdc25 slightly increased (Figure 4C). These data indicate that GCP triggers G2/M-phase arrest by regulating cell cycle-specific proteins.

### GCP Induces Apoptosis *via* the Mitochondrial Pathway in HeLa Cells

**FIGURE 5:**
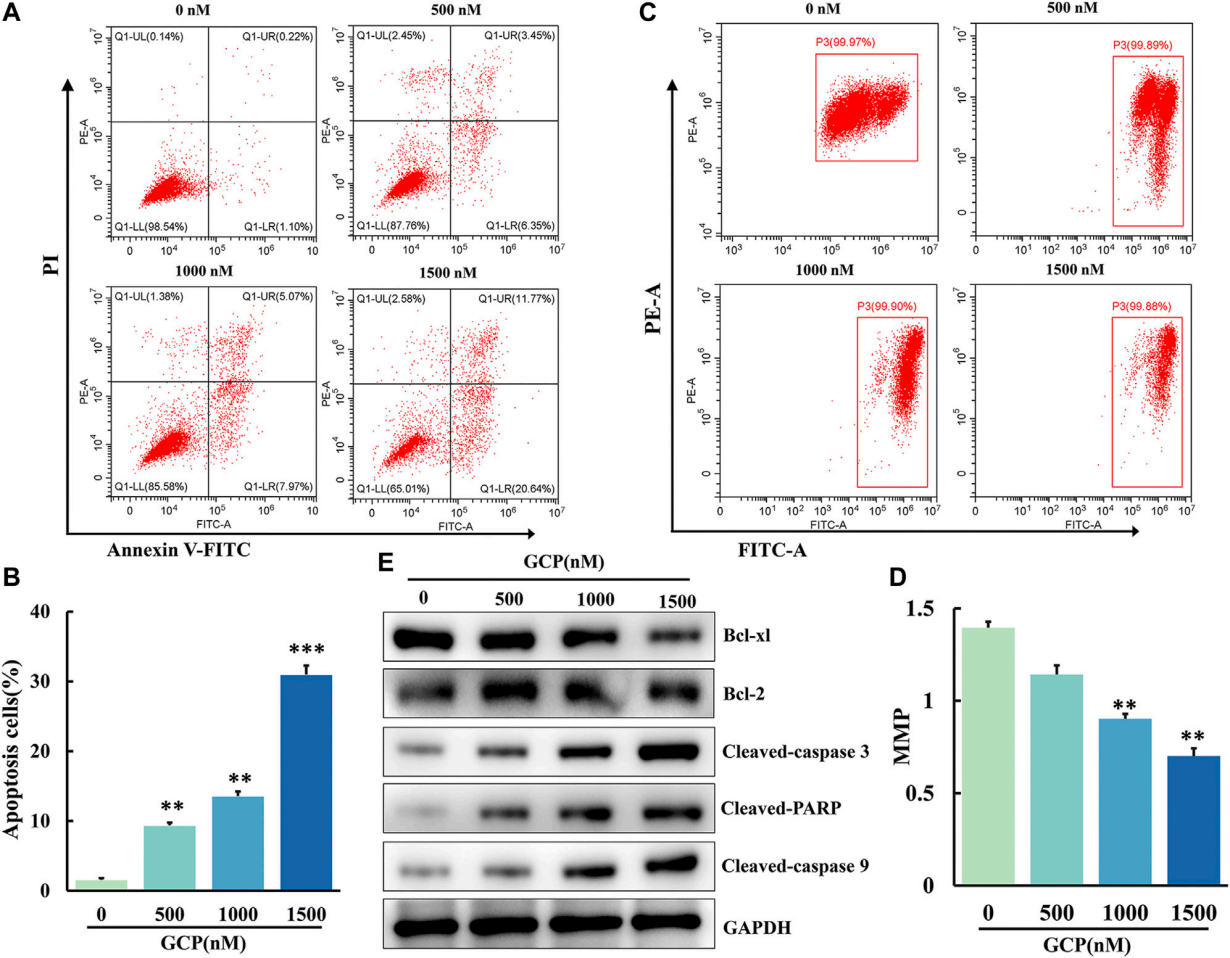
GCP induces apoptosis by mitochondrial pathway in HeLa cells. **(A, B)** AV/PI flow cytometry was used to detect the effect of GCP on the apoptosis. The percentage of apoptosis cells is shown as mean ± SD from three independent experiments. **(C, D)** The mitochondrial membrane potential after GCP treatment were measured using JC-1 staining by flow cytometry. Each bar represents as mean ± SD from three independent experiments. *p < 0.05, **p < 0.01, ***p < 0.001 vs control. **(E)** Apoptosis-related protein expression (Bcl-xl, Bcl-2, Cleaved-caspase 3, Cleaved PARP, Cleaved-caspase 9) in HeLa cells after GCP treatment at 0, 500, 1,000, 1500 nM for 48 h was analyzed by western blotting.

To investigate the apoptotic effect of GCP on HeLa cells, apoptosis was analyzed by means of Annexin V-FITC and PI staining using flow cytometry. We observed that the number of cells in early or late apoptosis increased in a dose-dependent manner (Figure 5A). Furthermore, the percentage of total apoptotic cells increased from 9.8 ± 0.13% to 32.4 ± 0.34% following GCP treatment (Figure 5B). This increase was statistically significant (**p < 0.01, ***p < 0.001). The process of cell apoptosis is often accompanied by the destruction of the mitochondrial transmembrane potential. Therefore, to observe the effect of GCP on the mitochondrial signaling pathway, we used the fluorescent mitochondrial probe JC-1 to measure mitochondrial membrane potential (MMP; Δψm). Mitochondrial membrane potential was calculated using the following formula: MMP = fluorescence intensity of PE/fluorescence intensity of FITC. As shown in Figures 5C,D, the MMP decreased significantly (1.46–0.70) following GCP treatment. Afterward, the expression of apoptosis-related proteins in the cells was measured by western blotting. GCP could markedly increase the levels of cleaved caspase-3, cleaved caspase-9, and cleaved PARP, while decreasing the expression of the anti-apoptotic proteins Bcl-2 and Bcl-xL (Figure 5E). In brief, these results indicated that GCP induces apoptosis through the mitochondrial pathway.

### GCP Induces Autophagy in HeLa Cells *via* the AKT/mTOR Signaling Pathway

**FIGURE 6:**
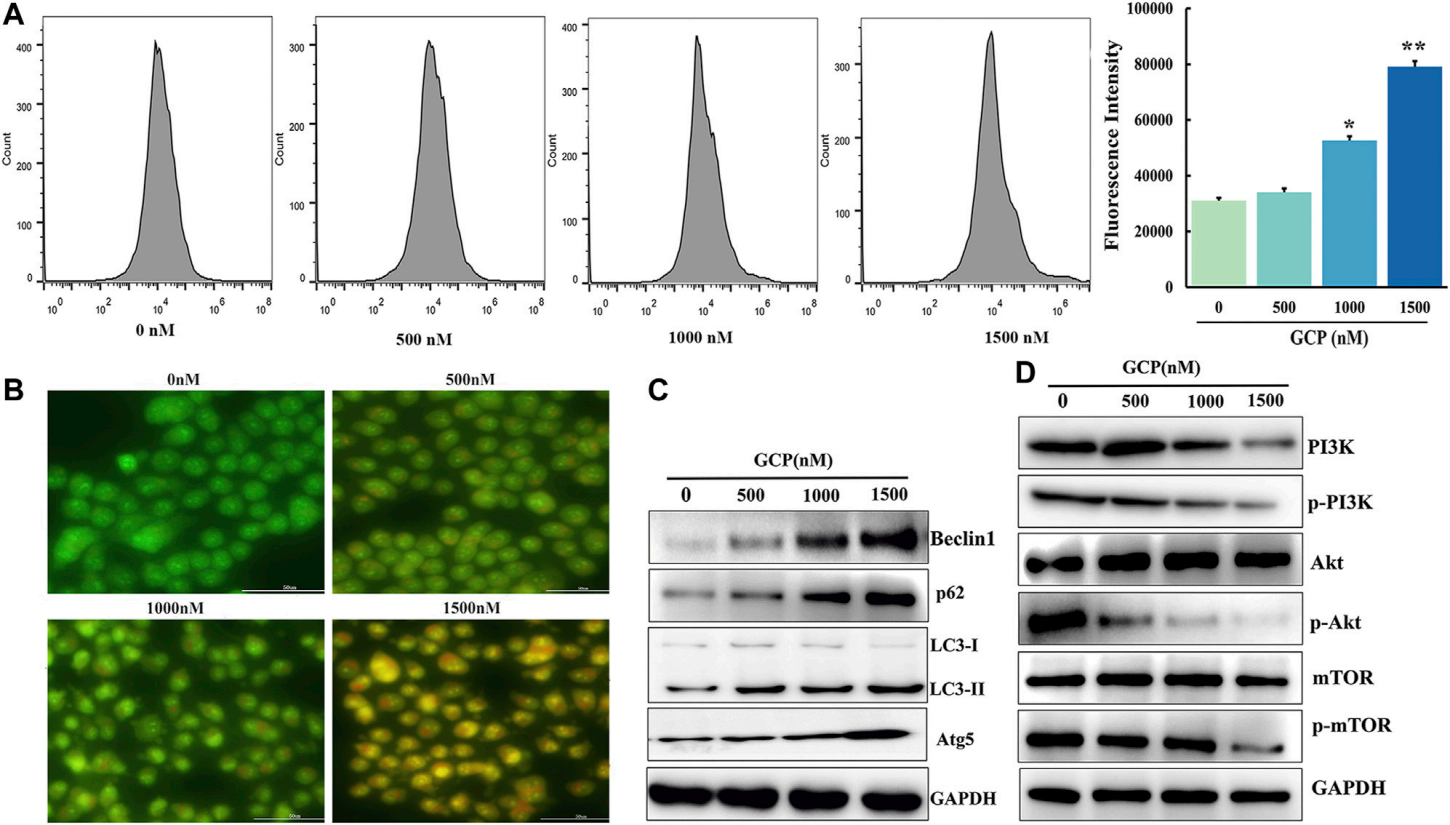
GCP induces autophagy in HeLa cells *via* the PI3K/Akt/mTOR signaling pathway. **(A)** Flow cytometry assay to detect autophagy level using by MDC staining, *p < 0.05, **p < 0.01 vs control. **(B)** Acridine orange staining was used to detect autophagic vacuoles. **(C)** The level of autophagy-related protein and **(D)** PI3K/Akt/mTOR signaling pathway protein expression shown by western blot analysis.

Autophagy is considered to be a dynamic process involving the phagocytosis and degradation of autologous cytoplasmic proteins or organelles, which plays a major role in cancer progression (Ferro et al., 2020). To examine whether GCP affected autophagy, AVOs were stained with AO and analyzed on a laser confocal microscope. As is evident from Figure 6B, the number of orange vesicles was highly increased following GCP treatment. Subsequently, flow cytometry was utilized to monitor the fluorescence intensity of HeLa cells stained by MDC. The mean fluorescence intensities of the control, low dose, medium dose, and high dose groups were 31,064, 34,044, 52,593, and 79,100, respectively (Figure 6A). Next, the expression of the autophagy-related markers Beclin1, LC3, p62, and Atg5 were detected by western blotting. As shown in Figure 6C, we also observed that GCP treatment increased the expression of Beclin1, LC3, p62, and Atg5 in HeLa cells in a dose-dependent manner. The above results suggest that GCP indeed induces autophagy.

Since the role of mTOR, as a crucial molecule in autophagy, is well documented, we further explored whether the AKT/mTOR signaling pathway is involved in GCP-induced autophagy. To this end, 48 h following GCP treatment of HeLa cells, western blotting was used to measure the expression levels of total AKT, phospho-AKT, total mTOR, and phospho-mTOR proteins. As shown in Figure 6D, the expression of total AKT and mTOR proteins in the GCP treatment group was not significantly altered, while the expression of *p*-AKT and *p*-mTOR was reduced in a dose-dependent manner, relative to the control group. These results indicate that GCP induces autophagy by regulating the AKT/mTOR signaling pathway in HeLa cells.

### Inhibition of Autophagy Suppresses GCP-Induced Apoptosis

**FIGURE 7:**
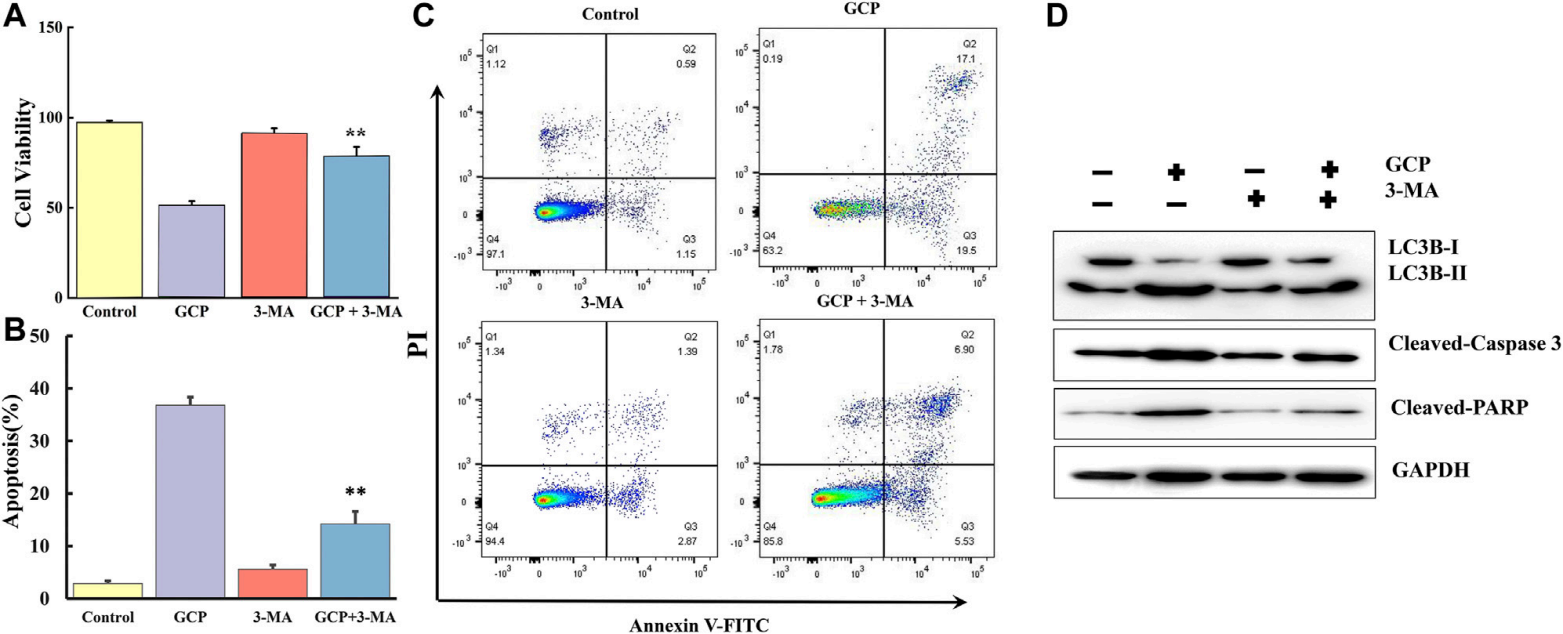
Inhibition of autophagy suppresses apoptosis induced by GCP. HeLa cells were preincubated with or without 3-MA for 2 h, then treated with GCP (1500 nM) for 24 h. **(A)** The CCK8 assay was used to measure the cell viability. **(B)** The levels of LC3, Cleaved-caspase 3, Cleaved PARP were detected by western bolt analysis. **(C, D)** Detection of apoptosis by flow cytometry. Each bar represents as mean ± SD from three independent experiments. **p < 0.01 vs control.

3-MA, a specific inhibitor of autophagy, was used to further clarify the interplay between apoptosis and autophagy induced by GCP in HeLa cells. The CCK8 assay was performed in HeLa cells treated with 1,500 nM GCP for 24 h in the absence or presence of 3-MA (2 mM). 3-MA alone had no significant effect on cell viability, relative to the control group. However, treatment with 3-MA improved cell viability by ∼26% compared to that observed for cells exposed to GCP alone (Figure 7A). We then examined the extent of apoptosis using the Annexin V-FITC apoptosis detection kit. Flow cytometry results (Figures 7B,C) indicated that the GCP-induced (1,500 nM) apoptosis rate was 36.8 ± 1.56%, while in the GCP+3-MA group the rate of apoptosis was 14.2 ± 2.36%. Correspondingly, compared with the GCP-treated cells, 3-MA lowered the protein levels of cleaved caspase-3 and PARP to a certain extent (Figure 7D). These data reveal that autophagy induced by GCP serves a pro-death function. However, there was still a difference between the GCP+3-MA and control groups, suggesting that there may be other factors that cause apoptosis, such as the intrinsic mitochondrial pathway.

### GCP Inhibits Tumor Growth *In Vivo*

**FIGURE 8:**
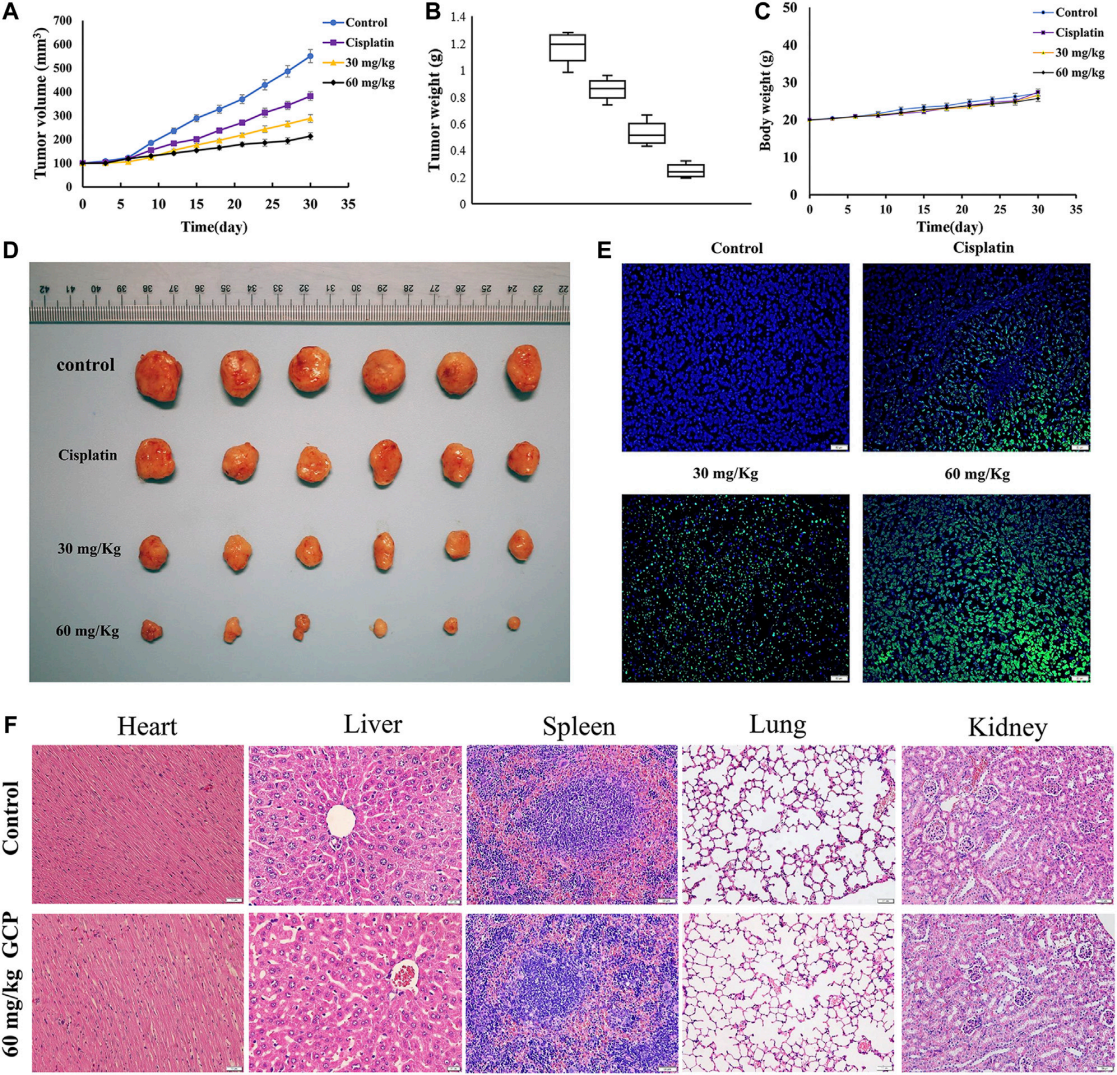
GCP inhibits tumor growth *in vivo*. **(A)** Tumor growth curves **(B)** tumor weight **(C)** and mouse body weights in four groups (control group; cisplatin group; 30 mg/kg GCP group; 60 mg/kg GCP group). **(D)** Image of tumors from mice of each group. **(E)** Tumor tissues were stained using TUNEL method, and the green area is TUNEL-positive cells (Magnification bars, 20 μm). **(F)** HE staining of heart, liver, spleen, lung, kidney in control and 60 mg/kg GCP group (Magnification bars, 20 μm).

We established the BALB/c nude murine xenograft model to further confirm the antitumor effect of GCP *in vivo* with cisplatin as the positive control. Firstly, the tumor volumes and weights were measured (Figures 8A–C). The tumor volume and weight in the control group were 550.8 mm^3^ and 1.22 g, respectively, while those in the 60 mg/kg GCP group were 213.4 mm^3^ and 0.23 g. As depicted in Figure 8D, significantly smaller tumor sizes were observed in the 30 and 60 mg/kg GCP treatment groups (especially in the 60 mg/kg GCP, *p* < 0.001), relative to the positive control and saline groups. In order to observe the extent of apoptosis, TUNEL staining was performed on tumor tissues from each group. TUNEL-positive cells were stained green, and the nuclei were labeled with DAPI (blue) in the merged image (Figure 8E). The highest level of green light intensity was observed in the high-dose GCP treatment group, which indicates that GCP is effective in suppressing tumor growth *in vivo* by inducing apoptosis. Finally, H&E staining was used to examine pathological changes affecting vital organ tissues (the heart, liver, spleen, lung, and kidney). The results shown in Figure 8F revealed that no significant organ toxicities were observed in the mice treated with 60 mg/kg GCP. Collectively, these data showed that GCP exhibits potent antitumor activity and low toxicity *in vivo*.

## Discussion

Natural products offer a rich and diverse source of traditional medicines that have been utilized since ancient times. To date, numerous plant-derived natural compounds have been reported to inhibit the growth of various cancer cells, and more than 50% of FDA-approved drugs in clinical use are of natural product origin (Lao et al., 2014). Previous studies have documented that three kinds of flavonoid isolated from *T. kirilowii* induced G2/M cell cycle arrest, apoptosis, and autophagy through the downregulation of PI3Kγ-mediated PI3K/AKT/mTOR signaling in human breast cancer cell lines (Zhang et al., 2018). Another report indicated that erianin, a natural product derived from *Dendrobium chrysotoxum* significantly induced G2/M cell cycle arrest and caused cell apoptosis and autophagy by modulating the ROS/JNK signaling pathway in human osteosarcoma cells (Wang et al., 2016). In addition, arsenic sulfide, the main active ingredient of the traditional Chinese medicine realgar, has been reported to induce G2/M cell cycle arrest, apoptosis, and autophagy *via* the activation of ROS/JNK signaling and the blockade of the AKT/mTOR pathway in human osteosarcoma cells (Wang et al., 2017).

*Descurainia sophia* (L.) Webb. ex Prantl is a medicinal herb widely used in Asian countries to treat various ailments. GCP is an important constituent of *Descurainia sophia*, and possesses several bioactivities. A natural product molecule with a unique structure, the pharmacological activity of GCP has seldom been studied. According to our preliminary study (Shi et al., 2013), GCP represents a promising drug candidate for the treatment of cervical cancer. Therefore, in the current study, we comprehensively analyzed the effect of GCP on cervical cancer using *in vitro* and *in vivo* models, and for the first time attempted to delineate the molecular mechanisms involved. The results revealed that GCP targets and reduces the activities of CDK1 and PLK1, thereby suppressing cell proliferation, inducing cell cycle arrest in G2/M phase, promoting cancer cell apoptosis *via* the intrinsic mitochondrial pathway, and inducing autophagy *via* the PI3K/AKT/mTOR signaling pathway. Moreover, in our study, inhibition of autophagy by treatment with 3-MA diminished GCP-induced apoptosis, indicating that the autophagy induced by GCP contributes to the cell survival.

The dysregulation of cell cycle regulators is one of the hallmarks of cancer, rendering them attractive therapeutic targets in cancer treatment (Hanahan and Weinberg, 2011). Numerous reports have identified that the initiation, tumorigenesis, and development of cervical cancer occur as a result of cell cycle instability (Qiao et al., 2018). Previous studies have indicated that autophagy and the cell cycle are coordinated and reciprocally regulated. However, to the best of our knowledge, the roles of autophagy regulation and function in the orchestration of the cell cycle need to be clarified. Cell cycle arrest can induce apoptosis, which itself plays a crucial role in the regulation of cancer formation and the treatment response. Besides apoptosis, autophagy also plays a vital role in determining the fate of cells. Autophagy modulation has received much attention due to its potential for improving anti-cancer therapeutics (Levy et al., 2017). Autophagic mediators, including ATGs, PI3K, mTOR, and Beclin-1, can integrate into cancer cell signaling networks and ultimately determine cell survival or death. Therefore, the modulation of autophagy may ultimately lead to the development of new therapeutic strategies in the fight against cancer.

CDK1 is a key cell cycle regulator and its inhibition in the eradication of cancer has been extensively studied (Gao et al., 2018). CDK1 can directly phosphorylate VPS34 at Thr159 in their common substrate recognition motif, thus interrupting the interaction between VPS34 and Beclin-1, and thereby decreasing the autophagy of mitotic cells (Funderburk et al., 2010). PLK1 and CDK1/cyclin B1 co-regulate nuclear envelope breakdown, centrosome separation, spindle assembly, chromosome condensation, and Golgi fragmentation, respectively. PLK1 plays an important role in the recovery from DNA damage-induced G2/M arrest *via* the activation of CDK1. Moreover, PLK1 is also a major cell cycle regulator and plays an important role throughout mitosis. PLK1 overexpression occurs in various cancers and is often associated with a poor prognosis (de Carcer et al., 2018). Inhibition of PLK1, thereby interfering with multiple stages of mitosis, has been adopted as an anti-cancer strategy (Dominguez-Brauer et al., 2015). To confirm whether GCP has an inhibitory effect on PLK1 and CDK1, we measured the expression of these proteins using the CDK1 kinase assay kit, the fluorescence polarization assay, RT-qPCR, and western blotting. Our results demonstrate that GCP could be targeted to attenuate the expression of CDK1 and PLK1 in a dose- and time-dependent manner.

It is well established that G2 checkpoint prevents cells from entering mitosis when DNA is damaged, providing a chance for repair and stopping the proliferation of damaged cells. The cyclin B1/CDK1 complex plays a significant role in promoting G2/M phase transition, and is controlled by a series of proteins, including CHK2, *p*-cdc25c, and p21 (Zheng et al., 2019). Numerous cytotoxic agents have the potential to induce G2/M phase arrest by targeting the cyclin B1/CDK1 complex. Moreover, it has been reported that p21 plays a critical role in blocking CDK1/cyclin B1 activation in a p53-dependent or independent manner. The converging results obtained by flow cytometry allowed us to propose that GCP may exhibit antitumor effects by inducing G2/M cell cycle arrest. In addition, our western blotting data showed that GCP can decrease the expression of Cyclin B1, CDK1, and up-regulate the levels of p21, p27, and phospho-Cdc25, which caused the accumulation of cells in G2/M phase.

Mounting evidence suggests that autophagy has an inseparable relationship with apoptosis and that both processes can be co-regulated in cancer cells (Ferro et al., 2020). While the inhibition of autophagy can enhance apoptosis in cancer cells (Singh et al., 2018), in some situations, autophagy promotes the pro-apoptotic effect (Dang et al., 2015). In the present study, we showed that GCP can significantly induce apoptosis in HeLa cells by decreasing mitochondrial membrane potential and increasing the levels of cleaved caspases 3 and 9, and PARP in a dose-dependent manner. Furthermore, GCP also induced autophagy in HeLa cells in a dose-dependent manner, as shown by AO and MDC staining, and western blotting. However, the autophagy inhibitor 3-MA suppressed GCP-mediated apoptosis, suggesting that autophagic pathway activation in GCP-treated cells leads to autophagic cell death. Therefore, in the case of extensive cell damage, another programmed cell death pathway (autophagic cell death) is promoted by autophagy.

Treatment with GCP induced a significant decrease in PI3K, AKT, and mTOR phosphorylation in a dose-dependent manner. Previous studies have identified that several signaling molecules, including mTOR, MAPKs, and class III PI3K, have been reported to have an intricate relationship with both apoptosis and autophagy (Xu et al., 2020). The AKT and mTOR major regulatory signaling pathways are involved in the modulation of the proliferation, metabolism, and survival of cancer cells. Beclin one enhances autophagy by combining with PI3KIII in the initiating stage of autophagy (Liang et al., 2019). mTOR1, as a central regulator of cell growth and proliferation, has been considered as an attractive target for cancer therapy (Ferro et al., 2020). The PI3K/AKT/mTOR1 pathway promotes cancer cell growth but inhibits autophagy. Mounting evidence has indicated that numerous anti-tumor drugs can promote apoptosis and autophagy through the inhibition of AKT/mTOR signaling (Hua et al., 2019).

## Conclusions

**FIGURE 9:**
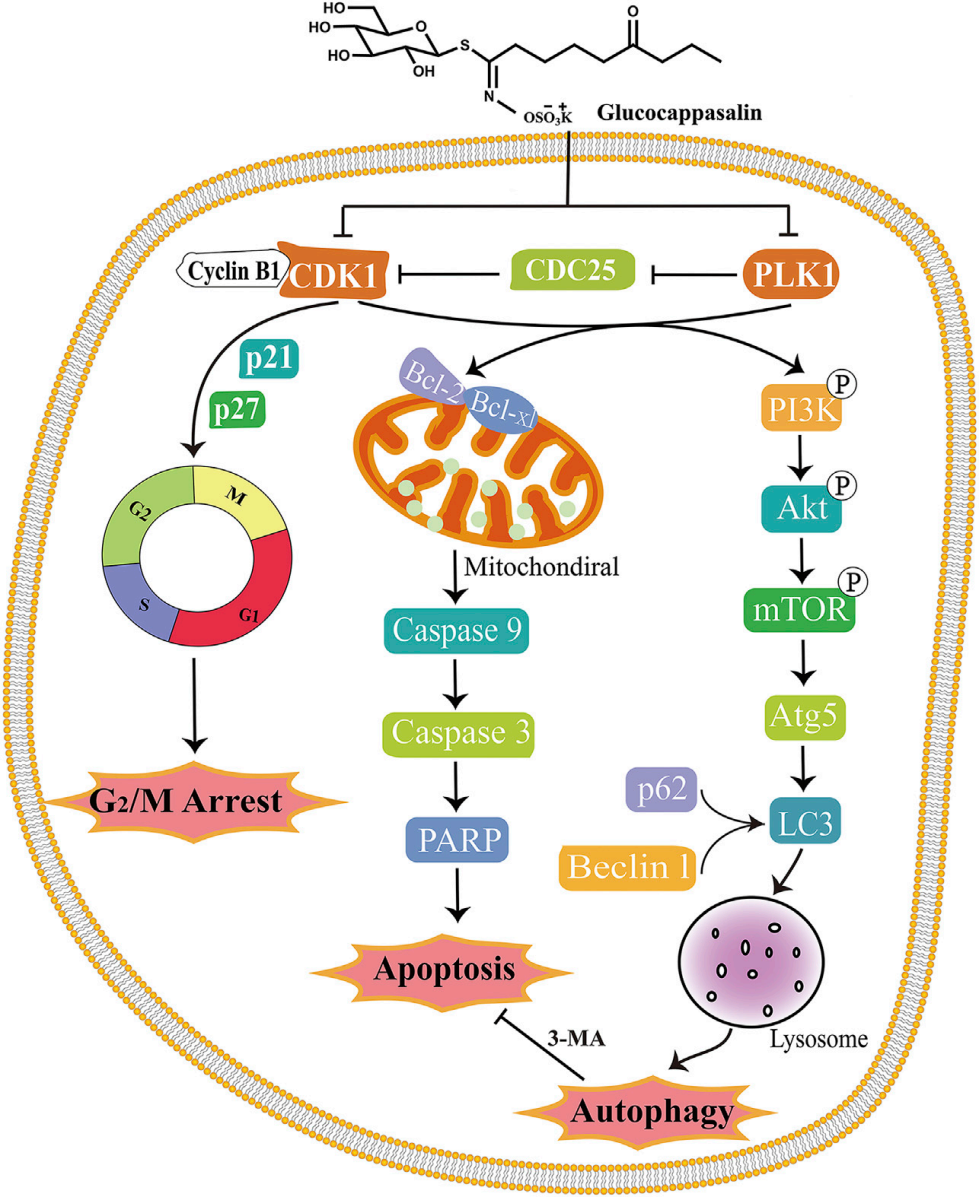
Schematic model of the molecular mechanisms illustrating how GCP induces G2/M arrest, apoptosis and autophagy in HeLa cells. After treatment, GCP suppresses the CDK1 and PLK1, leading to cell cycle arrest at G2/M phase, mitochondrial apoptosis, and autophagy *via* PI3K/Akt/mTOR signaling pathway.

In summary, we have demonstrated for the first time that GCP treatment contributes to cell death by targeting CDK1 and PLK1 and mediating G2/M cell cycle arrest *via* both autophagy and apoptosis. In addition, we demonstrated that GCP significantly decreases tumor growth in mice bearing xenografts. Our findings indicate that GCP could be considered a novel drug candidate, targeting both apoptotic and autophagic cell death pathways, in the treatment of cervical cancer. Our findings may not only pave the road for understanding the molecular mechanisms underpinning the antitumor activities of GCP, but also suggest that GCP may be used as an alternative therapeutic agent and a candidate for drug repositioning in cervical cancer. A schematic model outlining the molecular mechanisms associated with the GCP-mediated treatment of cervical carcinoma is shown in Figure 9.

## Data Availability

The original contributions presented in the study are included in the article/Supplementary Material, further inquiries can be directed to the corresponding authors.
