# Supplementary figures and images for: Glucocappasalin Induces G2/M-Phase Arrest, Apoptosis, and Autophagy Pathways by Targeting CDK1 and PLK1 in Cervical Carcinoma Cells
Source: Front Pharmacol. 2021 May 20;12:671138. doi: 10.3389/fphar.2021.671138 (PMC8172611; doi:10.3389/fphar.2021.671138)

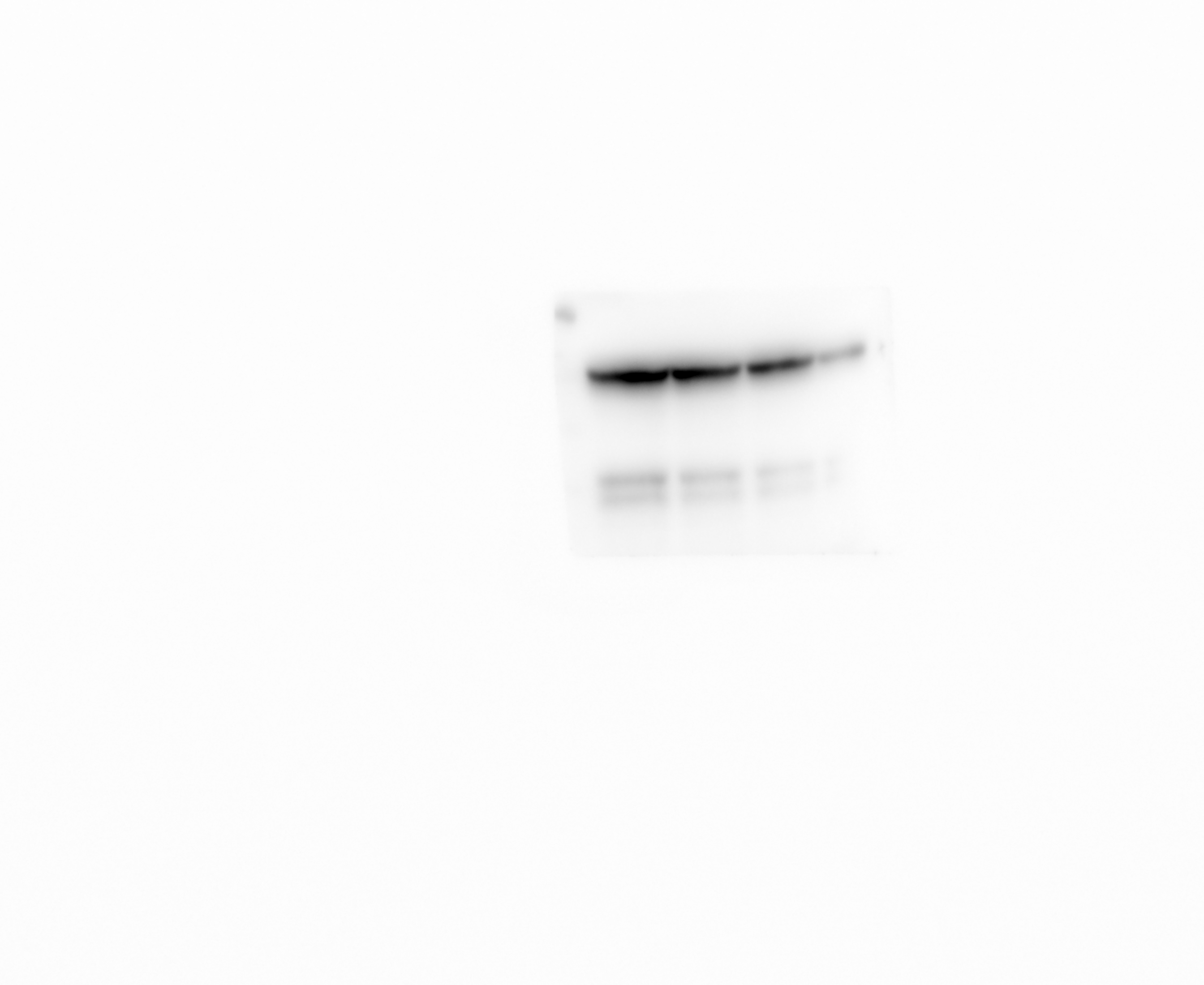

Supplement: Supplementary file 2 [file DataSheet3.ZIP › WB-fig 3/CDK1/20201223_144829_0 .5.0_4.tif]

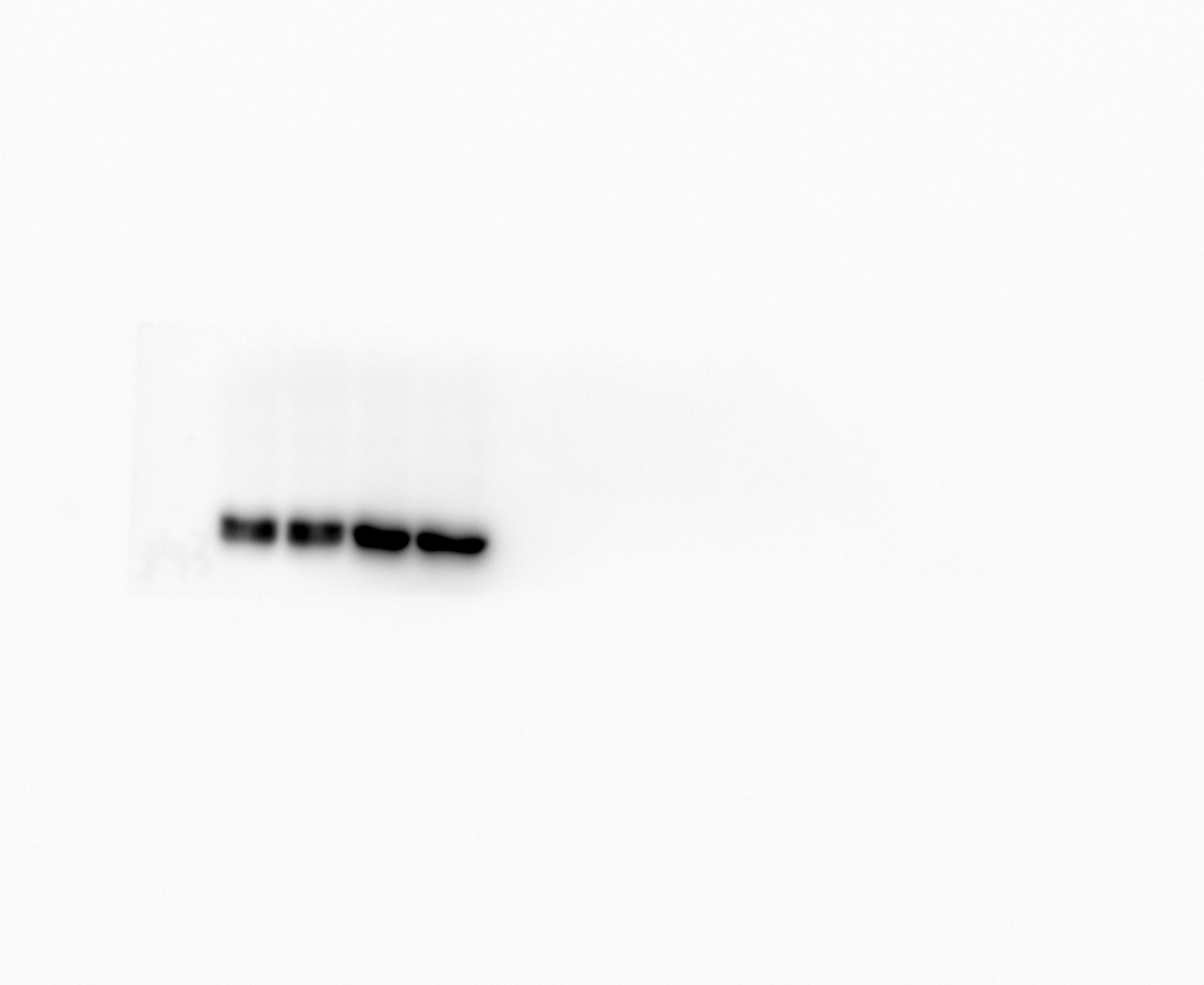

Supplement: Supplementary file 2 [file DataSheet3.ZIP › WB-fig 3/GAPDH/20200624_09 5133_0.10.0_1.tif]

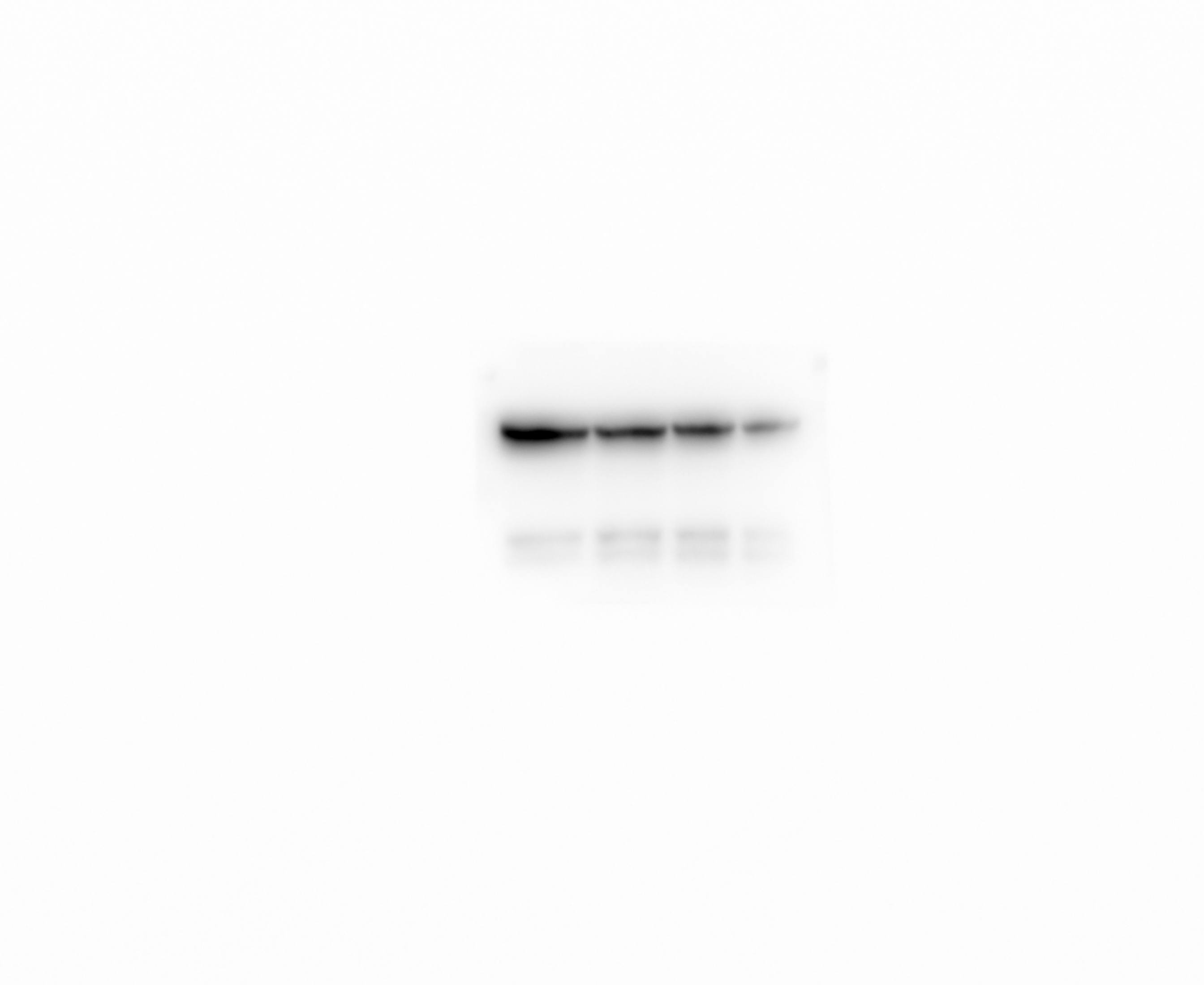

Supplement: Supplementary file 2 [file DataSheet3.ZIP › WB-fig 3/PLK1/202012 23_144606_0.5.0_3.tif]

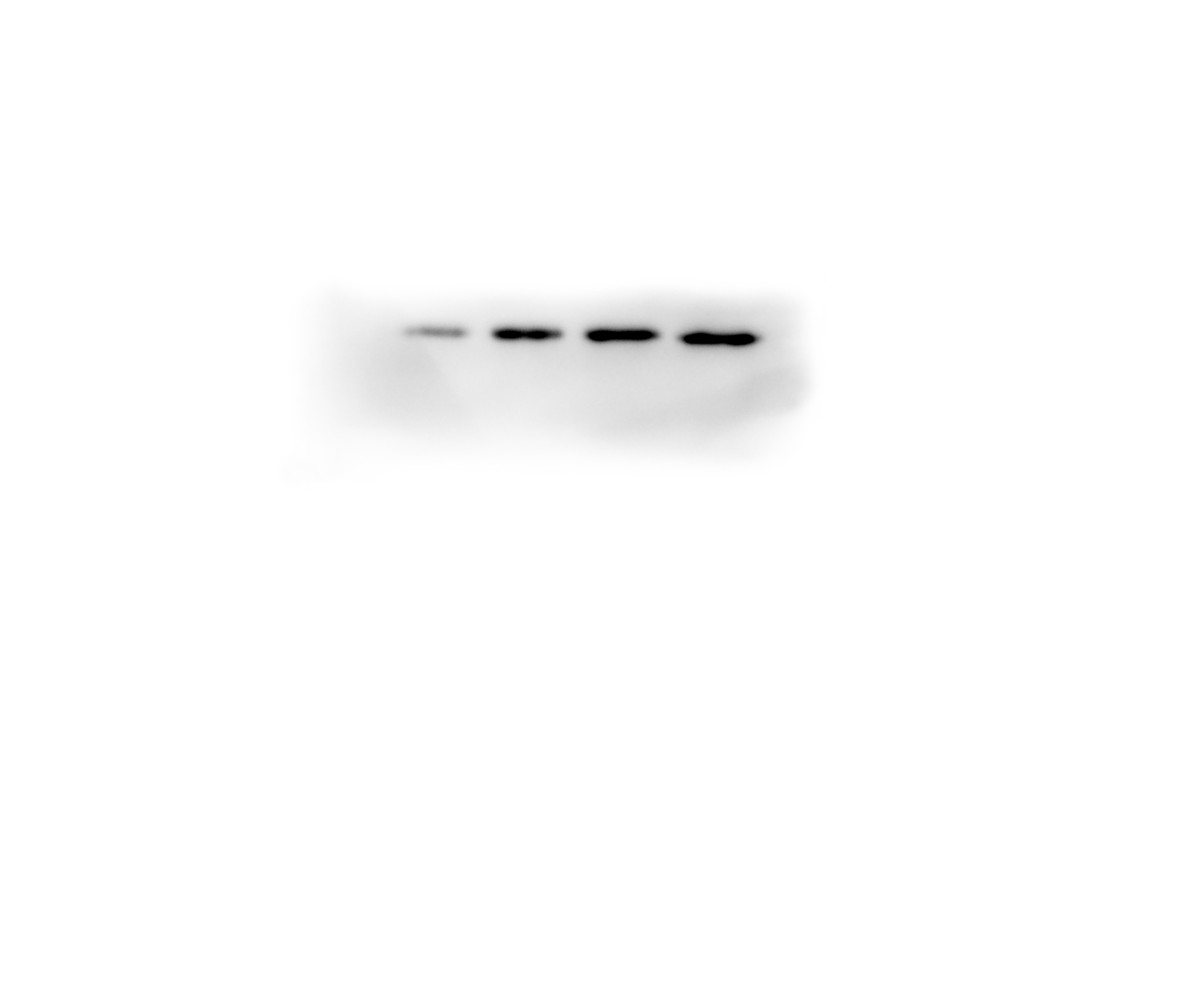

Supplement: Supplementary file 4 [file DataSheet4.ZIP › WB-fig 4/cdk1/20190803_1636 13_0.5.0_2.tif]

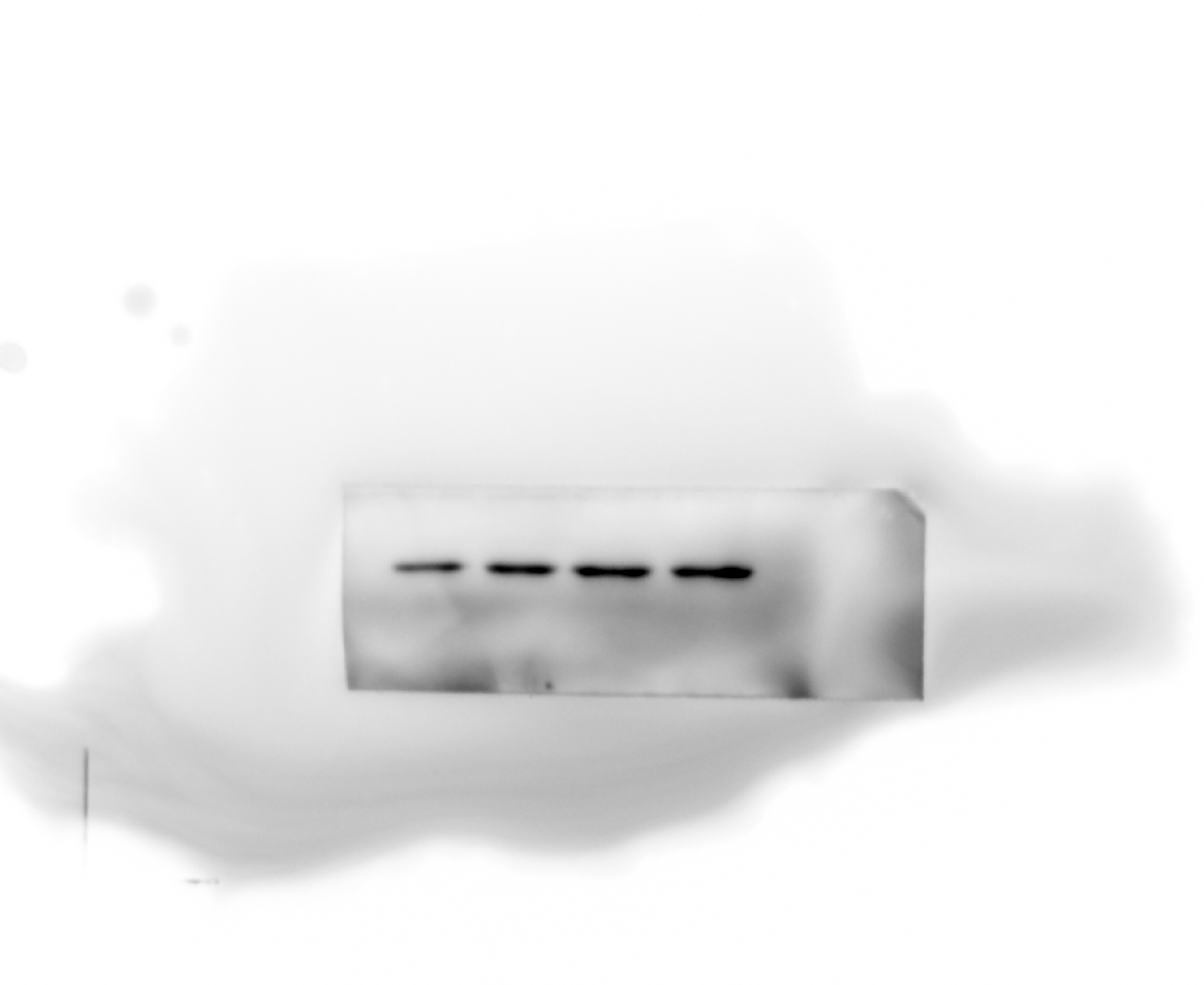

Supplement: Supplementary file 4 [file DataSheet4.ZIP › WB-fig 4/cyclin b1/20190803_165758_0.15.0_ 3.tif]

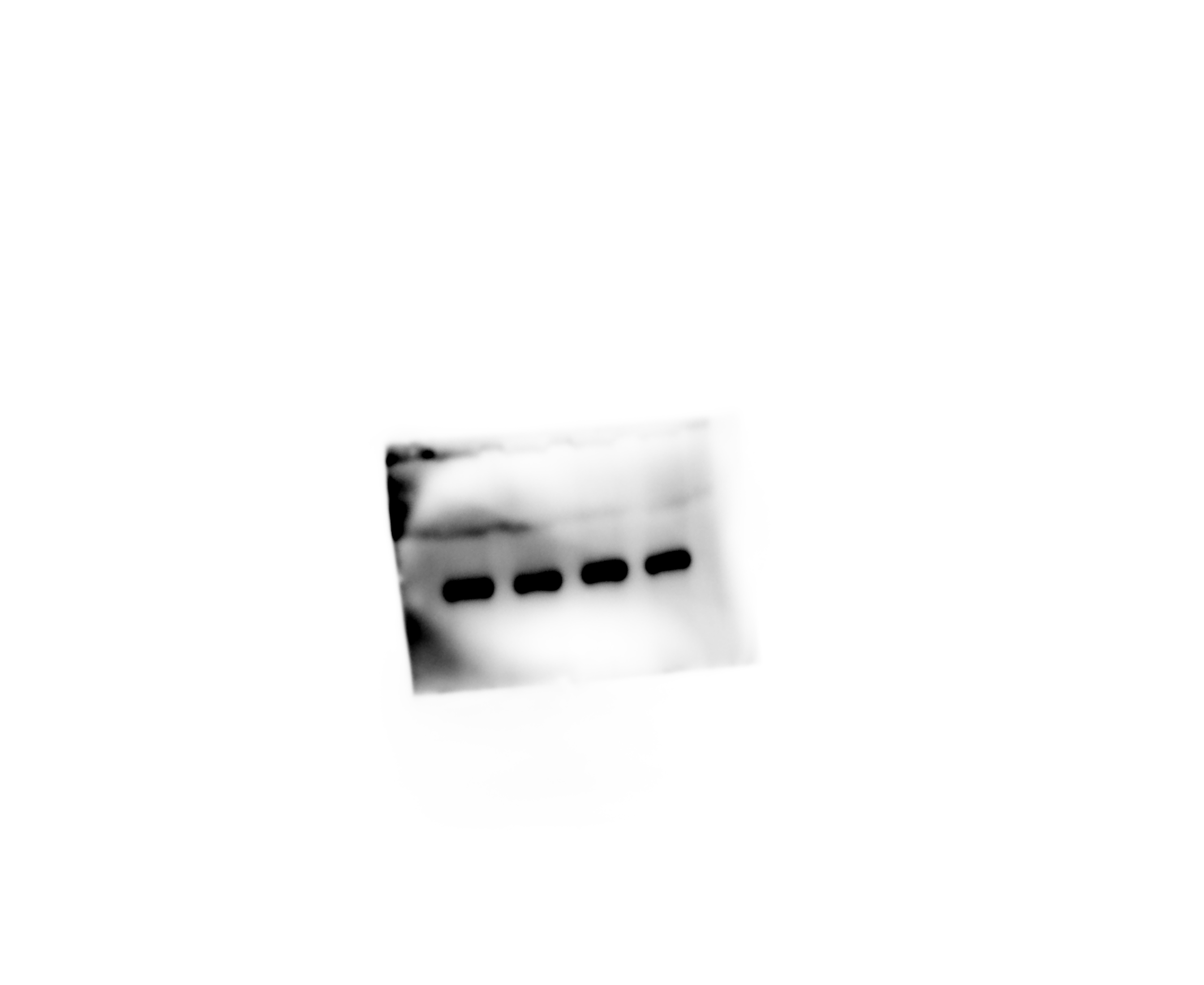

Supplement: Supplementary file 4 [file DataSheet4.ZIP › WB-fig 4/GAPDH/20190803_173318_0.10.0_ 2.tif]

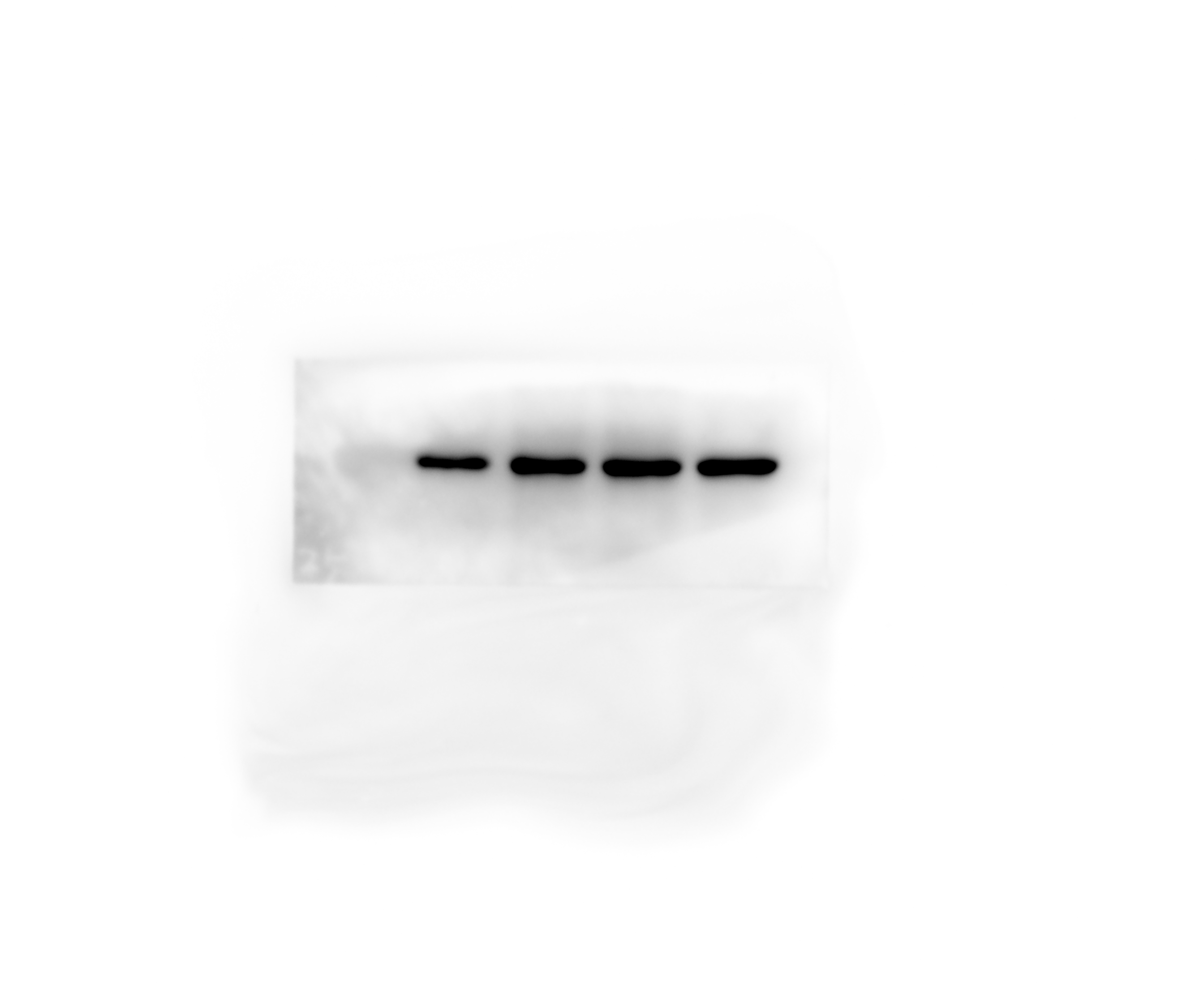

Supplement: Supplementary file 4 [file DataSheet4.ZIP › WB-fig 4/p-cdc25/20190803_164641_0. 8.0_3.tif]

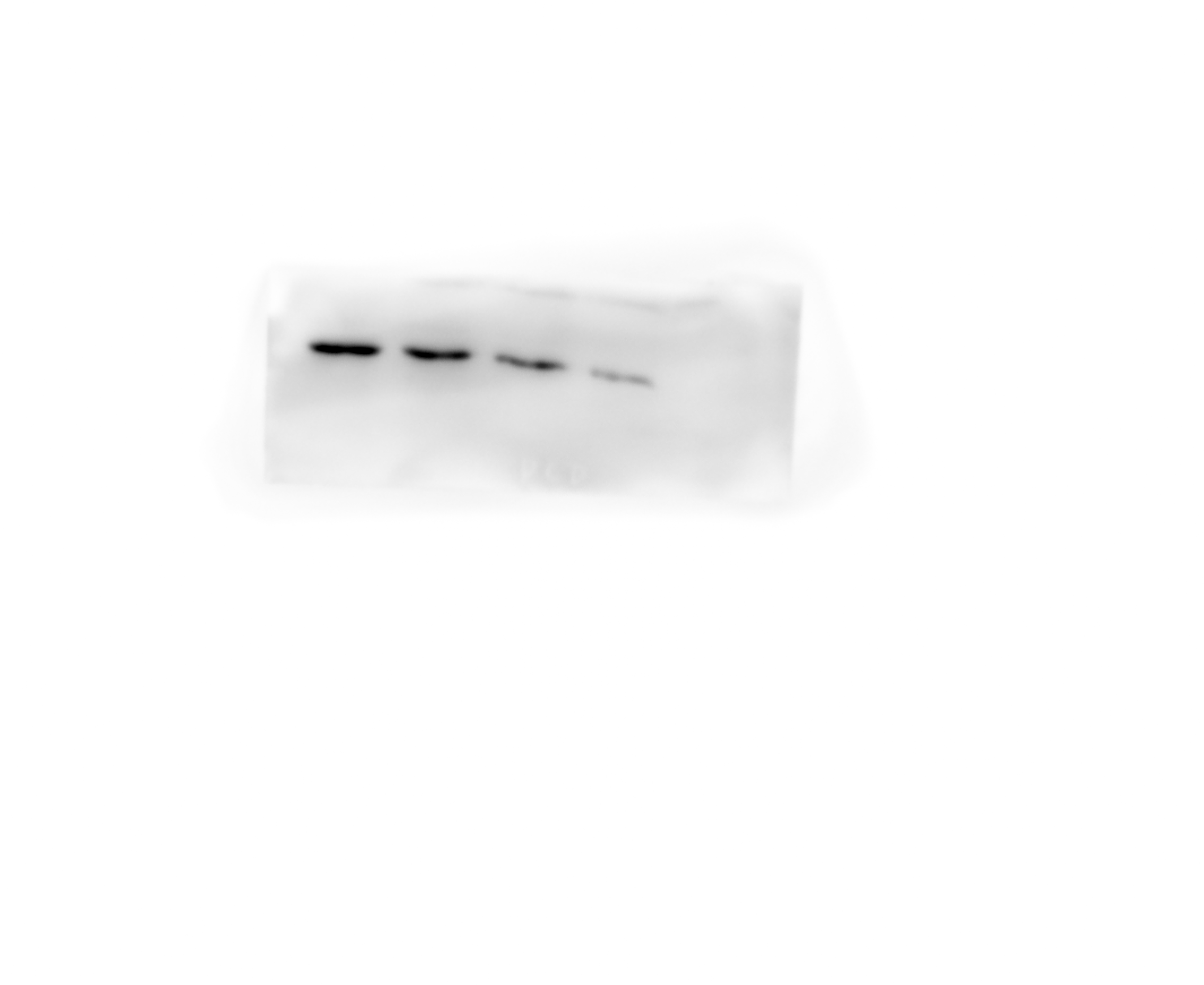

Supplement: Supplementary file 4 [file DataSheet4.ZIP › WB-fig 4/p-cdk1/20190803_164005 _0.8.0_1.tif]

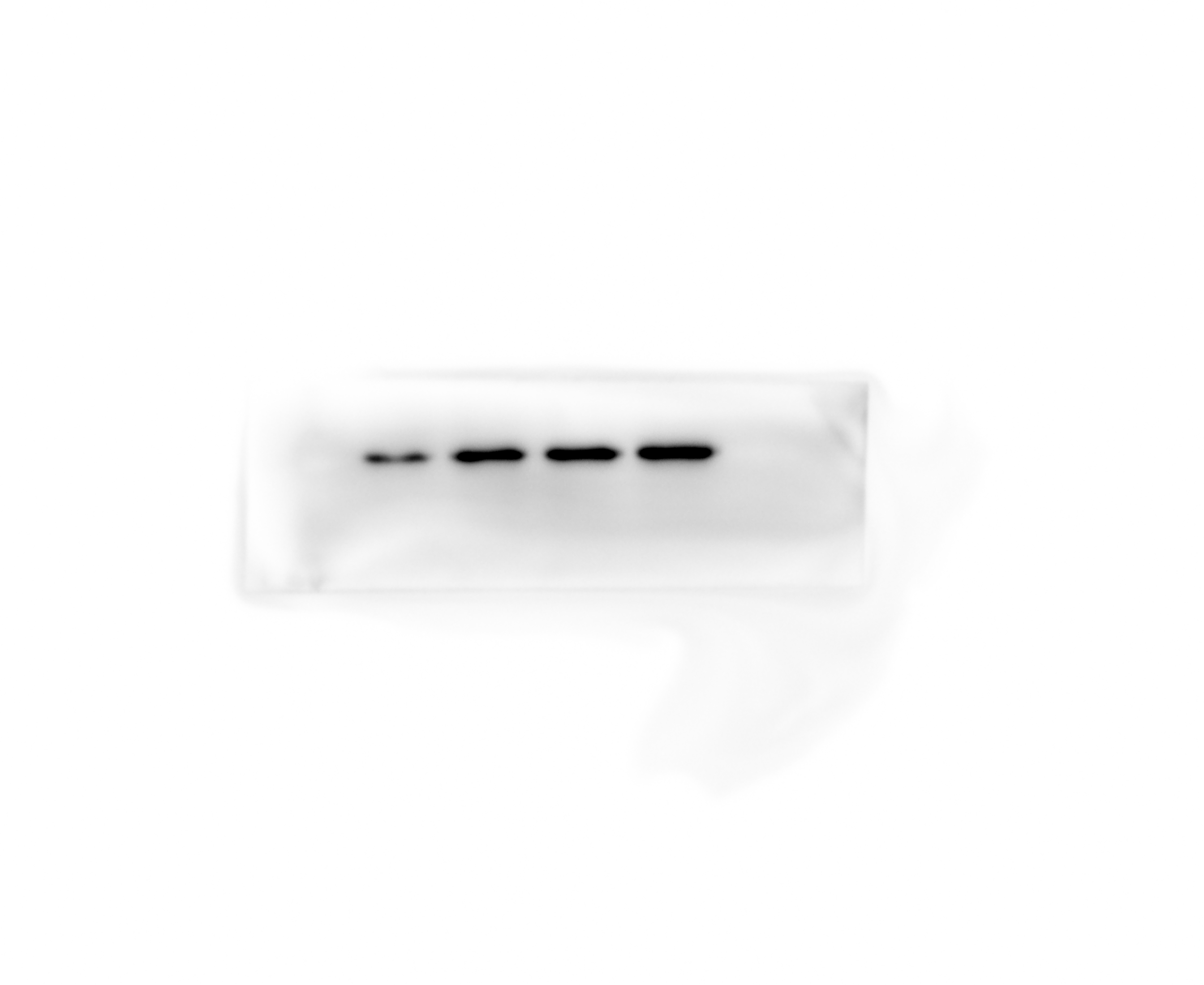

Supplement: Supplementary file 4 [file DataSheet4.ZIP › WB-fig 4/p21/20190803_155923_0.10 0_1.tif]

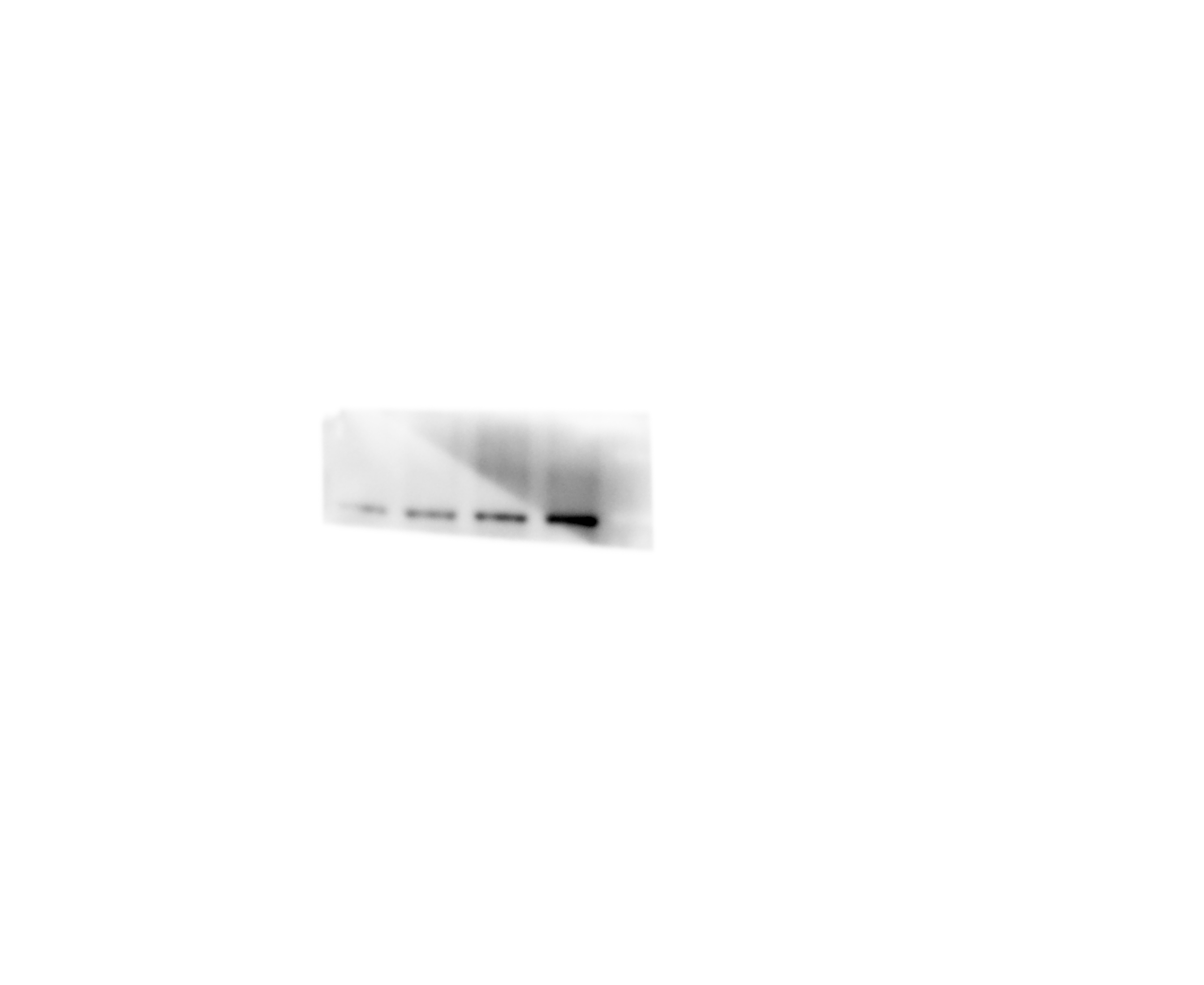

Supplement: Supplementary file 4 [file DataSheet4.ZIP › WB-fig 4/p27/20190709_192403_0.5. dd0_1.tif]

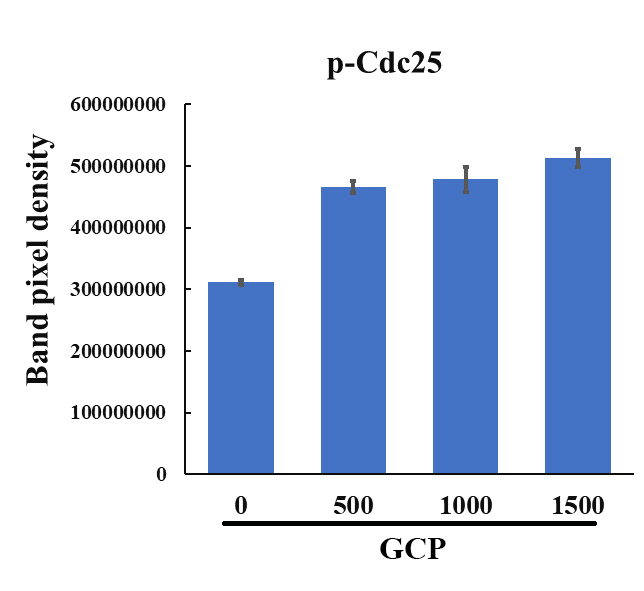

Supplement: Supplementary file 5 [file Image1.JPEG]

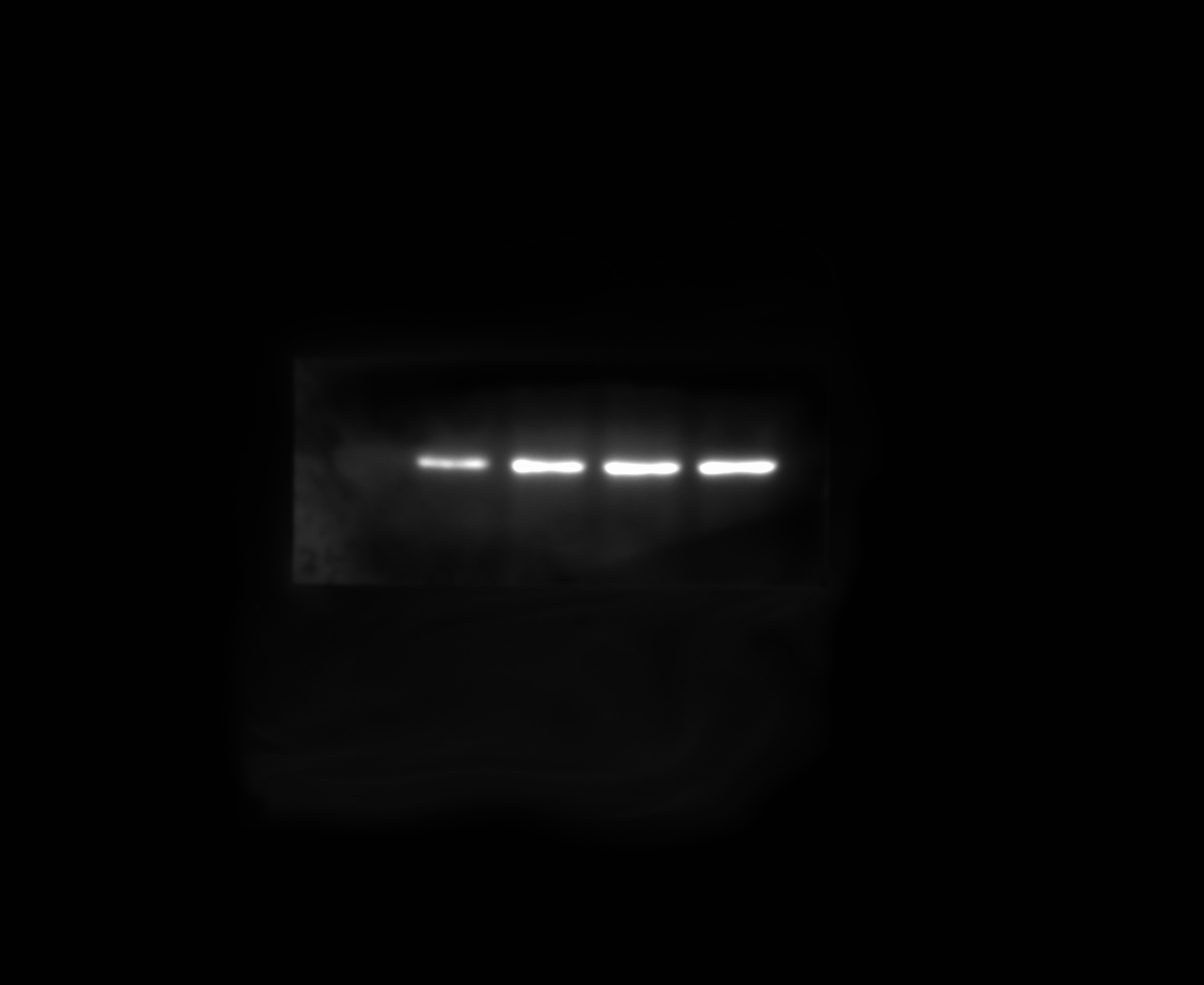

Supplement: Supplementary file 6 [file Image2.TIF]

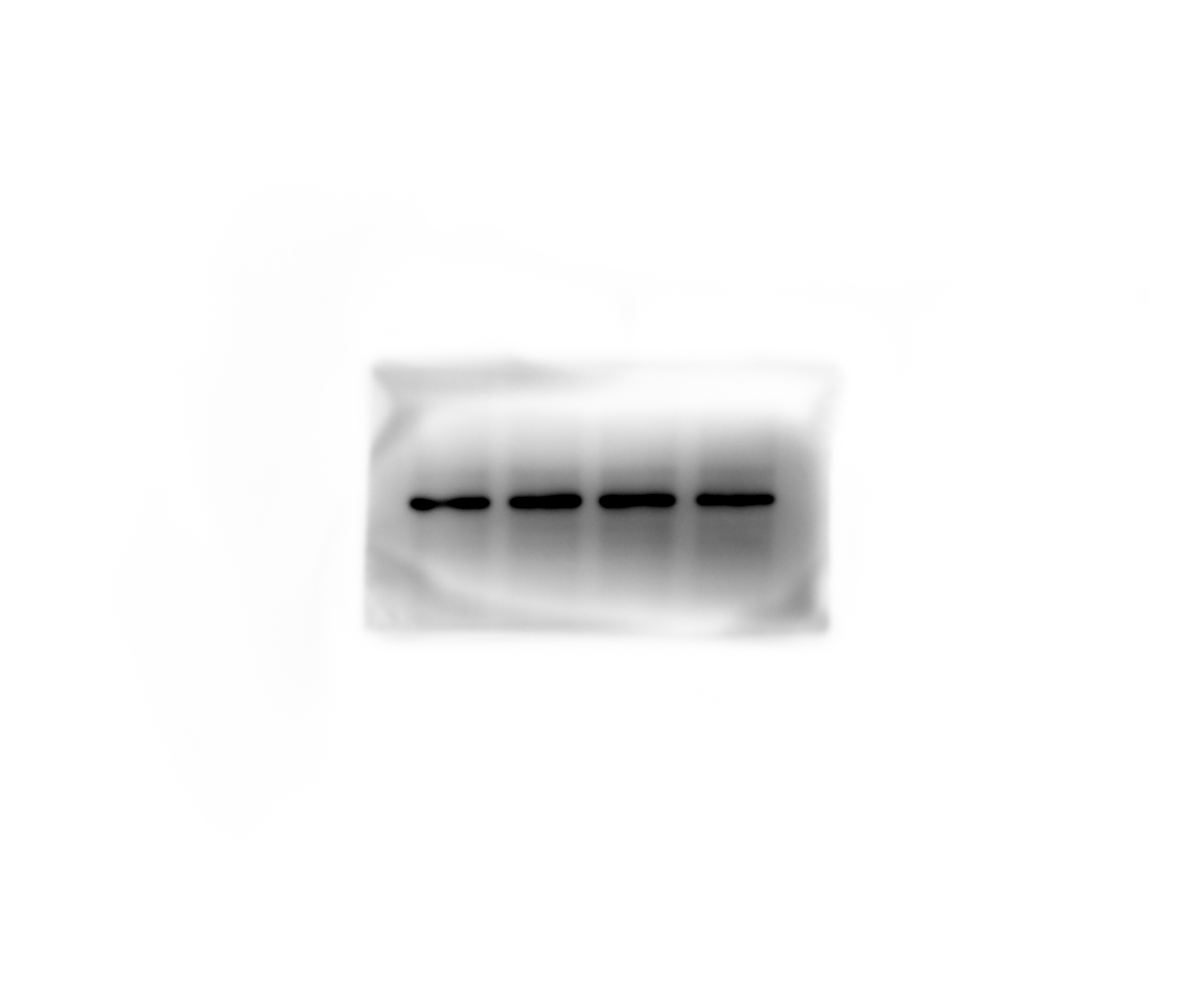

Supplement: Supplementary file 7 [file DataSheet6.ZIP › WB-fig 6/akt/20190731_195 942_0.15.0_3.tif]

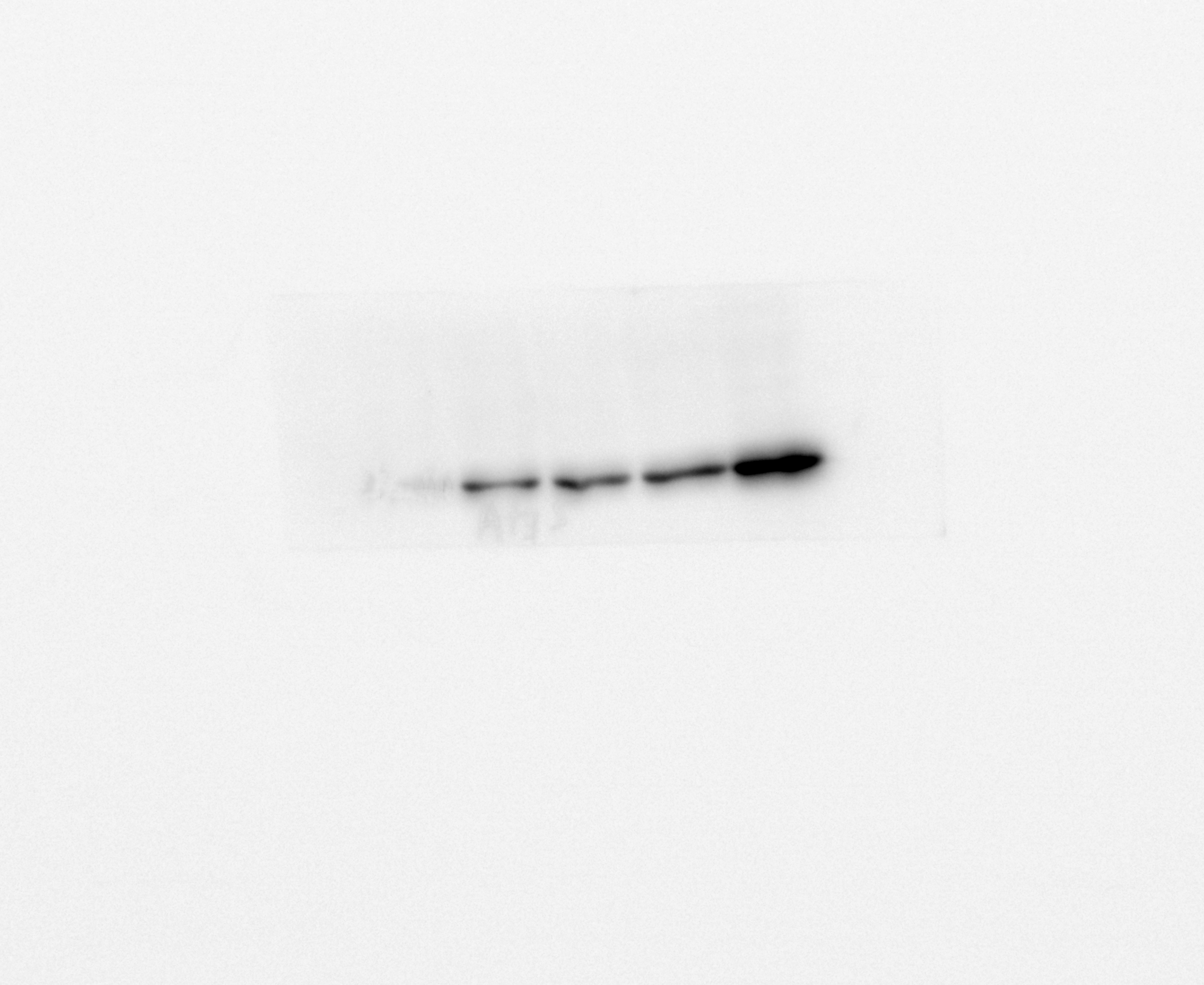

Supplement: Supplementary file 7 [file DataSheet6.ZIP › WB-fig 6/Atg5/2020062 4_103509_0. 。5.0_4.tif]

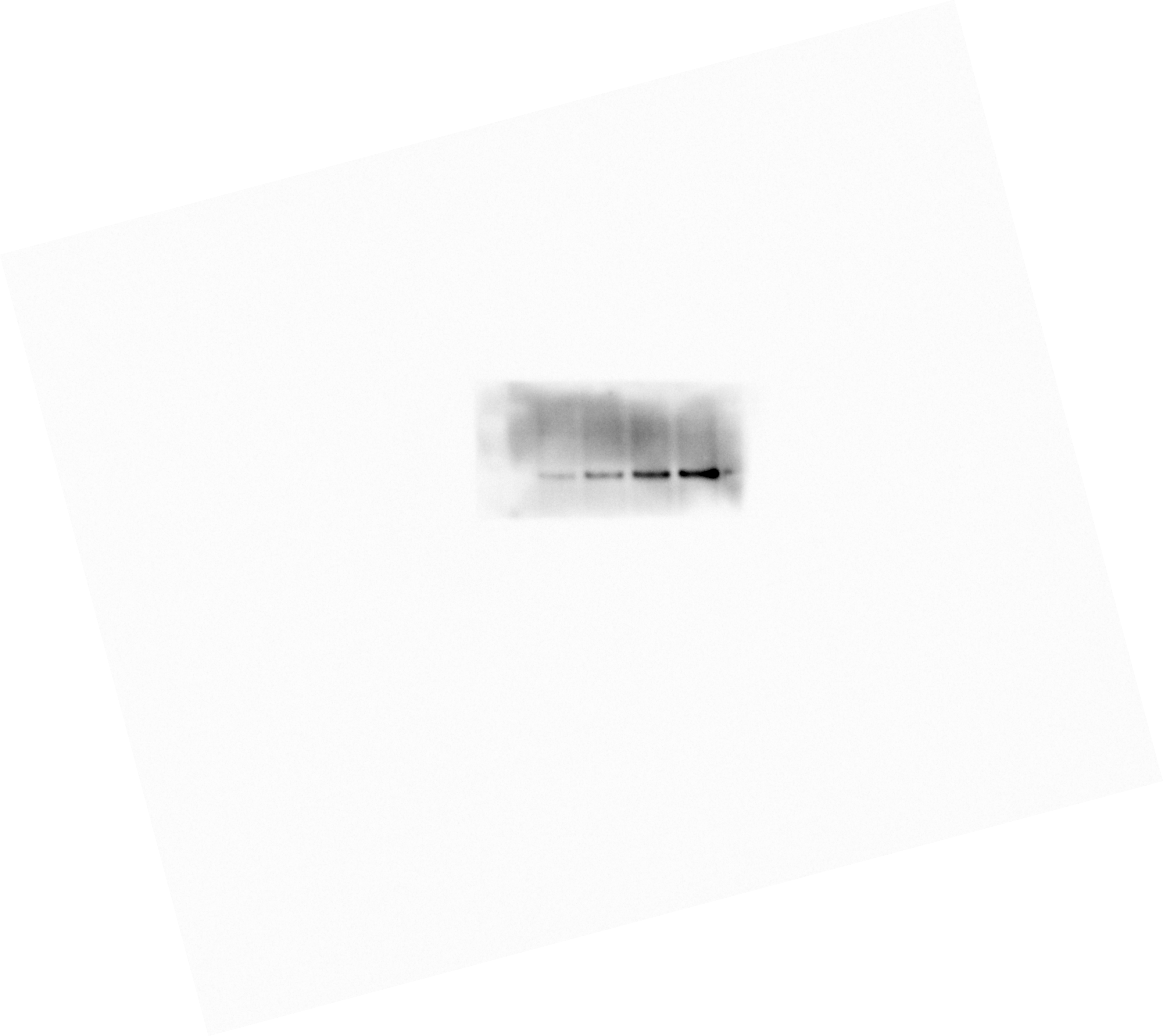

Supplement: Supplementary file 7 [file DataSheet6.ZIP › WB-fig 6/Beclin/20190708_211954_0.2.1_3.bmp]

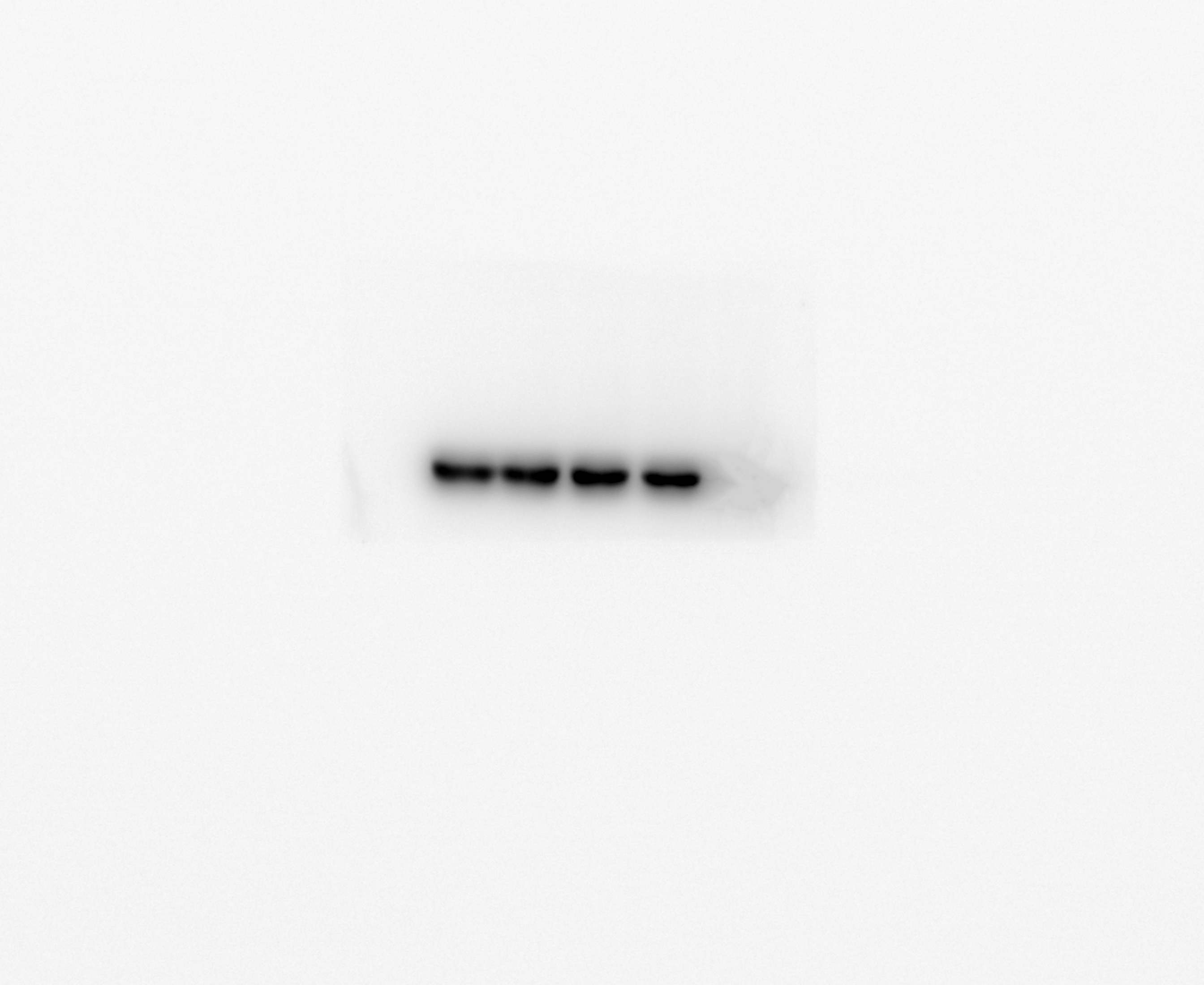

Supplement: Supplementary file 7 [file DataSheet6.ZIP › WB-fig 6/GAPDH1/202006 24_113909_0.5.0_4.tif]

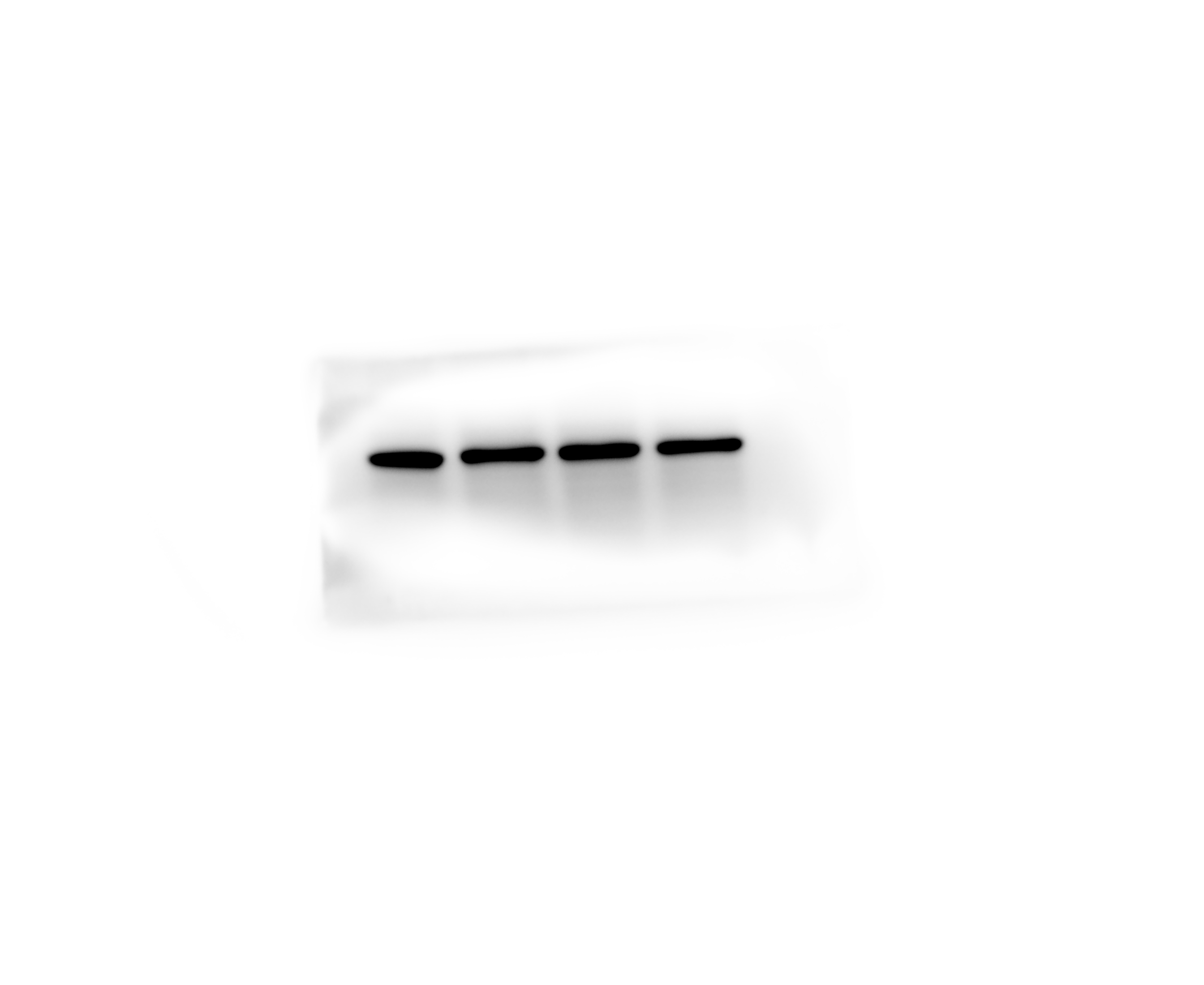

Supplement: Supplementary file 7 [file DataSheet6.ZIP › WB-fig 6/GAPDH2/20190731_193158_0.15.0 _3.tif]

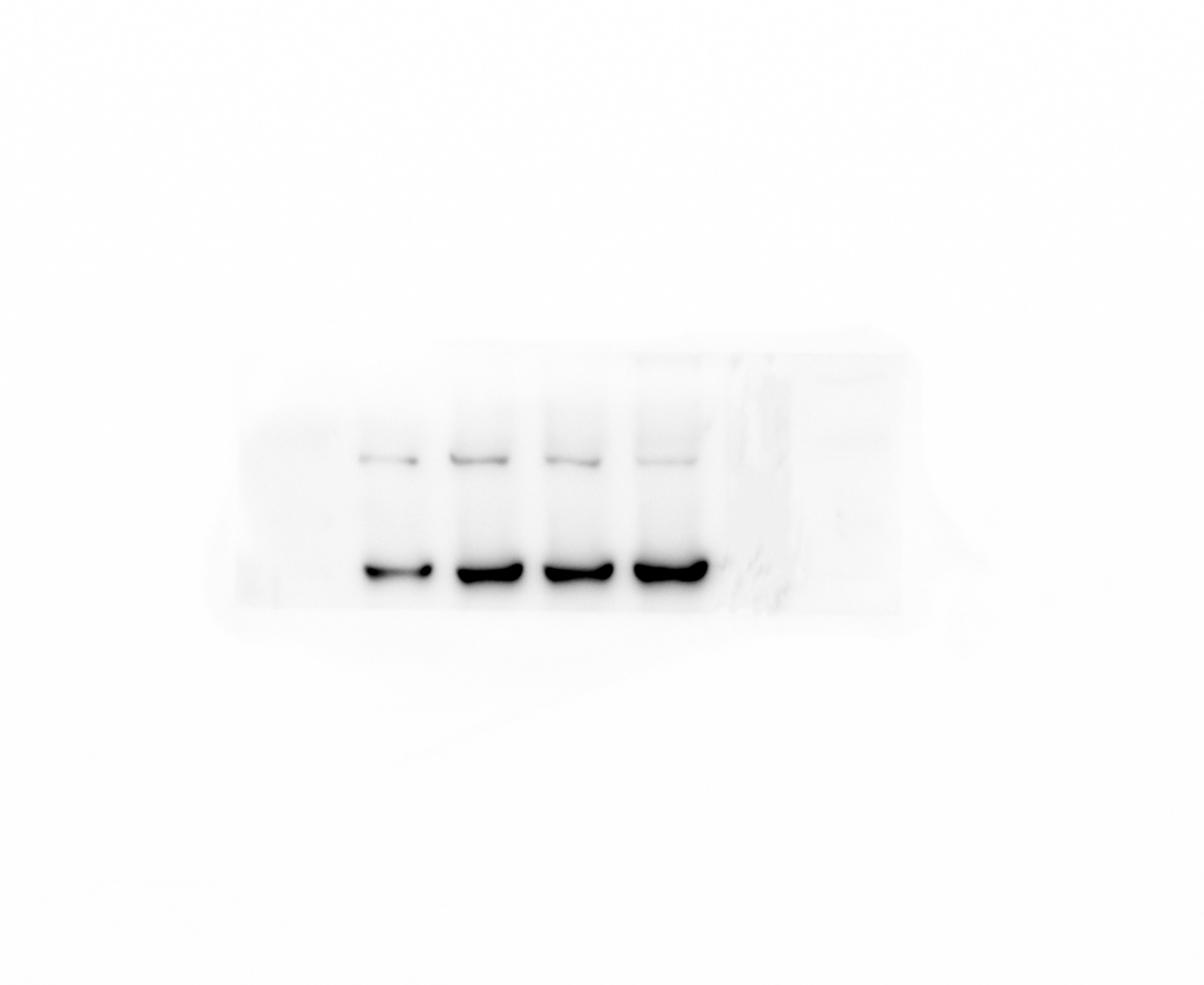

Supplement: Supplementary file 7 [file DataSheet6.ZIP › WB-fig 6/LC3B/20200630_1410 49_0.30.0_4.tif]

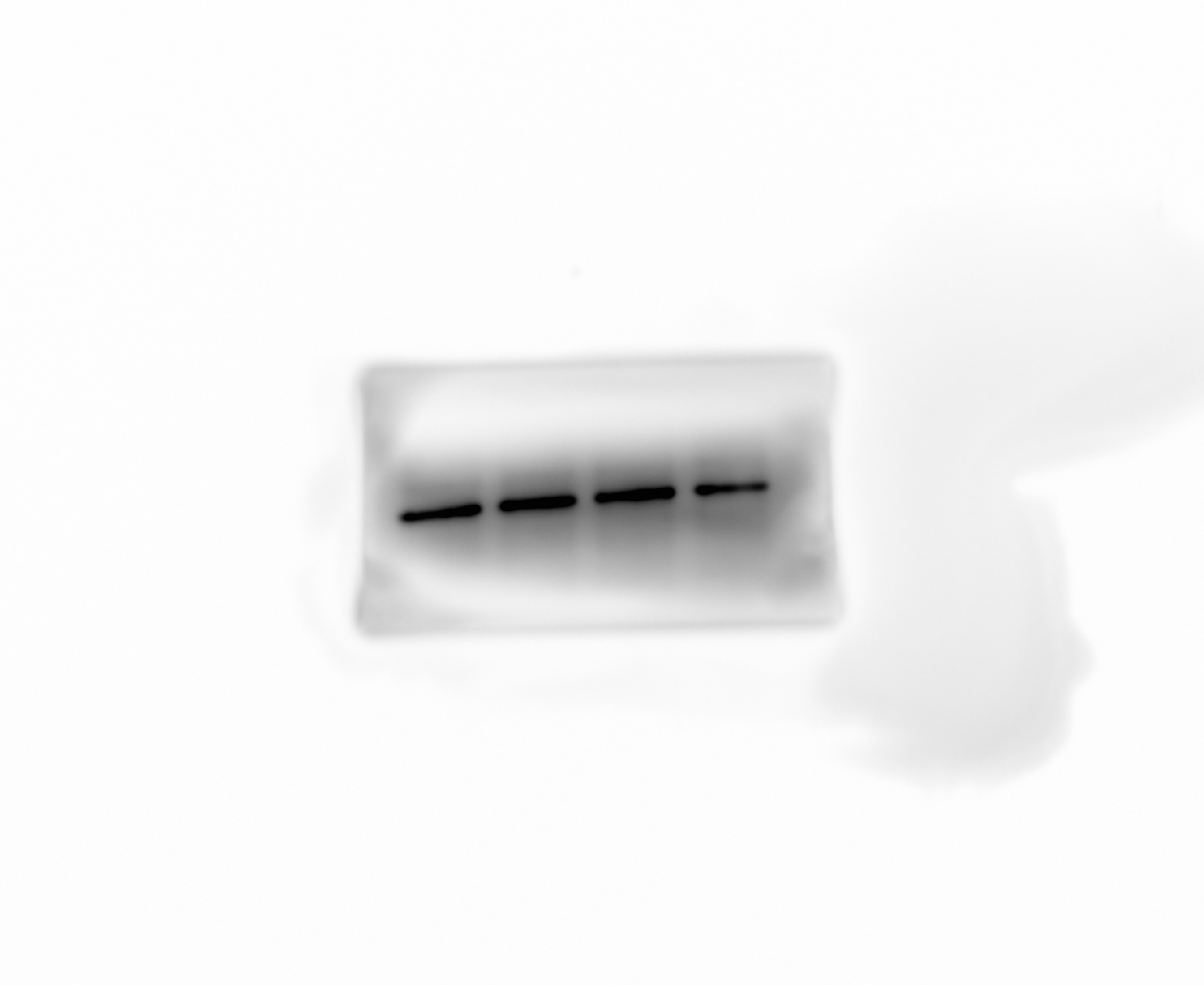

Supplement: Supplementary file 7 [file DataSheet6.ZIP › WB-fig 6/mtor/20190731_194853 _0.25.0_2.tif]

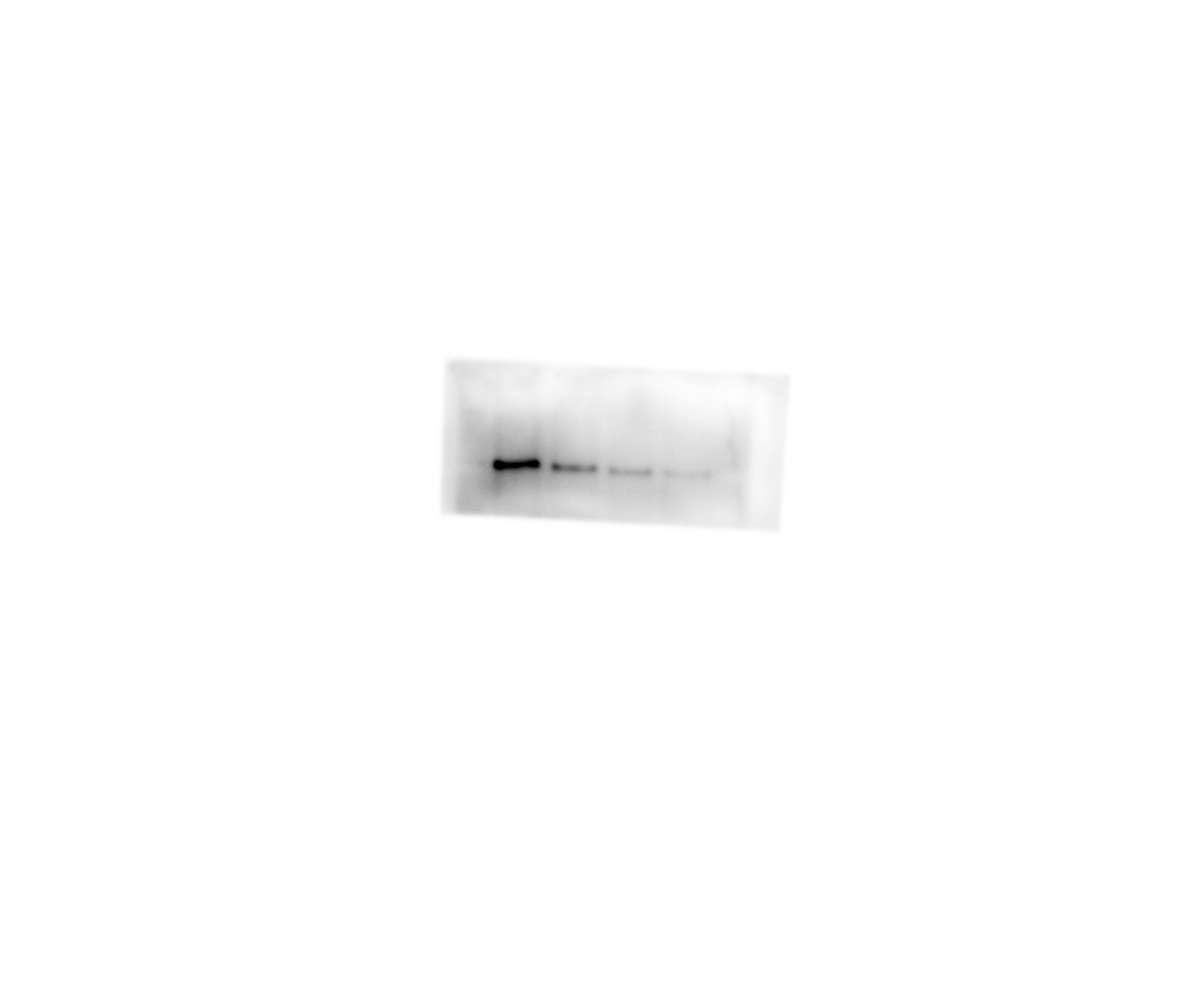

Supplement: Supplementary file 7 [file DataSheet6.ZIP › WB-fig 6/p-akt/20190708_212416_0.4.1_ 5.bmp]

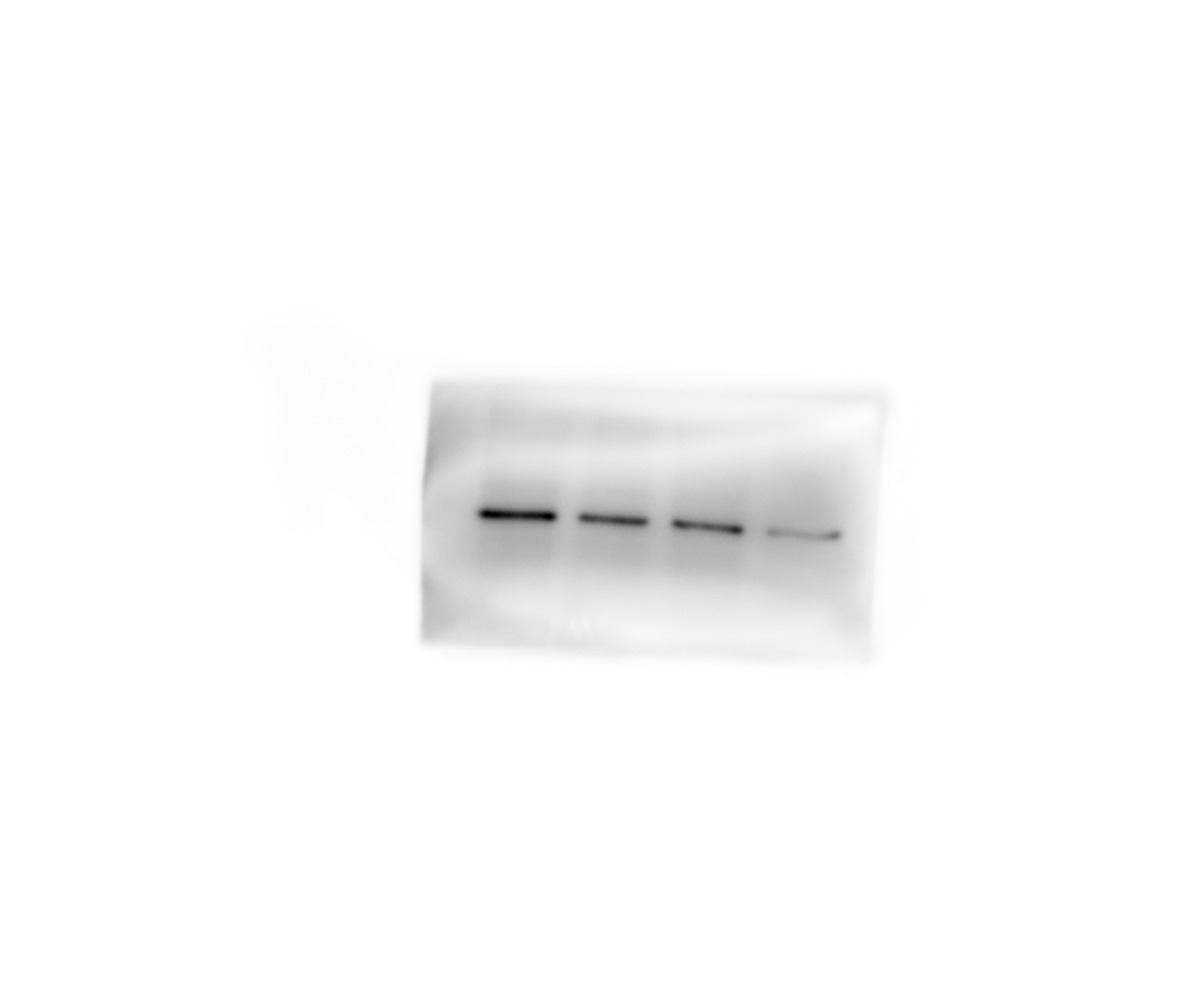

Supplement: Supplementary file 7 [file DataSheet6.ZIP › WB-fig 6/p-mtor/20190731_200507 _0.20.0_1.tif]

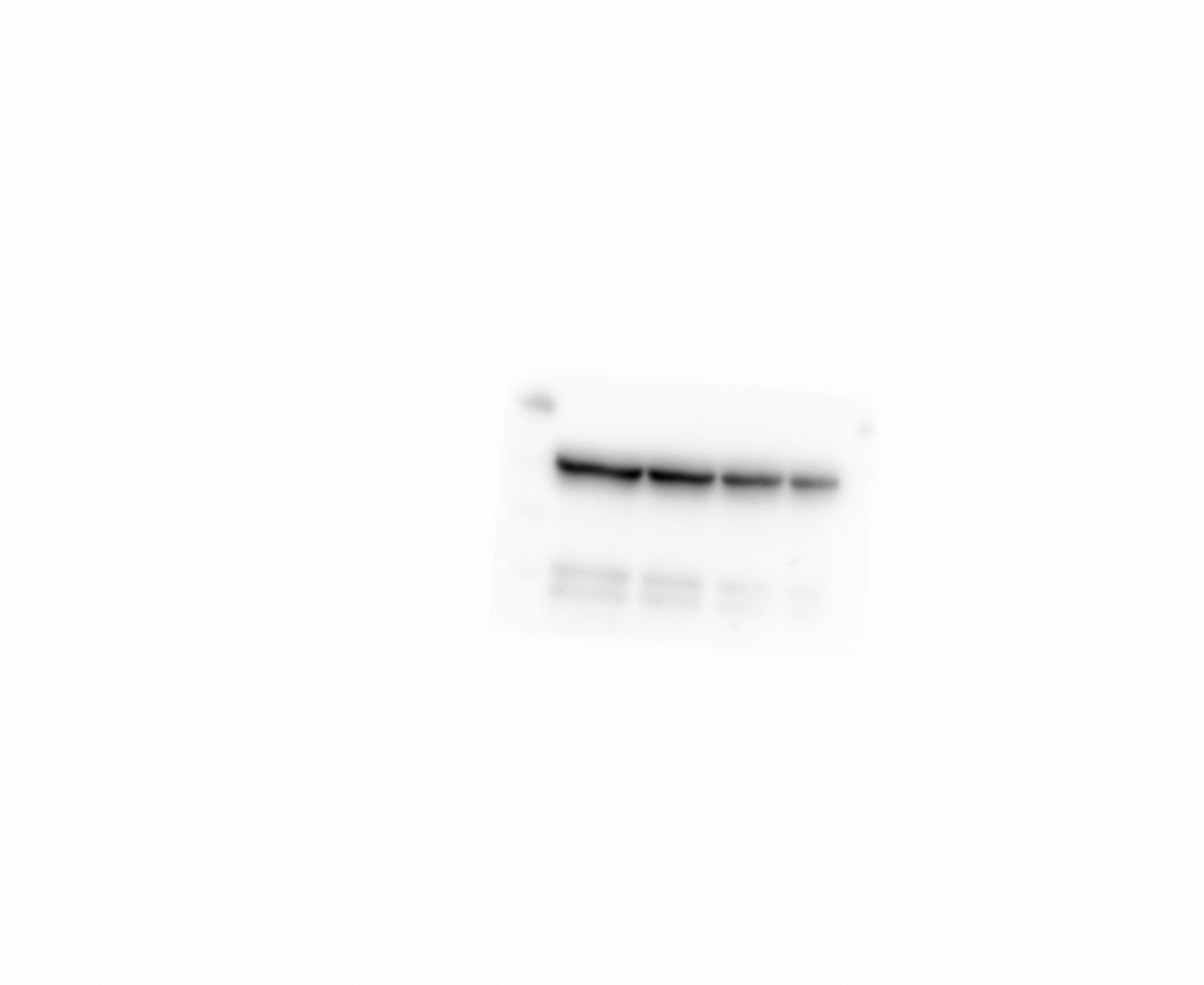

Supplement: Supplementary file 7 [file DataSheet6.ZIP › WB-fig 6/P-PI3K/2020 1223_144335_0.5.0_1.tif]

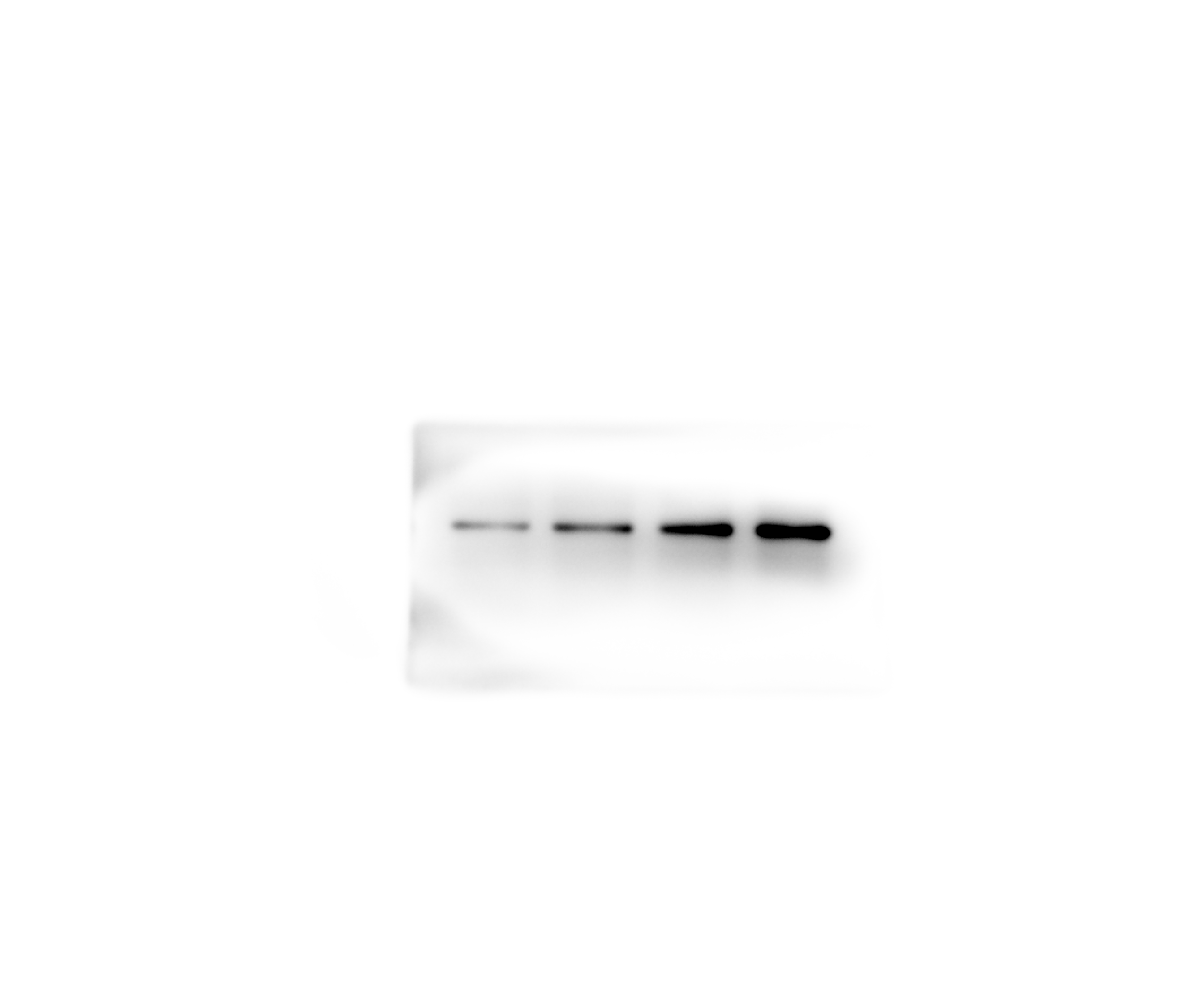

Supplement: Supplementary file 7 [file DataSheet6.ZIP › WB-fig 6/p62/20190731_19242 5_0.7.0_4.tif]

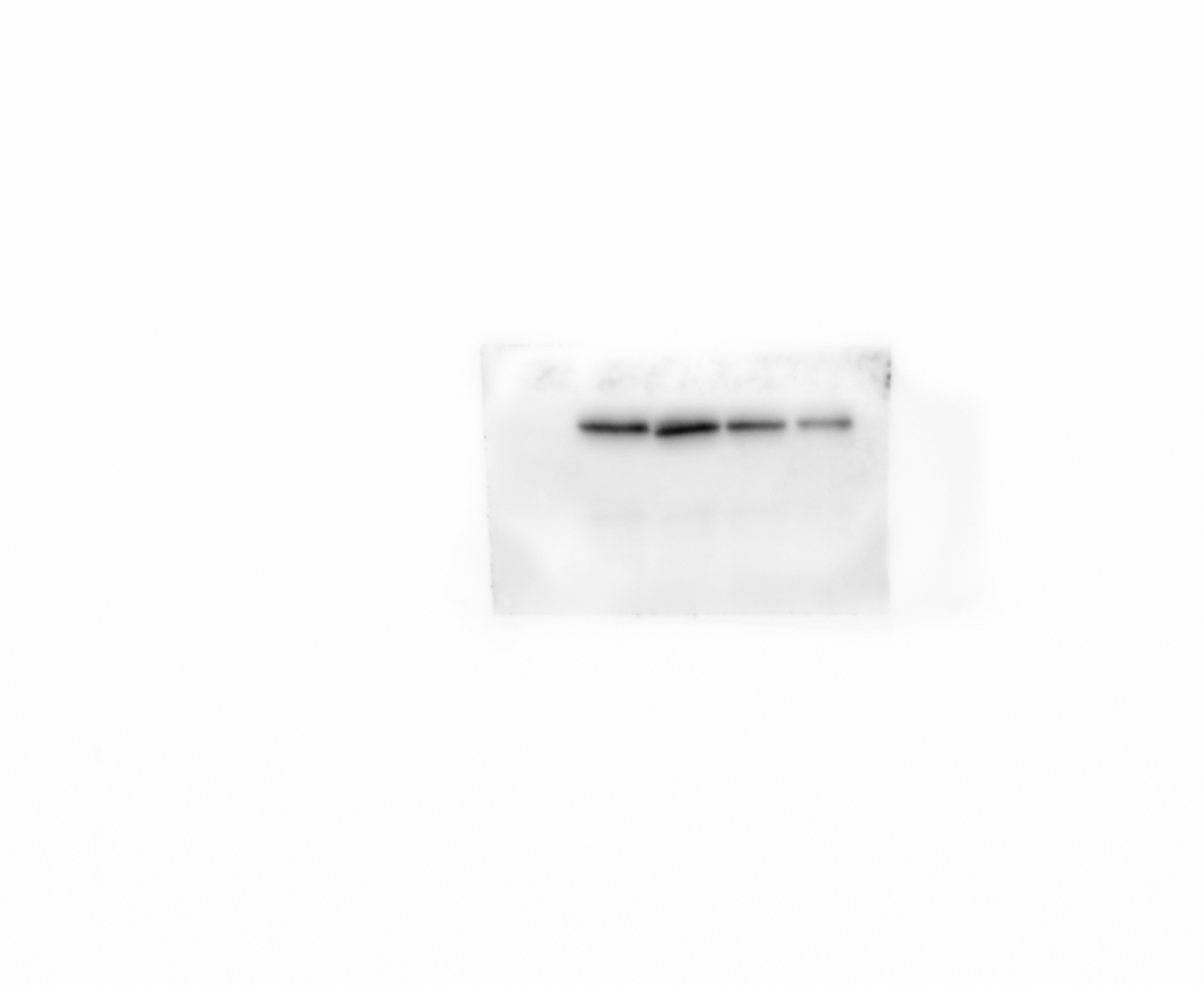

Supplement: Supplementary file 7 [file DataSheet6.ZIP › WB-fig 6/PI3K/202012 18_181350_0.5.0_4.tif]

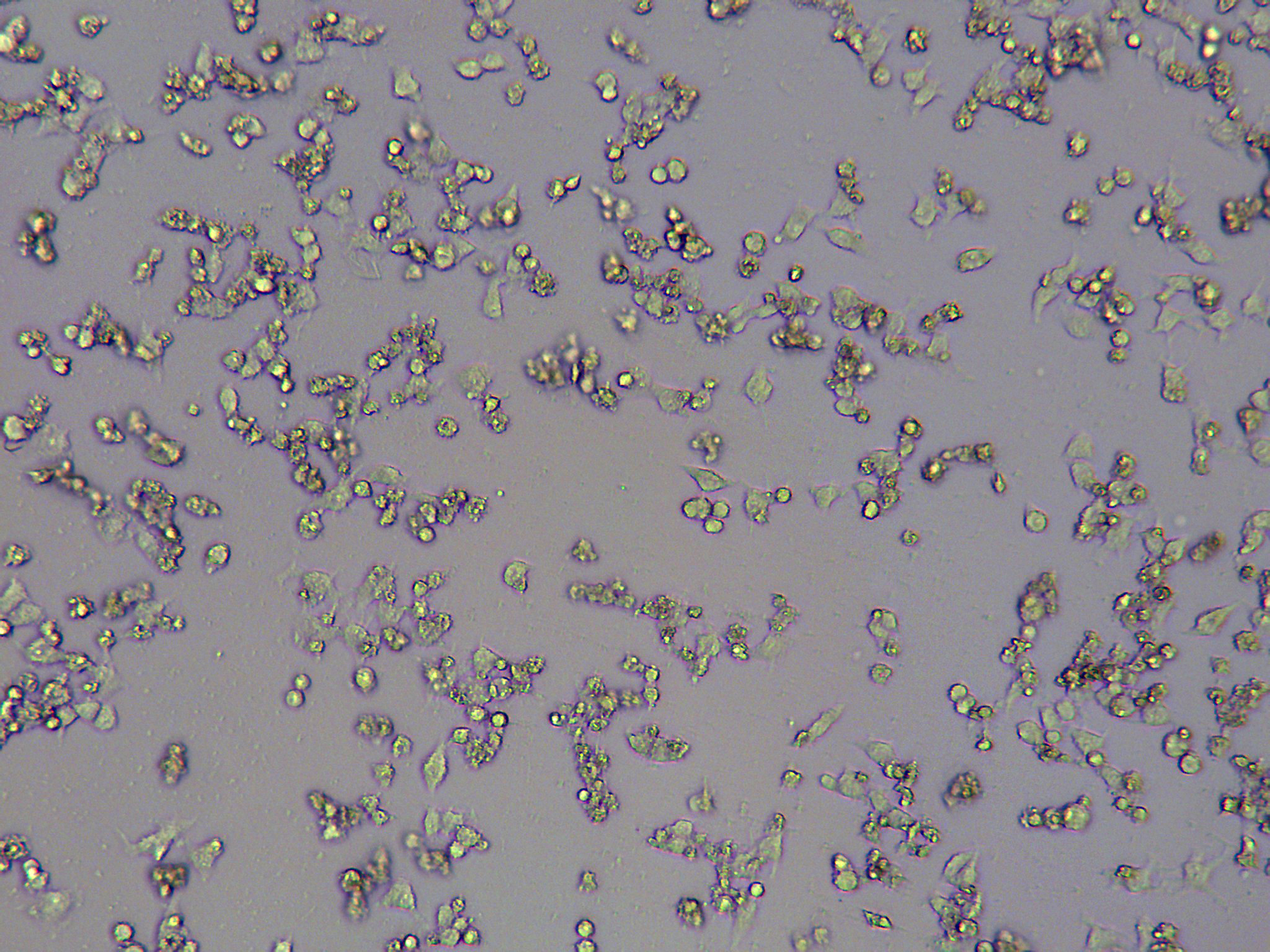

Supplement: Supplementary file 8 [file DataSheet2.ZIP › figure 2/CasKi/1000nm.jpg]

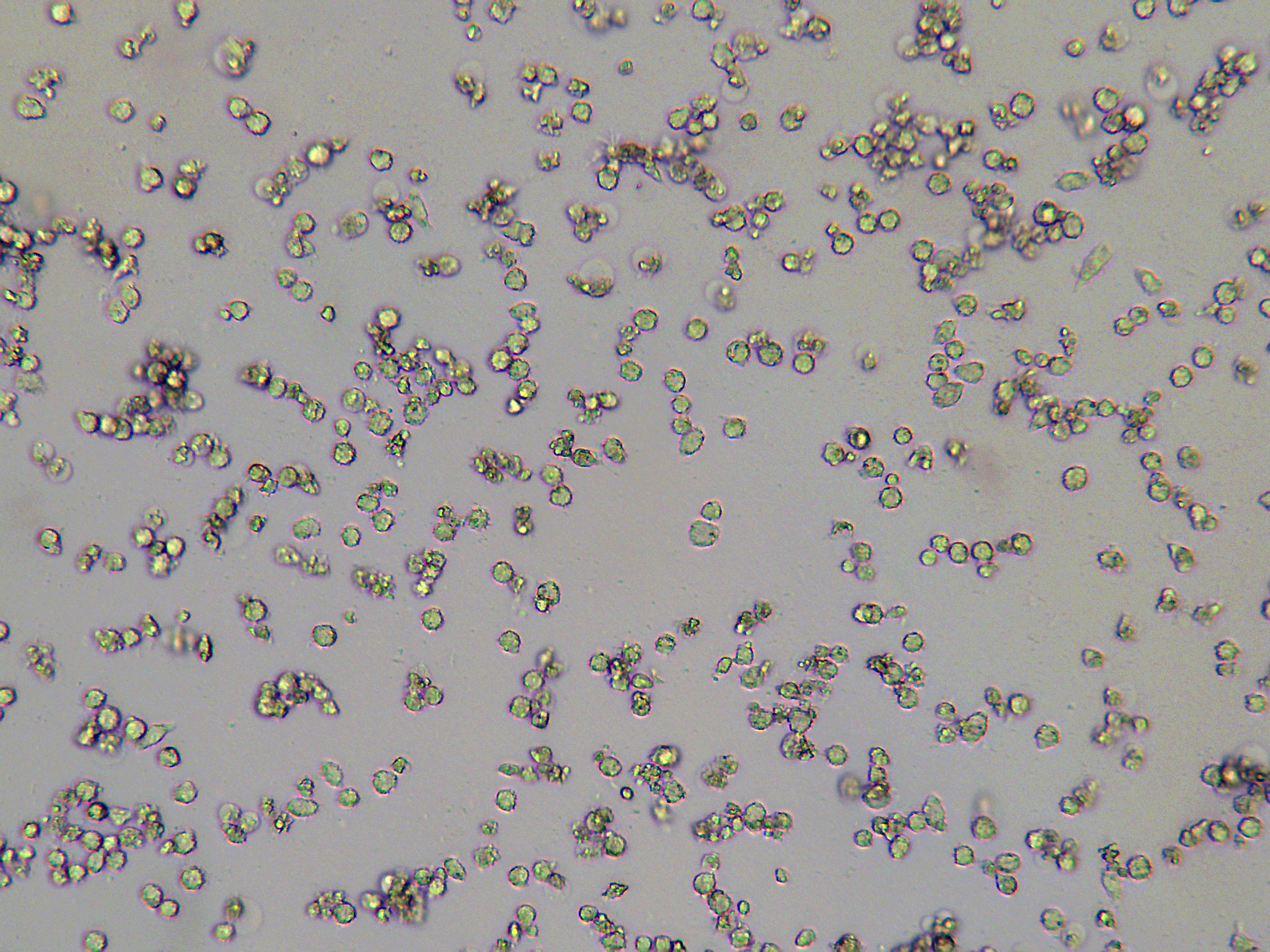

Supplement: Supplementary file 8 [file DataSheet2.ZIP › figure 2/CasKi/1500nm.jpg]

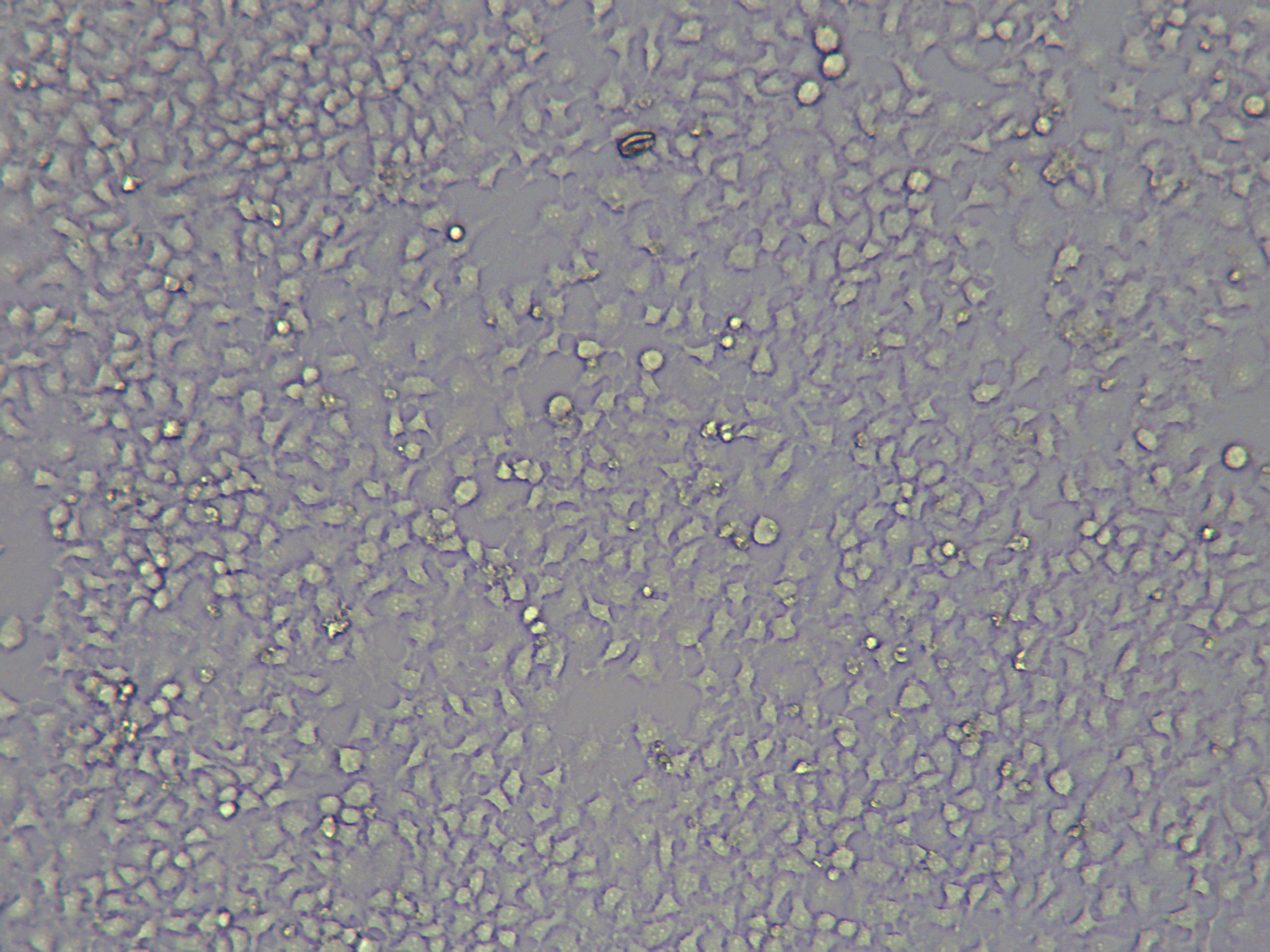

Supplement: Supplementary file 8 [file DataSheet2.ZIP › figure 2/CasKi/500nm.jpg]

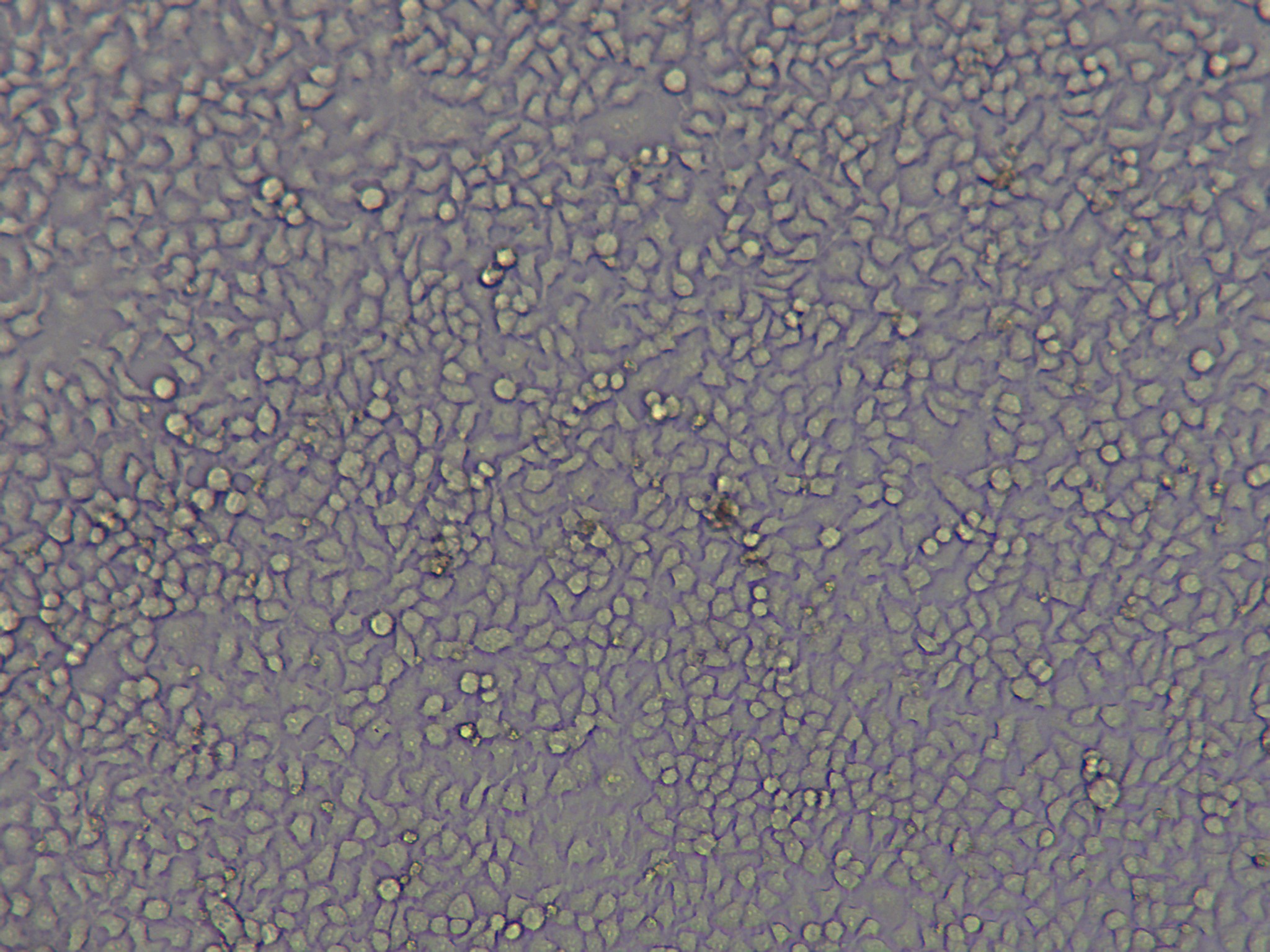

Supplement: Supplementary file 8 [file DataSheet2.ZIP › figure 2/CasKi/control.jpg]

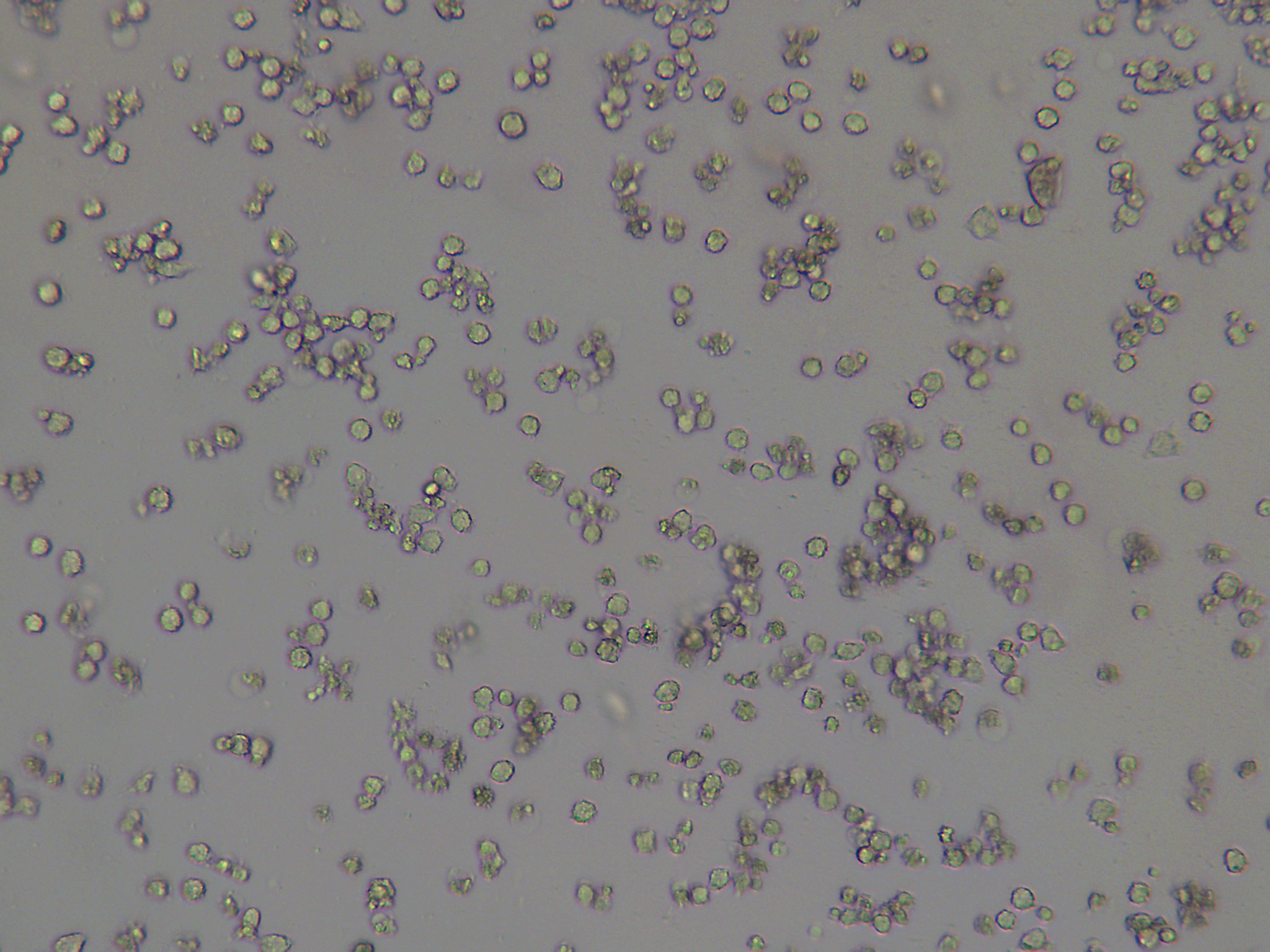

Supplement: Supplementary file 8 [file DataSheet2.ZIP › figure 2/Hela/1000nm.jpg]

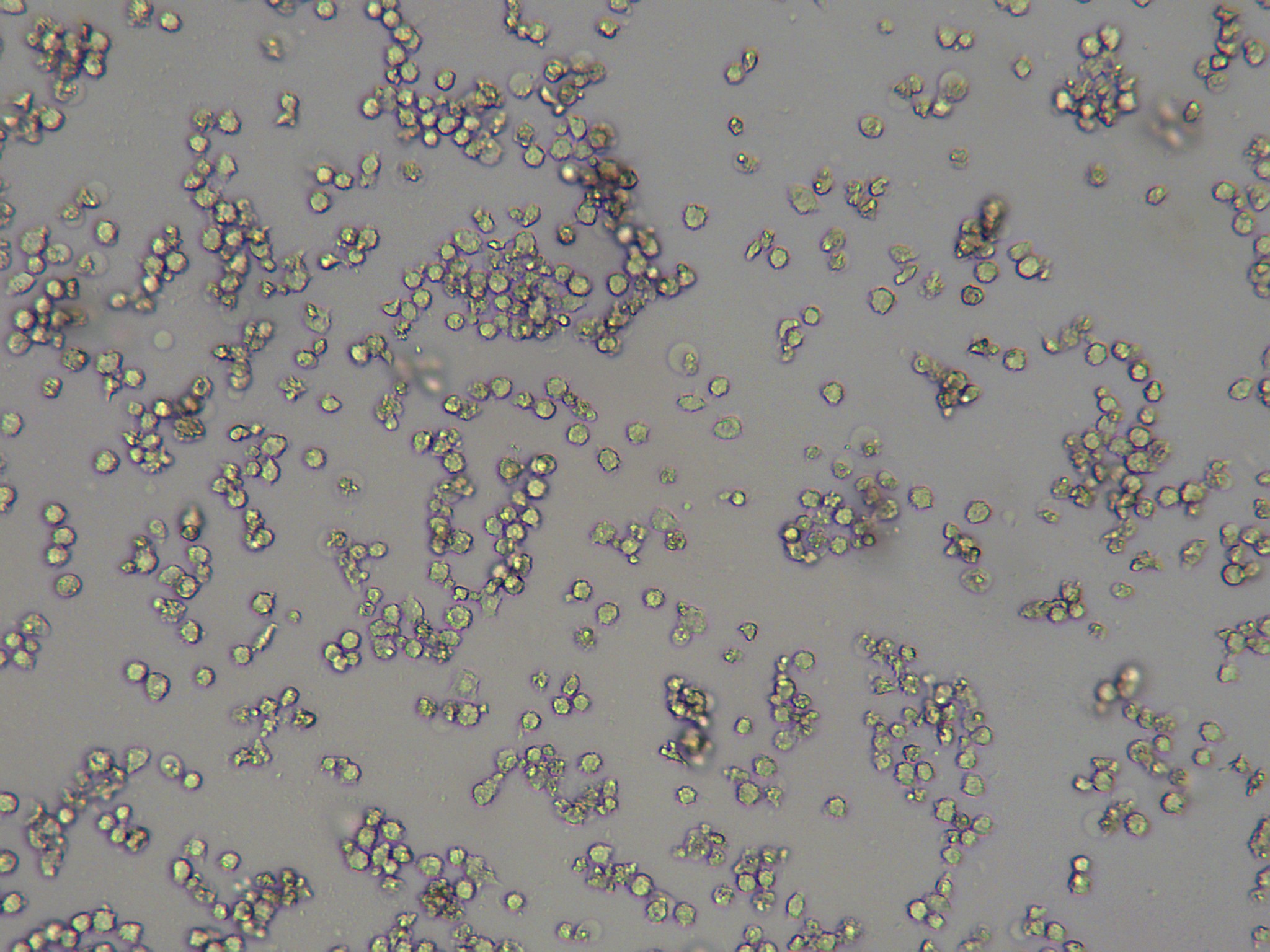

Supplement: Supplementary file 8 [file DataSheet2.ZIP › figure 2/Hela/1500 nm.jpg]

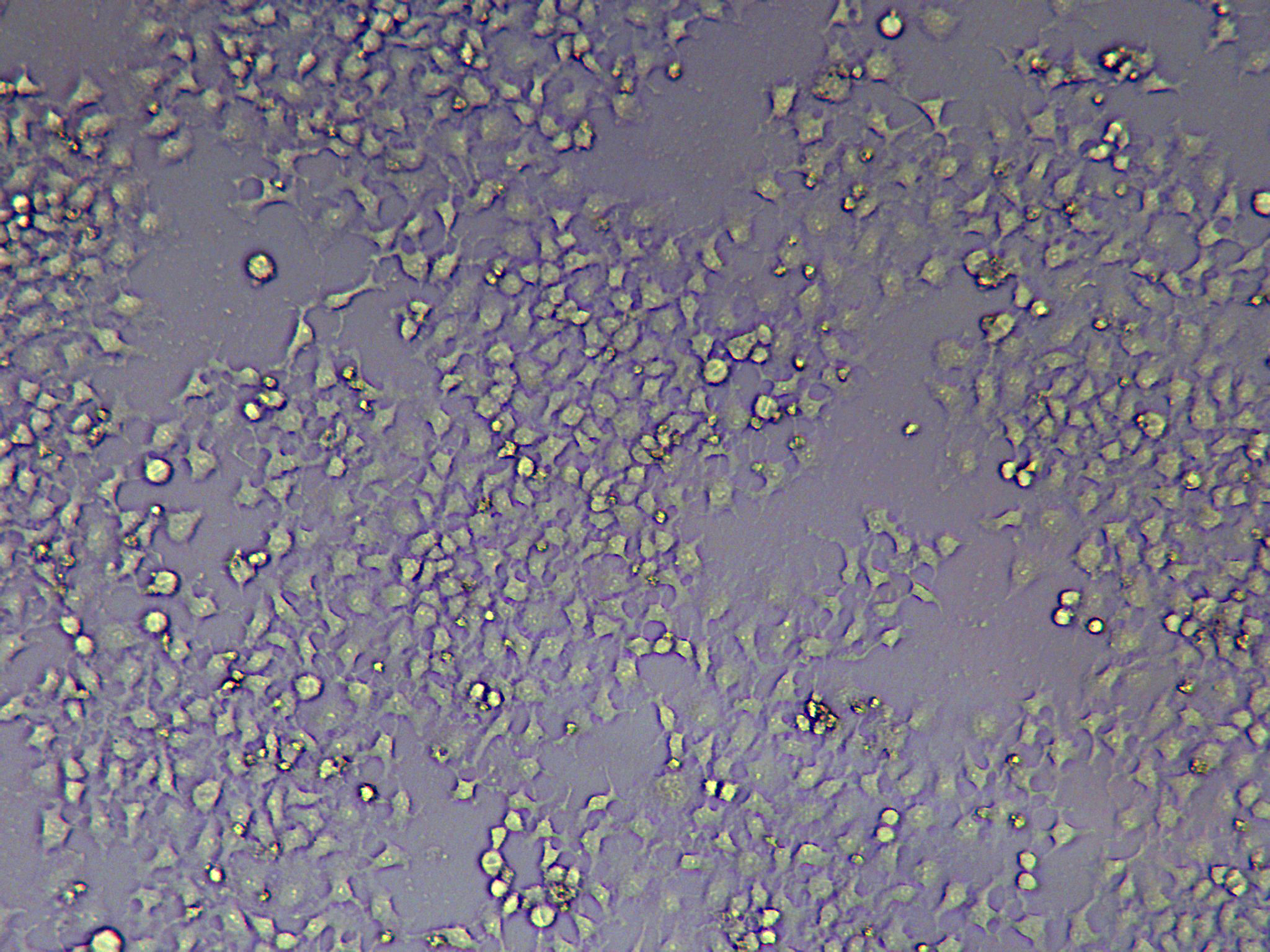

Supplement: Supplementary file 8 [file DataSheet2.ZIP › figure 2/Hela/500 nm.jpg]

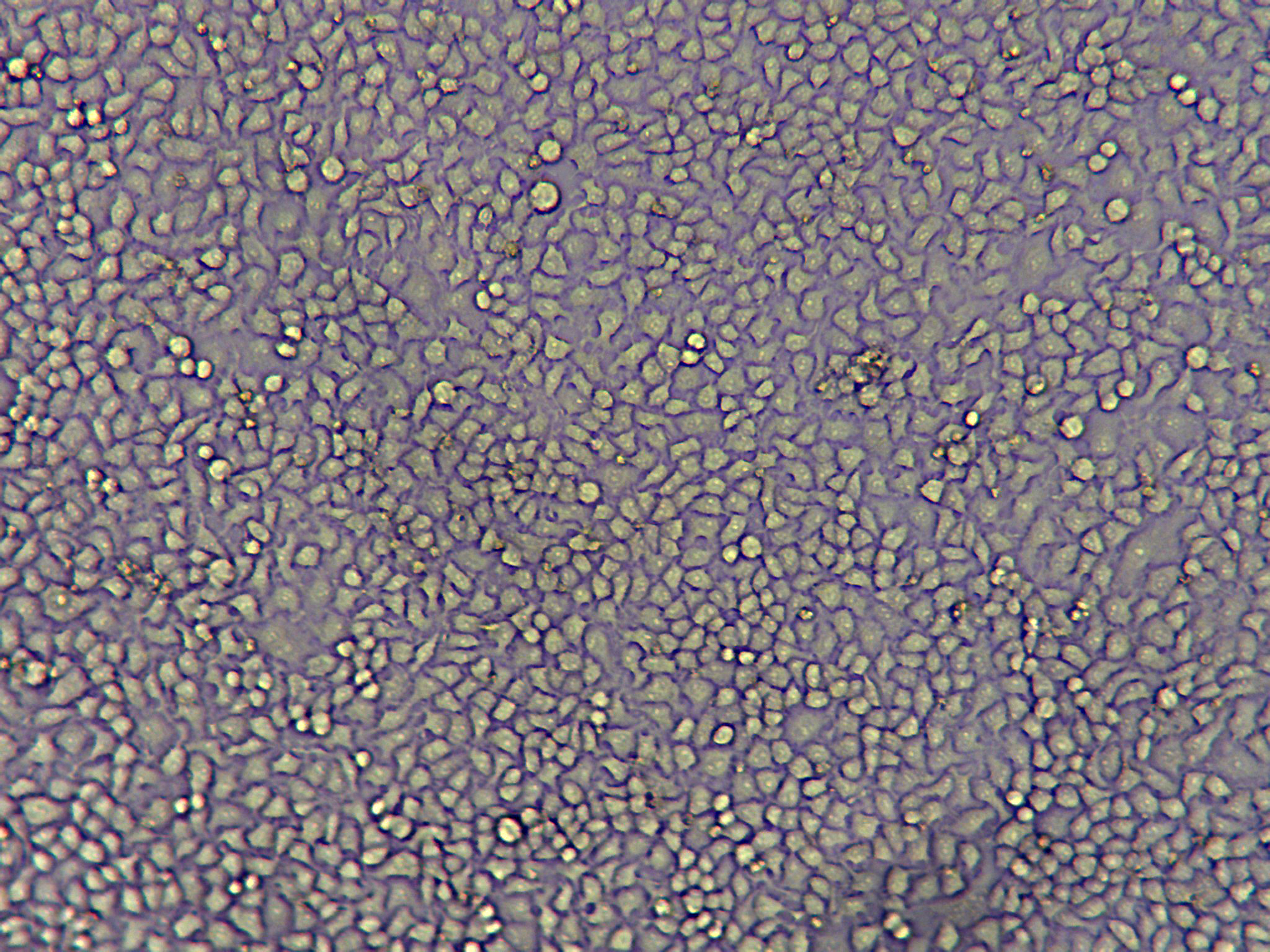

Supplement: Supplementary file 8 [file DataSheet2.ZIP › figure 2/Hela/control.jpg]

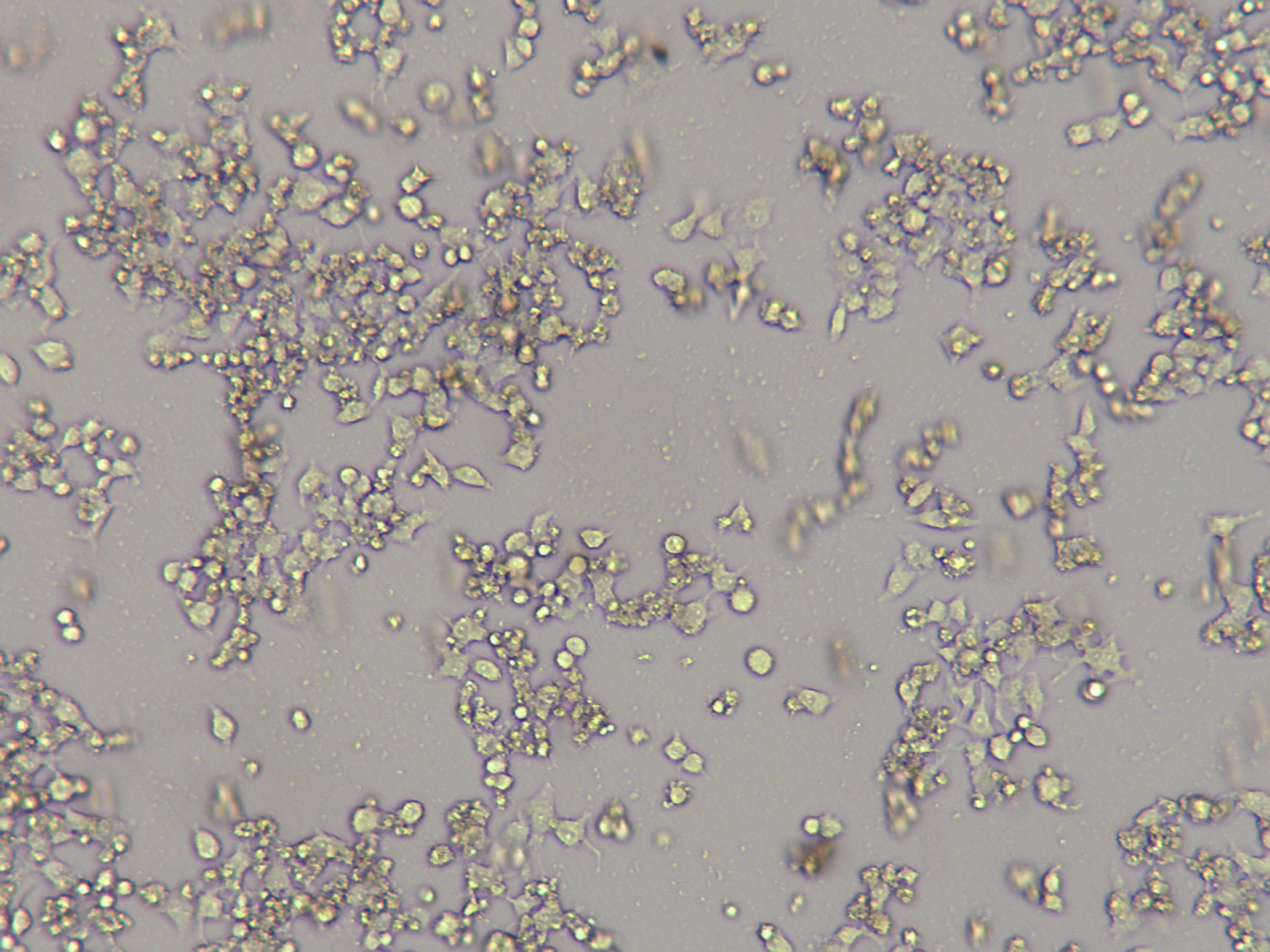

Supplement: Supplementary file 8 [file DataSheet2.ZIP › figure 2/Siha/1000nm.jpg]

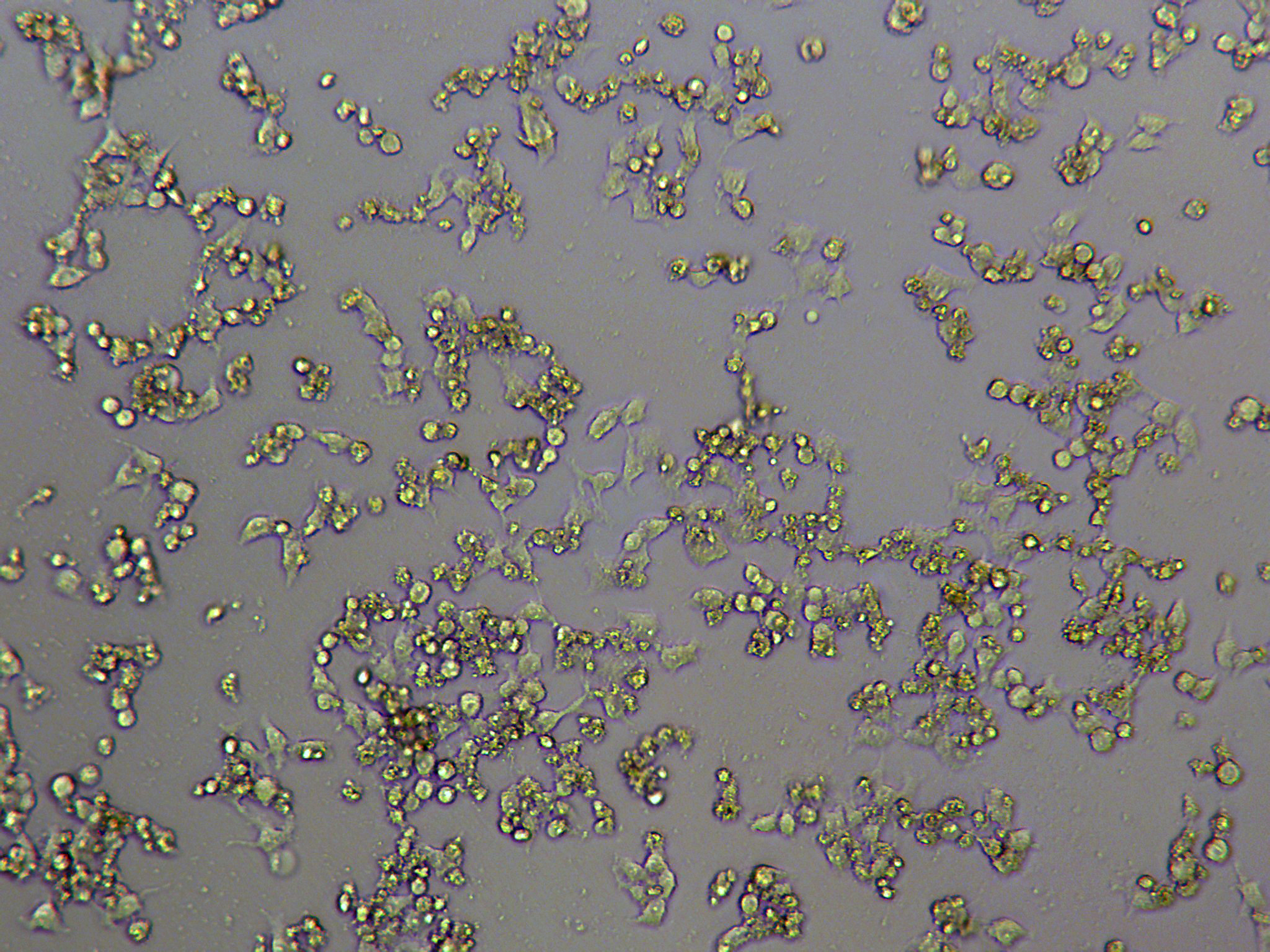

Supplement: Supplementary file 8 [file DataSheet2.ZIP › figure 2/Siha/1500nm.jpg]

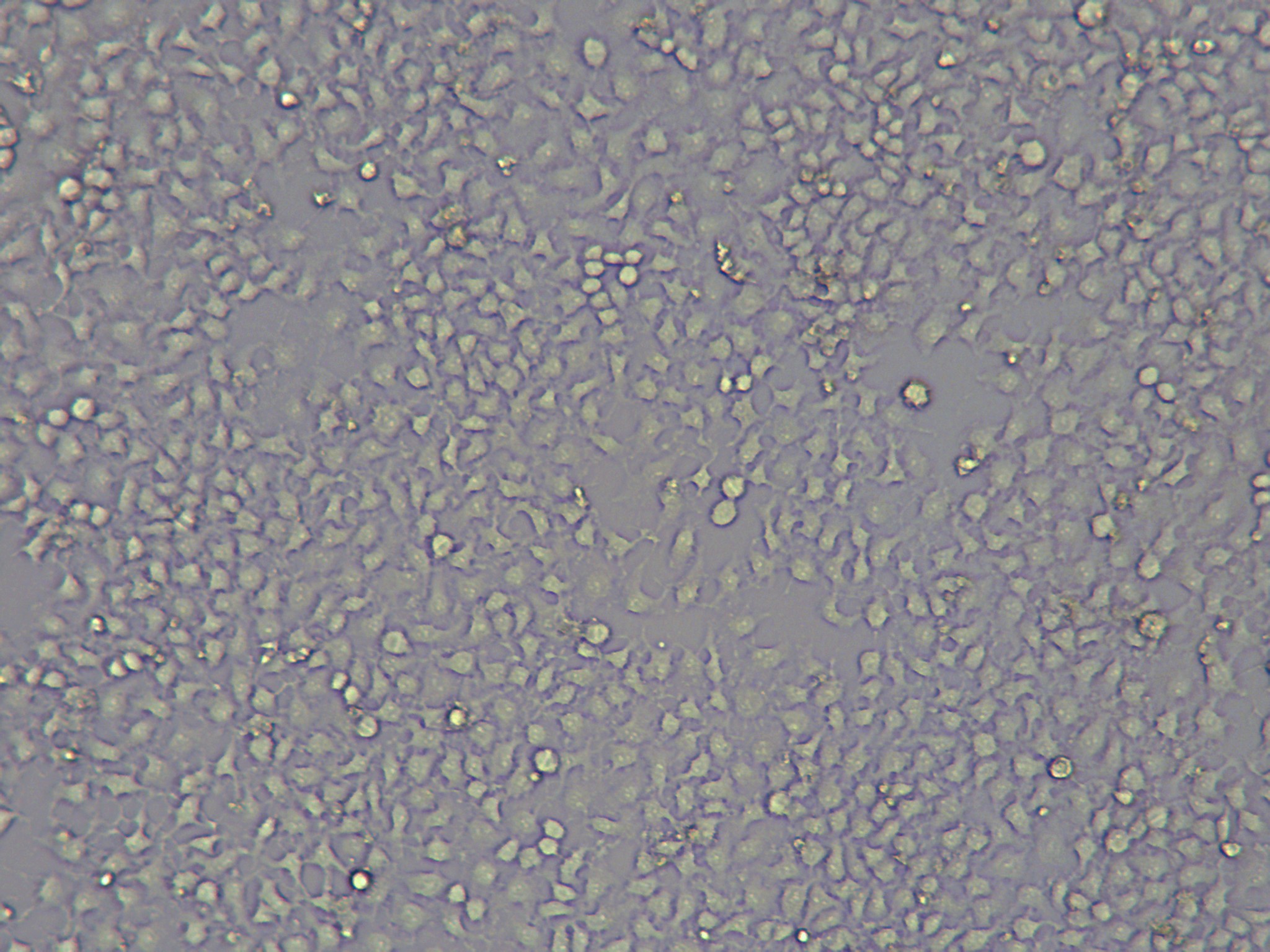

Supplement: Supplementary file 8 [file DataSheet2.ZIP › figure 2/Siha/500nm.jpg]

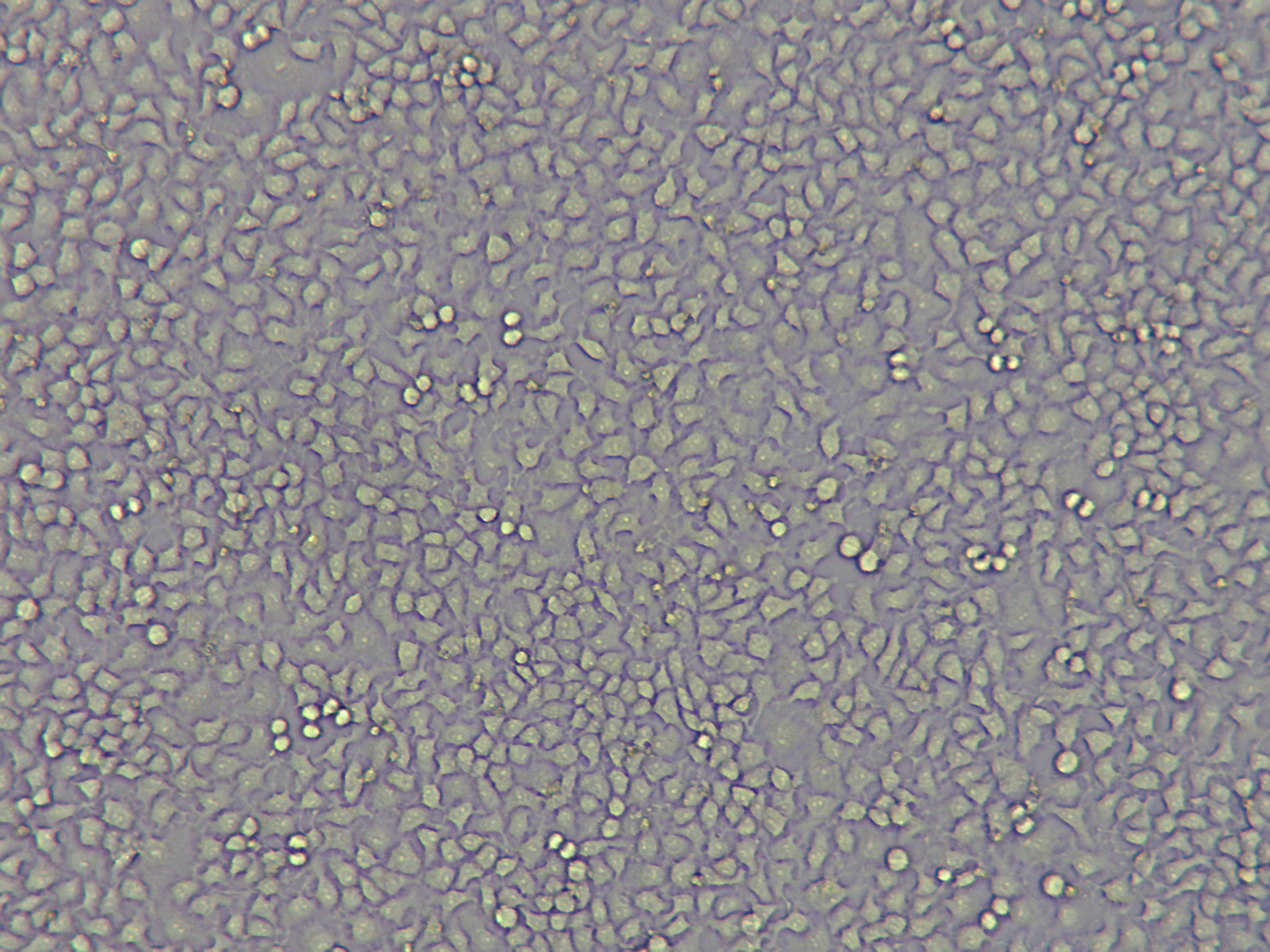

Supplement: Supplementary file 8 [file DataSheet2.ZIP › figure 2/Siha/control;.jpg]

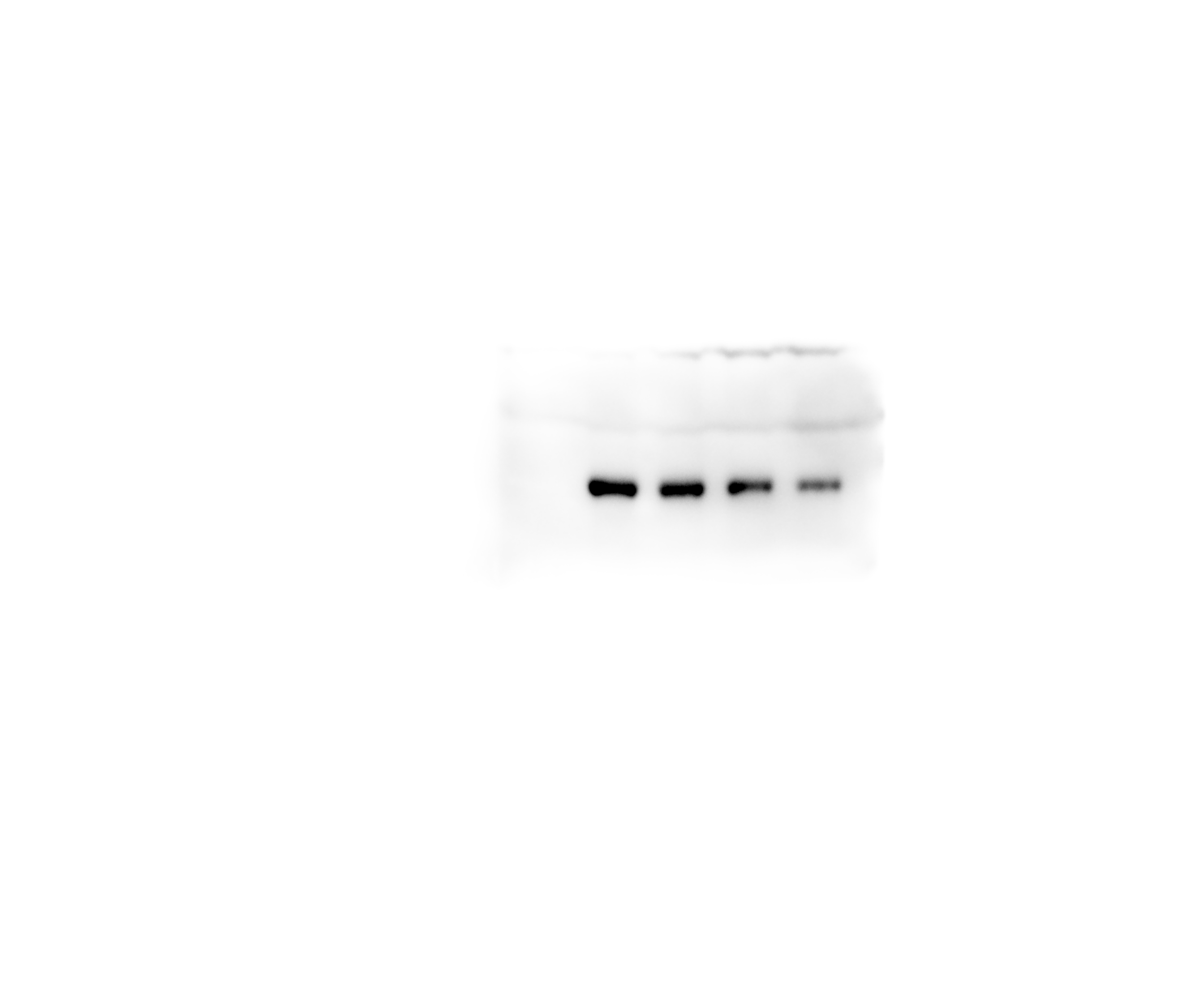

Supplement: Supplementary file 9 [file DataSheet5.ZIP › WB-fig 5/bcl-xl/20190803_172736_0.12.0_ 1.tif]

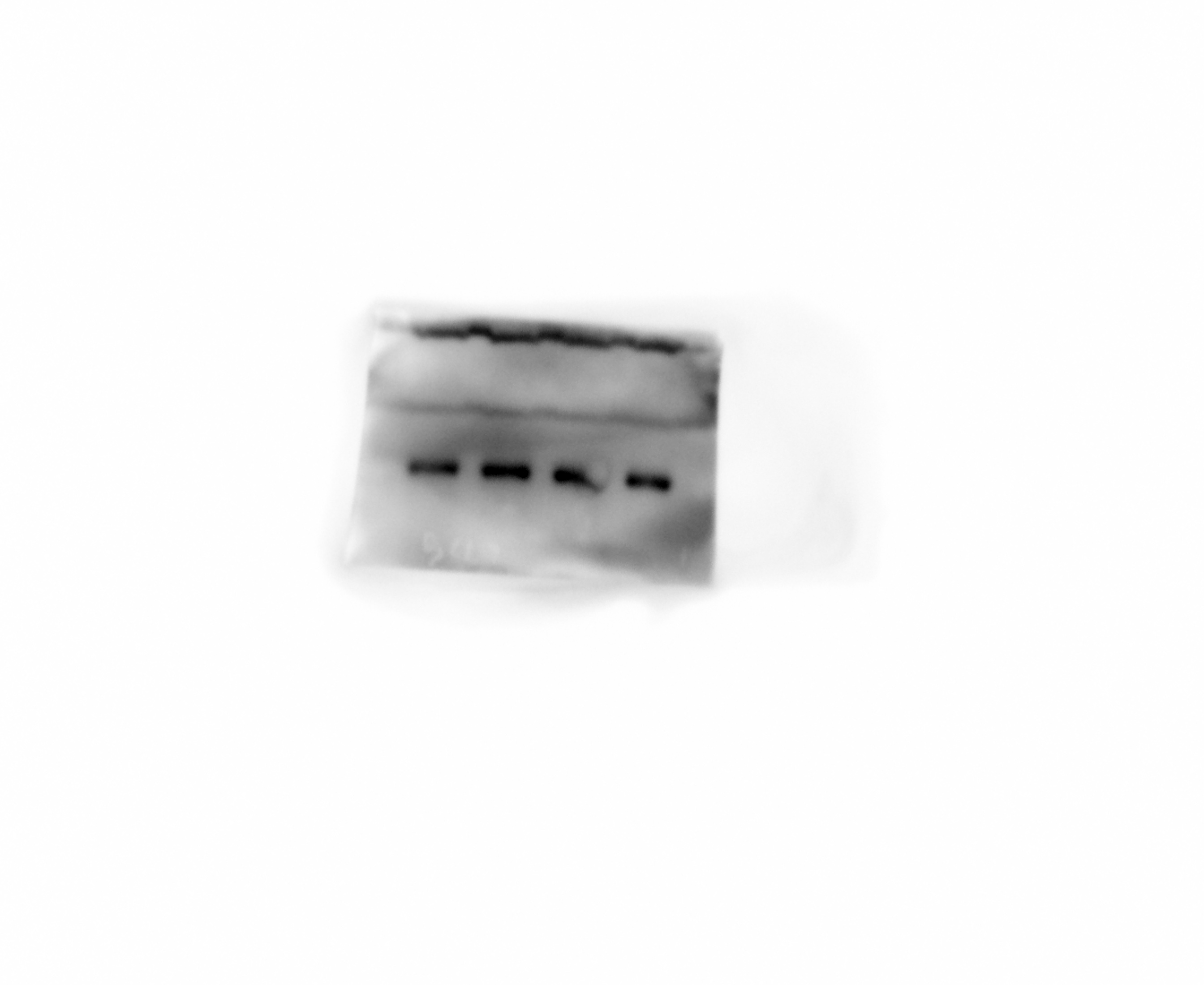

Supplement: Supplementary file 9 [file DataSheet5.ZIP › WB-fig 5/bcl2/20190803_0173052_0.5.0_1.jpg]

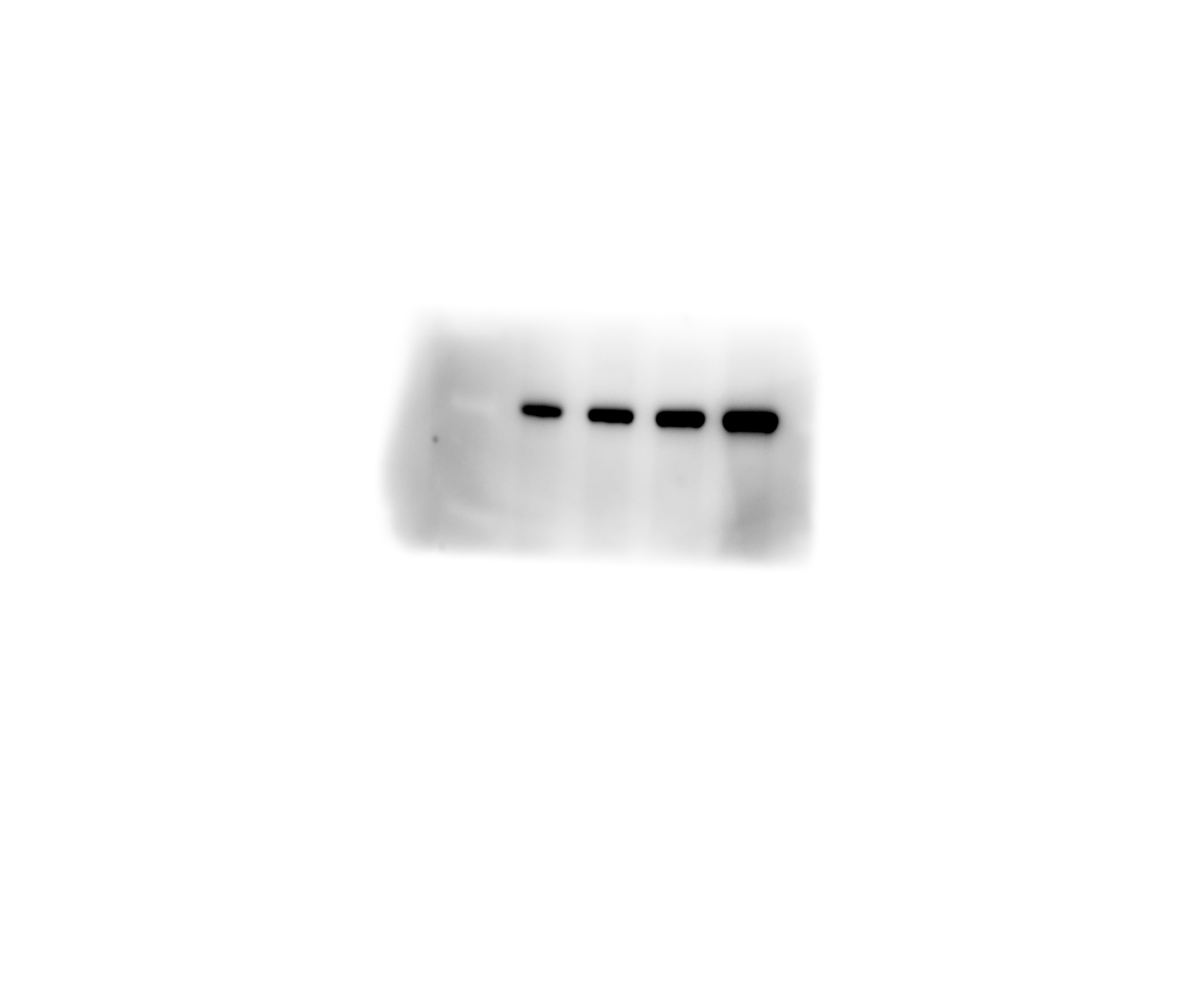

Supplement: Supplementary file 9 [file DataSheet5.ZIP › WB-fig 5/cas 3/20190803_174038 _0.10.0_3.tif]

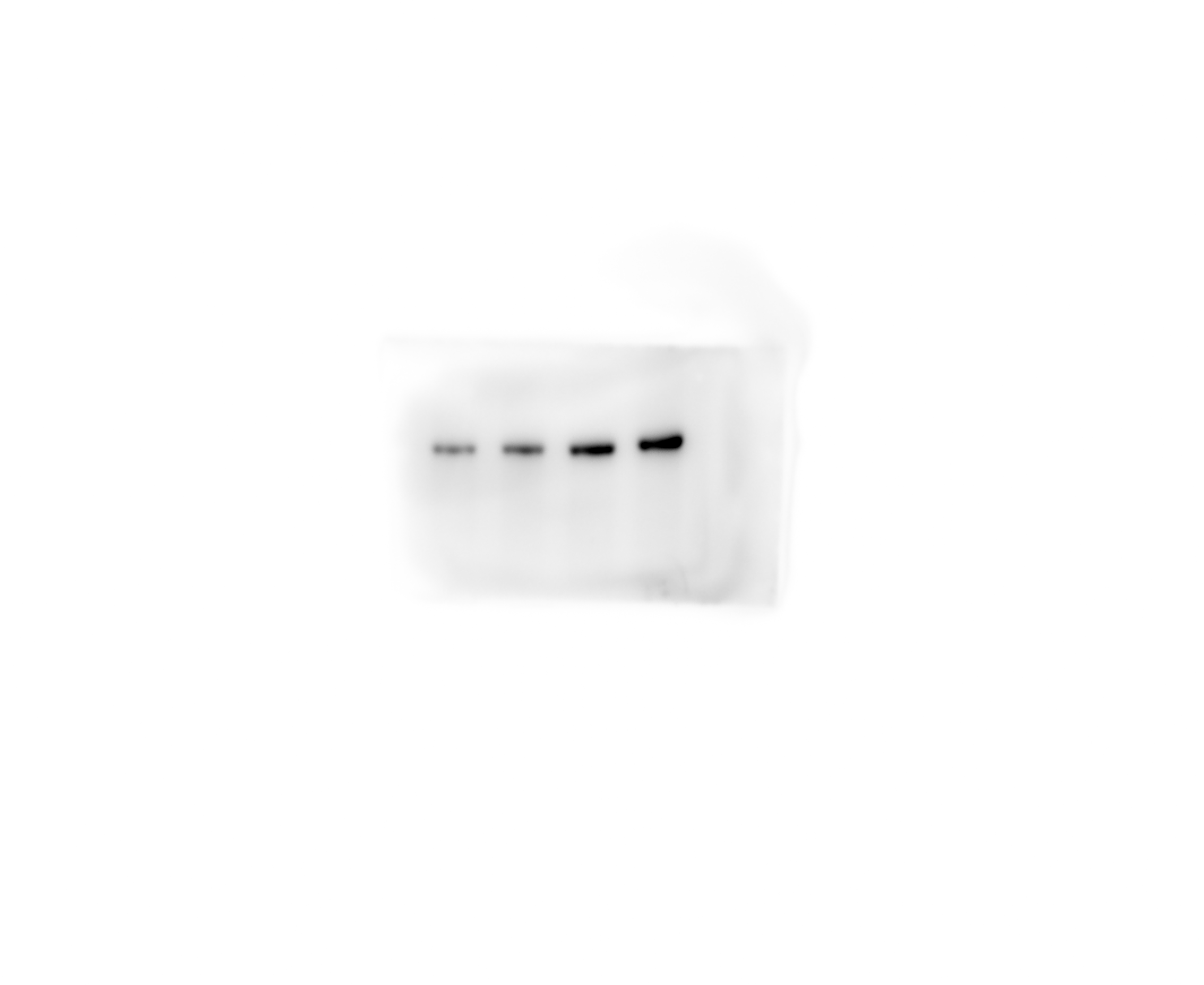

Supplement: Supplementary file 9 [file DataSheet5.ZIP › WB-fig 5/cas 9/20190803_174742_0.5.0_ 3.tif]

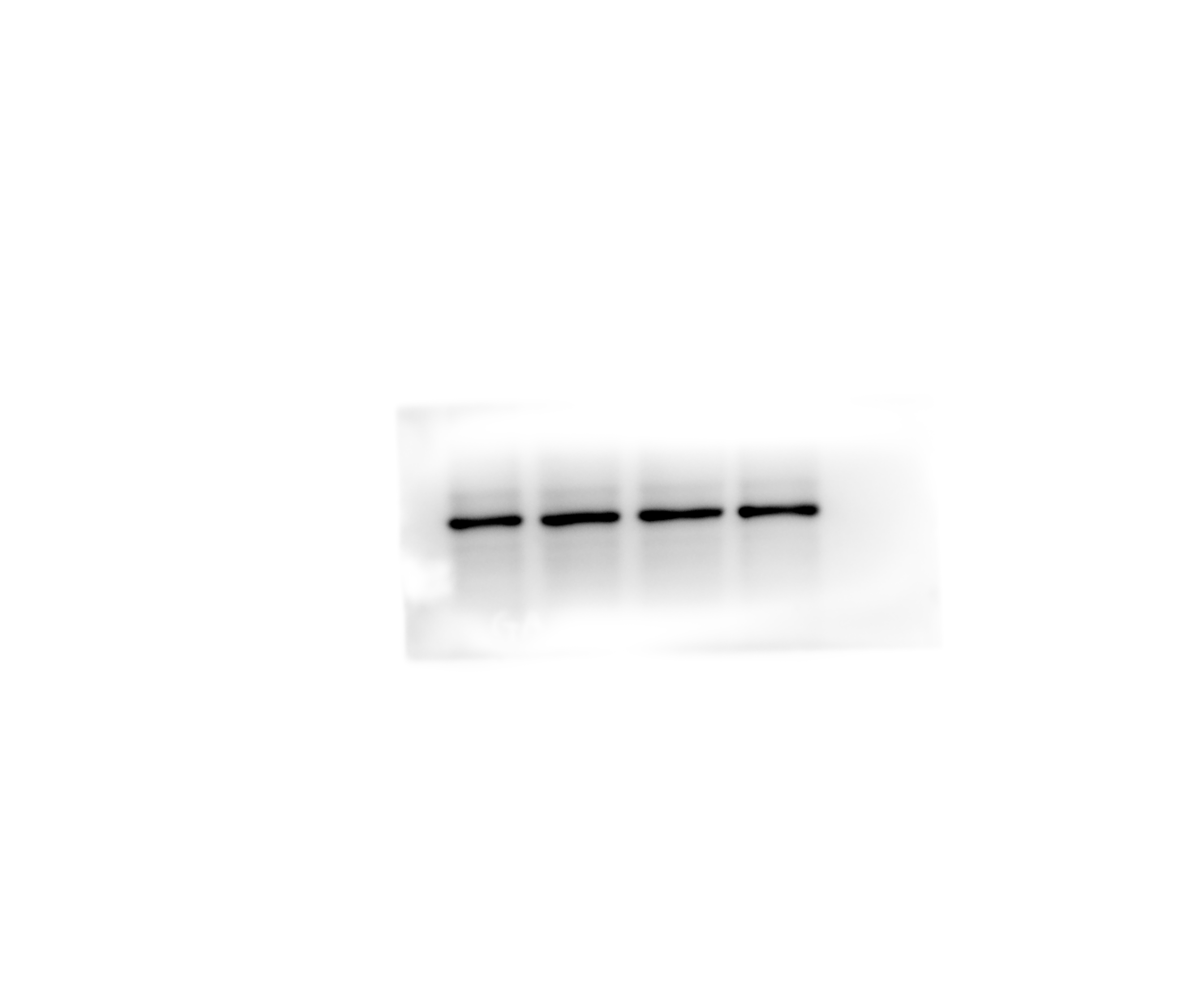

Supplement: Supplementary file 9 [file DataSheet5.ZIP › WB-fig 5/GAPDH/20190731_194617_ 0.25.0_1.tif]

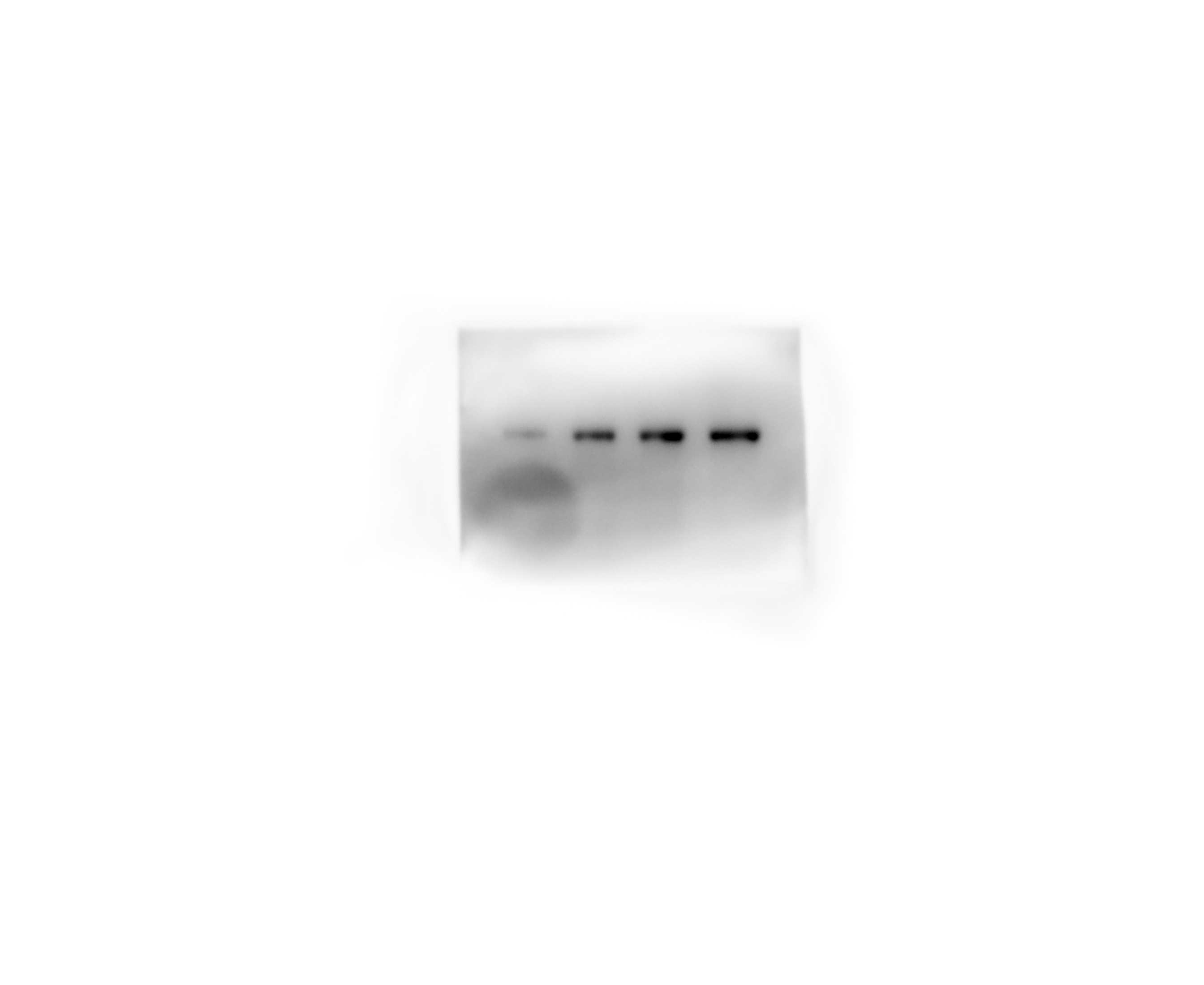

Supplement: Supplementary file 9 [file DataSheet5.ZIP › WB-fig 5/PARP/20190803_1745 29_0.5.0_1.tif]

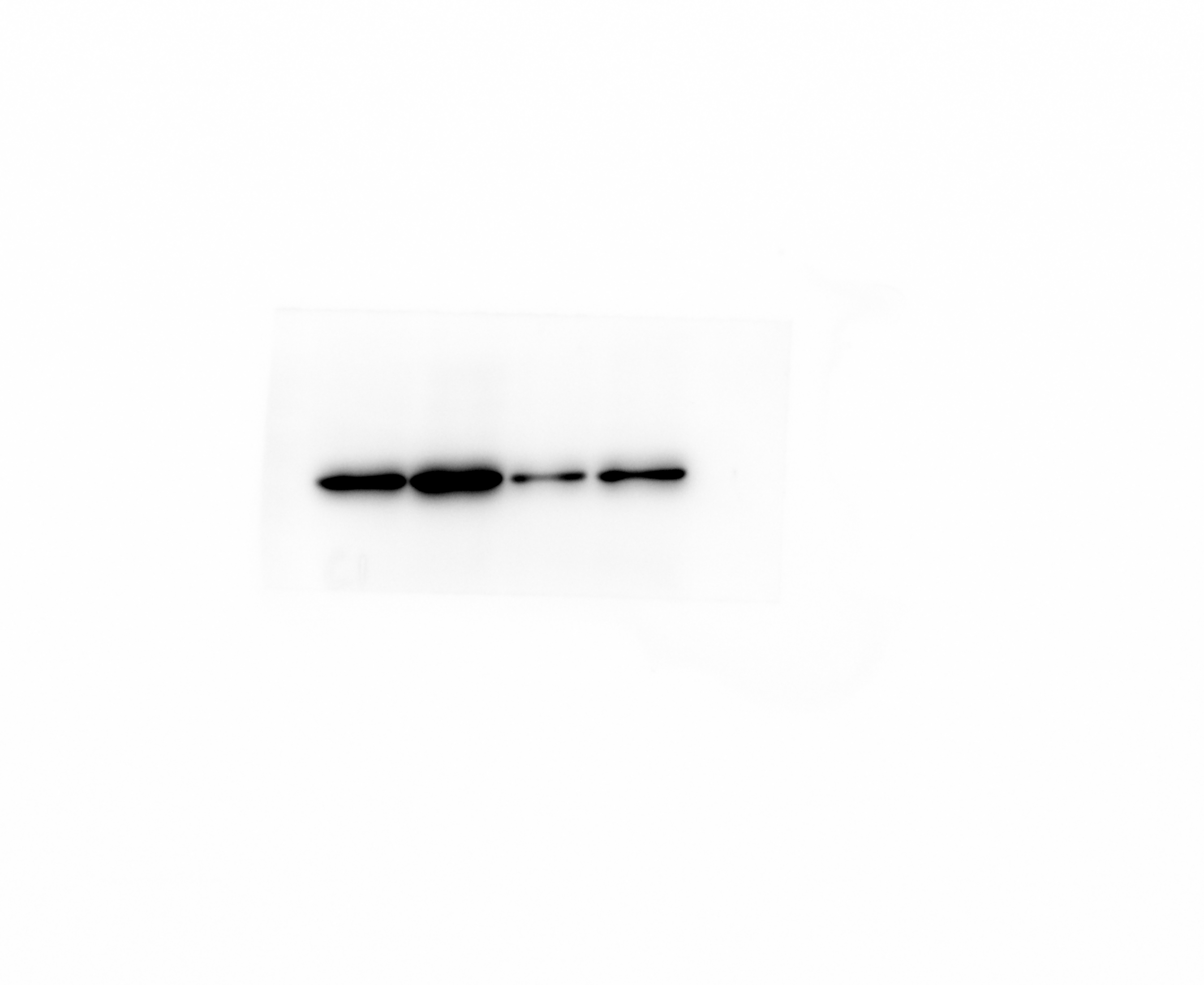

Supplement: Supplementary file 10 [file DataSheet7.ZIP › WB-fig 7/casp3/20200624_100 527_0.10.0_4.tif]

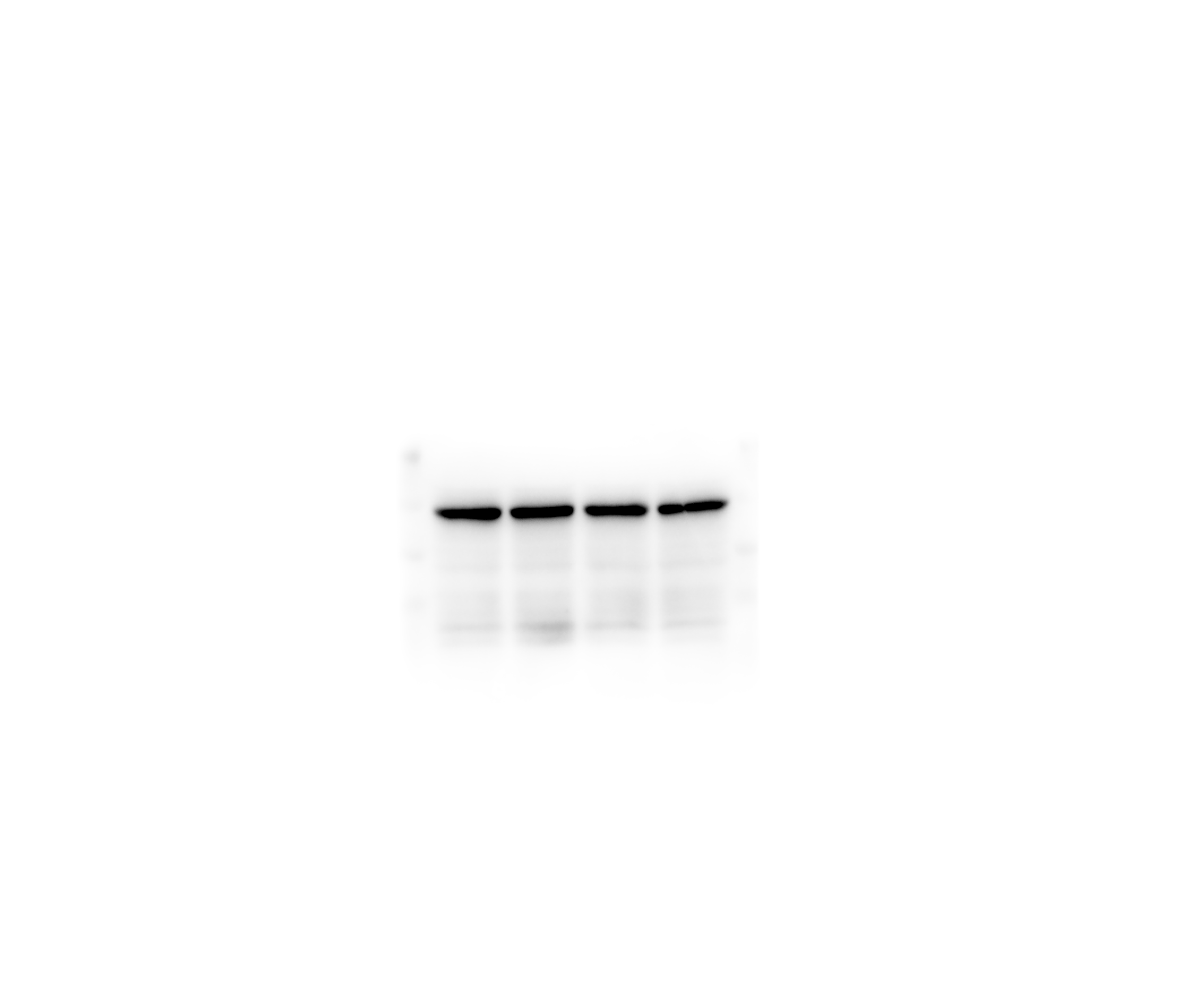

Supplement: Supplementary file 10 [file DataSheet7.ZIP › WB-fig 7/GAPDH/202012 11_142854_0.5.0_4.tif]

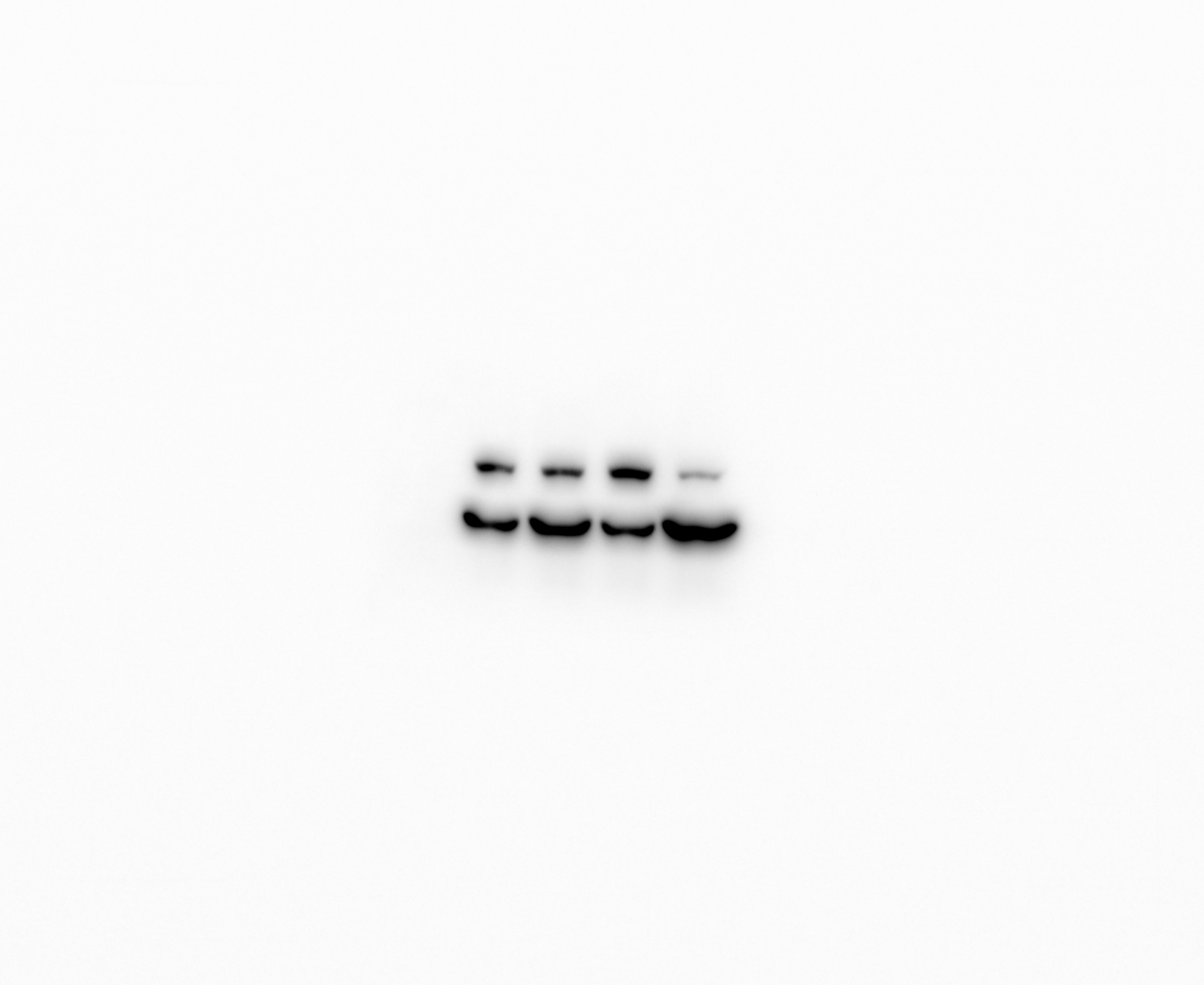

Supplement: Supplementary file 10 [file DataSheet7.ZIP › WB-fig 7/LC3B/20200703_135039_0. 15. 0_4.tif]

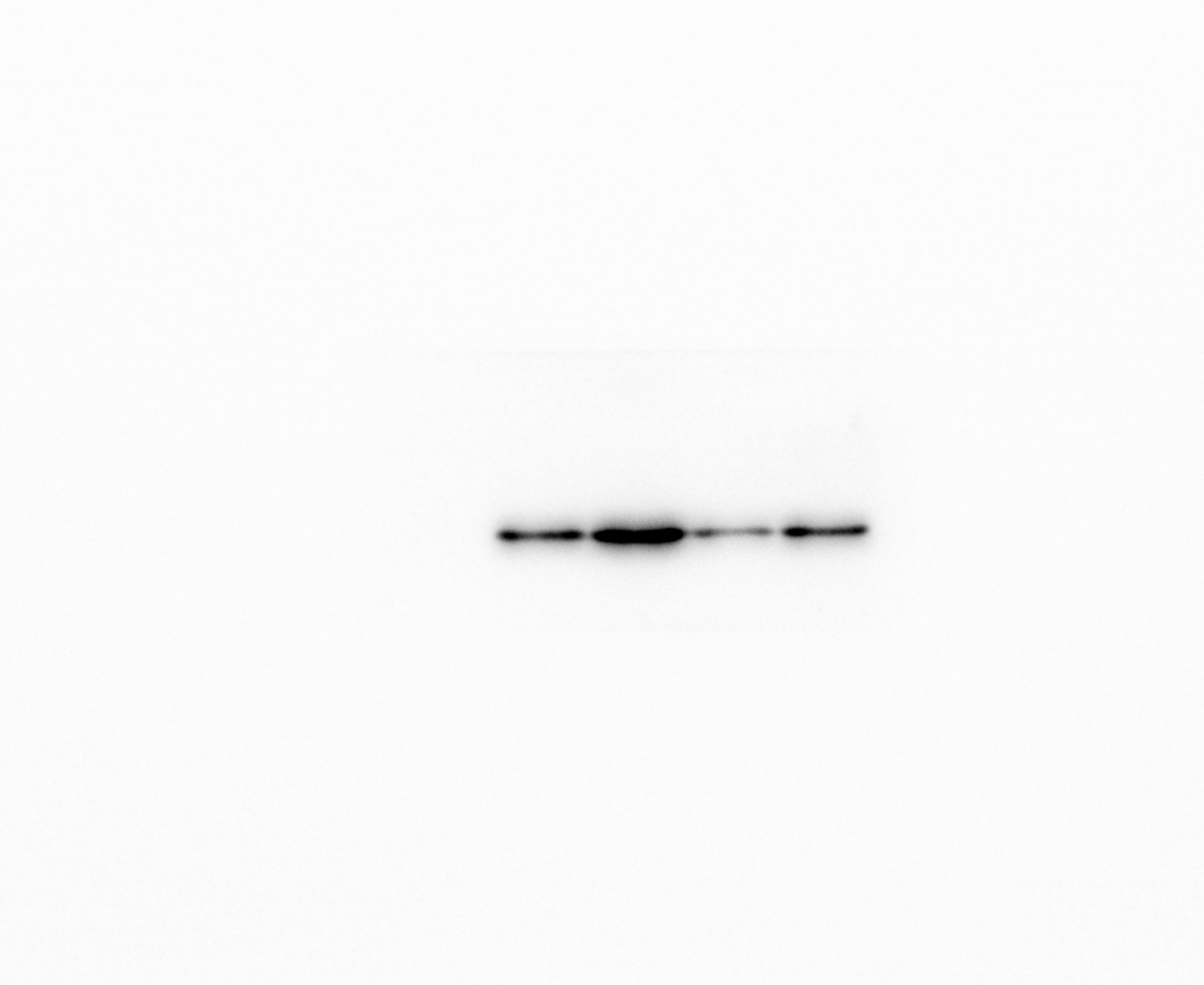

Supplement: Supplementary file 10 [file DataSheet7.ZIP › WB-fig 7/PARP/2020 0624_104134_0.5.0_3.tif]
