# Supplementary material for: Glucocappasalin Induces G2/M-Phase Arrest, Apoptosis, and Autophagy Pathways by Targeting CDK1 and PLK1 in Cervical Carcinoma Cells
Source: Front Pharmacol. 2021 May 20;12:671138. doi: 10.3389/fphar.2021.671138 (PMC8172611; doi:10.3389/fphar.2021.671138)
Supplement: Supplementary file 3 [file DataSheet8.ZIP › supplementary materials/Chinese-Certificate of Laboratory Animals’ Ethics.pdf]

成都大学实验动物伦理的审查意见

时政 申请的 2018 年国家自然科学基金项目“CDK1/PLK1 双靶点介导天然产物 glucocappasalin 诱导宫颈癌细胞自噬和凋亡分子机制研究”所涉及的实验动物有关材料，经成都大学伦理委员会审查，认为该项目符合实验动物伦理学的要求，同意申报。

成都大学伦理委员会

2018 年 3 月 6 日

成都大学伦理委员会

由 扫描全能王 扫描创建
