# Supplementary material for: Glucocappasalin Induces G2/M-Phase Arrest, Apoptosis, and Autophagy Pathways by Targeting CDK1 and PLK1 in Cervical Carcinoma Cells
Source: Front Pharmacol. 2021 May 20;12:671138. doi: 10.3389/fphar.2021.671138 (PMC8172611; doi:10.3389/fphar.2021.671138)
Supplement: Supplementary file 3 [file DataSheet8.ZIP › supplementary materials/CWS_Editorial_Certificate.pdf]

Charlesworth Author Services

EDITORIAL CERTIFICATE

This document certifies that the manuscript below was edited for correct English language usage, grammar, punctuation and spelling by qualified native English speaking editors at Charlesworth Author Services.

Paper Title:

Glucocappasalin targeting both CDK1/PLK1 and inducing G2/M-phase arrest, apoptosis, and autophagy pathways in cervical carcinoma cells

Author:

xuel yan

Date certificate issued:

April 21, 2021

cwauthors.com
