# Supplementary material for: Glucocappasalin Induces G2/M-Phase Arrest, Apoptosis, and Autophagy Pathways by Targeting CDK1 and PLK1 in Cervical Carcinoma Cells
Source: Front Pharmacol. 2021 May 20;12:671138. doi: 10.3389/fphar.2021.671138 (PMC8172611; doi:10.3389/fphar.2021.671138)
Supplement: Supplementary file 3 [file DataSheet8.ZIP › supplementary materials/English-Certificate of Laboratory Animals’ Ethics.docx]

**Certificate of Laboratory Animals’ Ethics of Chengdu University**

**Project：**Molecular mechanism of natural product glucocappasalin induced autophagy and apoptosis in cervical cancer by targeting CDK1/PLK1. **Principle investigator:** Zheng Shi. The animal experiment is under the certify assessment by the Laboratory animal ethics committee Chengdu University.

Date：March 6, 2018
